# *Stachybotrys chartarum*—A Hidden Treasure: Secondary Metabolites, Bioactivities, and Biotechnological Relevance

**DOI:** 10.3390/jof8050504

**Published:** 2022-05-12

**Authors:** Sabrin R. M. Ibrahim, Hani Choudhry, Amer H. Asseri, Mahmoud A. Elfaky, Shaimaa G. A. Mohamed, Gamal A. Mohamed

**Affiliations:** 1 Department of Chemistry, Preparatory Year Program, Batterjee Medical College, Jeddah 21442, Saudi Arabia; 2 Department of Pharmacognosy, Faculty of Pharmacy, Assiut University, Assiut 71526, Egypt; 3 Biochemistry Department, Faculty of Science, King Abdulaziz University, Jeddah 21589, Saudi Arabia; hchoudhry@kau.edu.sa (H.C.); ahasseri@kau.edu.sa (A.H.A.); 4 Center for Artificial Intelligence in Precision Medicines, King Abdulaziz University, Jeddah 21589, Saudi Arabia; melfaky@kau.edu.sa; 5 Department of Natural Products and Alternative Medicine, Faculty of Pharmacy, King Abdulaziz University, Jeddah 21589, Saudi Arabia; gahussein@kau.edu.sa; 6 Faculty of Dentistry, British University, El Sherouk City, Suez Desert Road, Cairo 11837, Egypt; shaimaag1973@gmail.com

**Keywords:** fungi, *Stachybotrys chartarum*, Stachybotriaceae, metabolites, phenylspirodrimanes, bioactivity, biotechnology

## Abstract

Fungi are renowned as a fountainhead of bio-metabolites that could be employed for producing novel therapeutic agents, as well as enzymes with wide biotechnological and industrial applications. *Stachybotrys chartarum* (black mold) (Stachybotriaceae) is a toxigenic fungus that is commonly found in damp environments. This fungus has the capacity to produce various classes of bio-metabolites with unrivaled structural features, including cyclosporins, cochlioquinones, atranones, trichothecenes, dolabellanes, phenylspirodrimanes, xanthones, and isoindoline and chromene derivatives. Moreover, it is a source of various enzymes that could have variable biotechnological and industrial relevance. The current review highlights the formerly published data on *S. chartarum*, including its metabolites and their bioactivities, as well as industrial and biotechnological relevance dated from 1973 to the beginning of 2022. In this work, 215 metabolites have been listed and 138 references have been cited.

## 1. Introduction

Fungi are found predominantly in all environments and have substantial roles in preserving eco-balance, diversity, and sustainability [1,2,3]. They have demonstrated a wide array of industrial and biotechnological potentials [1,4,5,6]. Additionally, they are an important source of metabolites with unique chemical skeletons that have the potential for discovering drugs of clinical relevance [7,8,9,10,11,12]. This was evident by many reported fungi-derived drugs that are in use, for example camptothecin, cyclosporine, paclitaxel, torreyanic acid, compactin, vincristine, lovastatin, and cytarabine [13,14,15]. Additionally, they are a pool of new scaffolds, which can be further modified to achieve the needed action [16]. Many of these metabolites can be obtained in considerable amounts and at a feasible cost through fermentation utilizing genetically modified or wild type fungi [15]. Regardless of the remarkable advancement in discovering drugs that provide treatment for most ailments, epidemics, and infections, novel drugs are requested to counter the reported resistance of some diseases and infections to the existing drugs [17,18,19]. Despite the biodiversity of the fungi kingdom, only a limited number of fungi have been explored for their capacity to produce bioactive metabolites.

*Stachybotrys* *chartarum* (black mold) (Stachybotriaceae, *S. atra* or *S. alternans*) is a toxigenic fungus that is widely present in the indoor air of buildings or homes which have water damage or sustained flooding from roofs, broken pipes, floor or wall leaks, and condensation. *S. chartarum* a hydrophilic fungus, needing wet conditions for maintaining and initiating its growth. It is also found on gypsum, cellulose-based ceiling tiles, fiberglass, wallpaper, paper products, natural fiber carpets, insulated pipes paper covering, wood and wood paneling, and organic debris, as well as soil, grains, and litter [20,21]. *S. chartarum* is one of the most common pathogenic indoor fungi that is capable of producing mycotoxins, having life-threatening health impacts [21]. Several reports stated that the exposure to this fungus or its mycotoxins through contaminated indoor air and construction material causes severe fungi-mediated sick building illnesses and even human death [20,21,22]. The common symptoms of this illness are fatigue, chest tightness, mucosal irritation, and headache [20]. It can also cause respiratory disorders that range from cough and congestion to more dangerous syndromes including bronchiectasis, alveolitis, and pulmonary fibrosis [23]. It was also found that exposure to this fungus has been related to infants’ pulmonary hemorrhage outbreaks [24]. Further, the exposure to *S. chartarum* in damp environments exceeds the threshold of sensitization in susceptible people [25]. This fungus causes stachybotryotoxicosis in horses and other animals [26]. The fungus’ biosynthesized macrocyclic trichothecenes are considered one of the most powerful inhibitors for protein synthesis. Additionally, it produces biometabolites (e.g. atranones and spirodrimanes), protein factors (e.g. stachylysin and hemolysin), immunosuppressive agents, and proteinases (e.g. serine proteases) that are attributed to pulmonary destruction, hemosiderosis, and hemorrhage [27,28,29]. Most of the reported studies on *S. chartarum* highlighted its pathogenic influences on humans and animals [21,30,31,32].

Surveying the literature revealed no review article that shed light on the positive impact of this important fungus. Therefore, this work reviewed the published studies on this fungus, particularly its metabolites and their bioactivities. In addition, the reported research on *S. chartarum*, including biotechnological and industrial applications, as well as nanoparticles’ preparation, have been reviewed. The reported studies from 1973 to the beginning of 2022 are cited. For the reported metabolites, molecular weights and formulae, classes, places, hosts, and bioactivities were recorded, and their structures were also illustrated. Further, the pathways for biosynthesis of the main metabolites were discussed. The aim of this work is to draw the attention of chemists, biologists, and phytochemistry and fungi-interested researchers to these interesting metabolite producing fungus. Additionally, it highlights the possible utilization of these metabolites as drug leads that could change the research direction regarding this fungus. The data for this work were gathered via computer searches in various databases (Web of Knowledge, PubMed, and SCOPUS), scientific websites (Science Direct and Google Scholar), and publishers (Wiley Online Library, Taylor & Francis, JACS, Springer, Bentham, and Thieme).

## 2. *S. chartarum* Enzymes and Their Possible Applications 

Fungi possess diverse digestive-enzyme batteries. They can utilize agro-industrial wastes by yielding diverse enzyme types, such as xylanases, cellulases, amylases, laccases, and pectinases. These enzymes play a substantial ecological role in lignin-cellulosic materials’ decomposition and can be utilized in various biotechnological applications. In this work, the reported research on *S. chartarum* enzymes and their possible biotechnological and industrial applications have been discussed.

Polythene wastes are adversely affecting the environment due to strong reluctance towards degradation [33]. Biodegradation is one of the favorable solutions for conquering this problem [34]. *S. chartarum* was found to possess degradation potential for biodegradable and low-density polythene [34]. 

Andersen et al. also reported that the indoor strain of *S. chartarum* had no or little xylanolytic and cellulolytic potentials in the AZCL (Azurine-cross-linked) assay [35]. On the contrary, it exhibited amylolytic and cellulolytic potential [36,37] and had a lignocellulose complex-degrading enzymes system [28].

Kordula et al. purified and characterized stachyrase A (chymotrypsin-like serine protease) from *S. chartarum* that had wide substrate specificity and hydrolyzed various physiologically potential proteins, protease inhibitors, and collagen in the lung [38]. 

### 2.1. Laccases

Enzymes’ oxidizing phenols have diversified applications in various industries, including paper and wood pulp delignification, textile (dye and stain bleaching), and biosensors. Laccases are phenol oxidases that can oxidize several aromatic compounds [4,39,40]. Fungal laccases have a crucial role in developing the fruiting body, as well as in lignin degradation [41]. Some laccases are produced upon exposure to phenolic substances. 

Mander et al. purified a laccase enzyme from *S. chartarum* and its gene was separated and expressed in *Trichoderma reesei*, *Aspergillus niger*, and *A. nidulans*. This enzyme oxidized the artificial substrate ABTS (2,2-azino-di-(3-ethylbenzthiazolinsulfonate) [42]. Further, Janssen et al. stated that the insertion of the peptide sequences IERSAPATAPPP, YGYLPSR, SLLNATK, KASAPAL, and CKASAPALC inserted in the C-terminal of *S. chartarum* laccase by recombinant DNA tools resulted in laccase- peptide fusions that selectively targeted carotenoid stains and displayed enhanced bleaching potential on stained fabrics [43]. This suggested that the modification of certain enzymes could improve their activity, suggesting a new area of research in this fungus.

### 2.2. Mannanases

Mannans are constituents of hemicellulose that are found in plants, some algae, microorganisms, and tremella [44]. They include gluco-, galacto-, and galactogluco-mannanase well as linear mannan [45]. They have diverse applications, for example KG (konjac glucomannan) and LBG (locust bean gum, galactomannan) are commonly utilized for chronic diseases and obesity prevention and viscosity-boosting food additives, respectively [46,47]. Mannans’ degradation is accomplished by various GHs (glycoside hydrolases) such as β-mannanase, β-mannosidase, β-glucosidase, acetyl-mannan-esterase, and α-galactosidase producing manno-oligosaccharides [48]. The later were utilized as prebiotics that enhance immune responses and modulate gut microbiota [49]. Yang et al. identified two β-mannanase genes (*s331* and *s16942*) from *S. chartarum* that were expressed in *Aspergillus niger* with high protein titers and activities [50].

### 2.3. β-Glucanases

*β*-Glucans comprise glucose polymers heterogeneous group, including lichenan (β-1,4-1,3-glucan), cellulose (β-1,4-glucan), β-1,3(4)-glucan, and laminarin (β-1,3-glucan). Their hydrolysis is catalyzed by various kinds of *β*-glucanases, such as lichenases, cellulases, laminarinases, and *β*-1,3(4)-glucanases [51]. Lichenases are produced by various microorganisms, including fungi and bacteria, and have remarkable applications in detergent, food, feed, brewing, and wine industries as well as biodiesel and bioethanol production [51]. The expression of the gene *Cel12A* isolated from rotting cellulose rag-associated-*S. chartarum* by Picart et al. was triggered by rice straw than 0.1% glucose or 1% lactose [52,53]. The resulting enzyme Cel12A (GH12 family) had a lichenase potential. It also exhibited high potential towards barley mixed glucans and lichenan and low effectiveness on cellulose. Hence, Cel12A could have potential applications in various industries [53]. 

r-ScEG12, a recombinant glucanase gene from *S. chartarum,* belonging to GH12 (glycosyl hydrolase family12), was purified and expressed in *Pichia pastoris* [54]. It was found that Mn^2+^ and Cu^2+^ (%inhibition 50.97 and 71.64%, respectively) prohibited its activity, while Ca^2+^ and Na^+^ enhanced the activity. This proved the capacity of *S. chartarum* to secrete endoglucanases that could be beneficial for industrial use [54]. 

Xylanase is the principal enzyme accountable for hemicellulose hydrolysis. The *xya6205a* gene obtained from *S. chartarum* was expressed in *A. niger*. The obtained xylanase had optimum potential at 5.8 pH and 50℃ temperature and maintained 83% activity after 18 h in the alkaline buffer [55].

### 2.4. Fucoidanases and Alginate Lyases

Alginate and fucoidan are polysaccharides found in brown seaweed that have wide potential applications because of their diversified bioactivities. Alginate lyases are polysaccharide lyases that hydrolyze alginate by cleaving the glycosidic bond to produce oligo-alginates, which have a substantial role in feed, food, nutraceutical, biofuel, and pharmaceutical industries [56]. In addition, they possess film-formation, emulsifying, gelling, and plant-growth promoting capacities [57]. Fucoidan showed antioxidant, anticoagulant, antiviral, anticancer, anti-inflammatory, and immunomodulatory capacities [56,58]. In spite of these beneficial bioactivities, fucoidan molecules possess structural variation, high molecular weight, and viscous nature that limit their therapeutic and pharmaceutical applications, however, low molecular-weight fucoidan-derived oligosaccharides have a wide potential for applications [59]. Fucoidanases are accountable for fucoidan hydrolysis to fucooligosaccharides and are a substantial tool for fucoidan structural characterization [59]. It is noteworthy that *S. chartarum* was found to have fucoidanase and alginate lyases producing potential [60]

## 3. Secondary Metabolites from *S. chartarum*

Reported data displayed that *S. chartarum* is rich with various types of metabolites with diverse structural characteristics, including phenylspirodrimanes, trichothecenes, isoindoline derivatives, atranones, dolabellane diterpenoids, xanthones, chromenes, cochlioquinones, and cyclosporins. Here, they are classified according to their chemical classes. Furthermore, it was detected that some reported compounds having the same molecular formulae and chemical structures were given different nomenclature (e.g., stachartin A/stachybotrysin (**13**), arthproliferin E/stachybotrin E (**31**), stachybotrysin/stachybotramide (**32**)). Besides, some metabolites with different structures had the same names. Some formerly reported metabolites were also separated recently as new ones, e.g., atranone Q (**180**) and stachatranone C (**187**), reported in 2019 by Yang et al. [61] and isolated as new metabolites in 2020 by Qin et al. [62], which may be due to improper literature search. Herein, the separated metabolites from *S. chartarum* and their bioactivities, as well as their biosynthesis and structure/activity relation, have been highlighted.

### 3.1. Phenylspirodrimanes 

Phenylspirodrimanes are an uncommon class of meroterpenoids (terpenylphenol) reported from *Stachybotrys* genus. Structurally, they are featured by the fusion of spirocyclic drimane with a phenyl moiety via spirofuran ring. They are created from farnesyl-diphosphate and orsellinic acid via ilicicolin B intermediate [63]. Their dimers are derivatives with various structural scaffolds that are produced from two monomers’ dimerization through C-C or C-N linkage with or without an alkyl chain. These metabolites are designated as the most dominant and characteristic kind of mycotoxins in this genus [64]. Various phenylspirodrimanes have been purified and characterized from *S. chartarum* utilizing diverse chromatographic and spectral tools (Table 1). Their bioactivities were assessed using different bioassays that were discussed in this work.

Various illnesses are featured by unregulated and persistent angiogenesis [77,114]. The agents that inhibit angiogenesis have a substantial role in various diseases such as metastasis, cancer, hemangioma, arthritis, and ocular diseases [114]. Angiogenesis is a complicated multicellular process in which specified receptors and their ligands have a considerable role. It was reported that Tie2 receptor (tyrosine kinase receptor) and its ligands have an important function in angiogenesis [77,115].

**Scheme 1:**
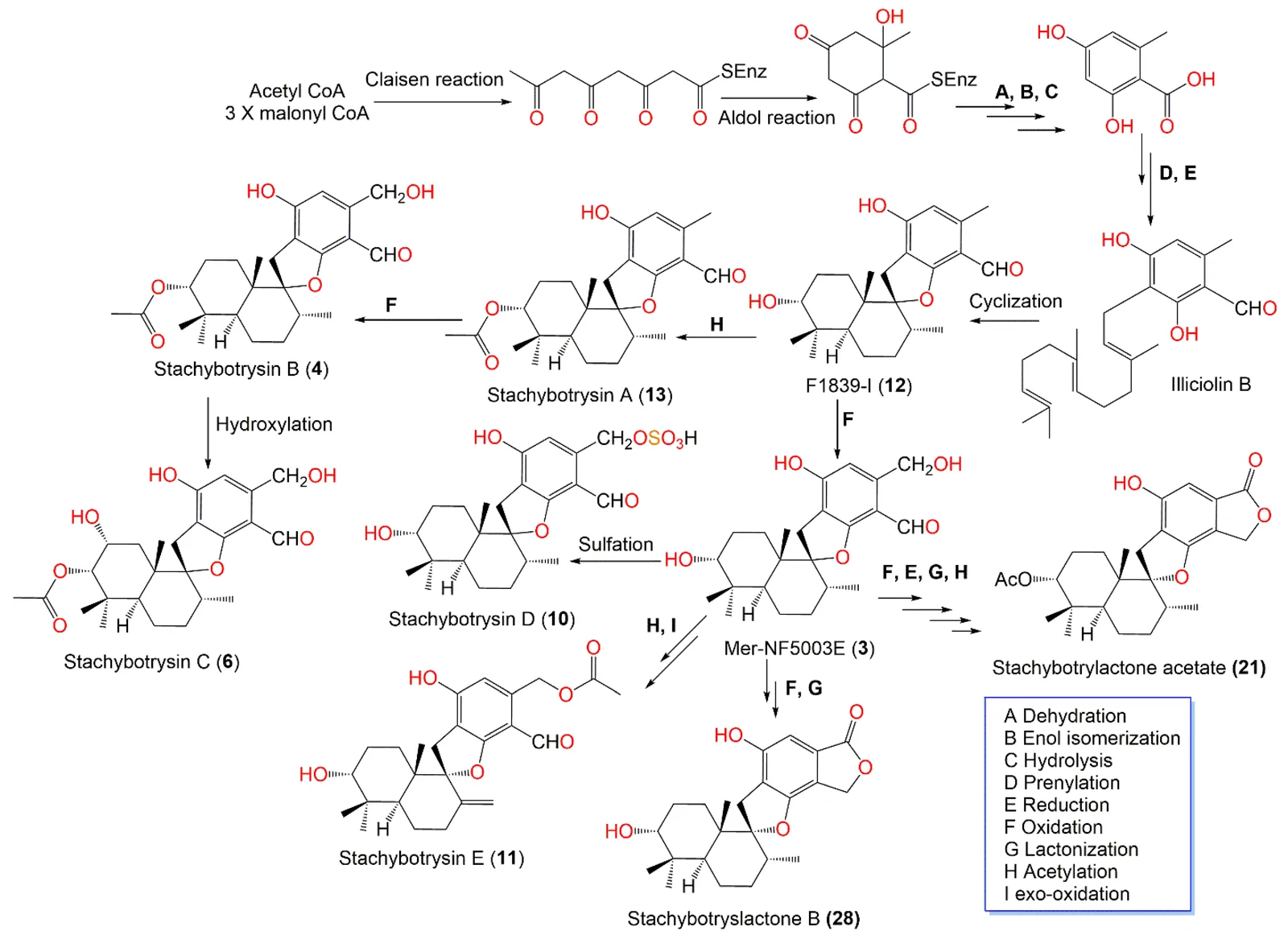
Biosynthetic pathways of compounds **3**, **4**, **6**, **10**–**13**, **21**, and **28** from ilicicolin B [67].

Compounds **4**, **6**, **10**, **11**, **13**, **16**, and **17** are new phenylspirodrimane derivatives that were isolated by Zhao et al. along with **3**, **12**, **14**, **21**, and **28** from *S. chartarum* CGMCC-3.5365 by using SiO_2_, RP, and HPLC. Their structures and configurations were established by spectral analyses, as well as X-ray, ECD (electronic circular dichroism), calculated ECD, and optical rotation. Stachybotrysin D (**10**) is an alcoholic O-sulfating derivative, whereas stachybotrysins F and G (**16** and **17**) have isobenzo-tetrahydrofuran moiety with a C-8’-attached acetonyl group. Their antiviral potential versus HIV-1 virus and influenza A virus (IAV) was estimated. Compounds **4**, **11**, **13**, and **17** displayed weak anti-HIV capacity (IC_50_ range 18.1–35.7 μM) compared to efavirenz (IC_50_ 4.0 nM), however **11, 13**, **14**, **16**, **17**, and **21** possessed inhibitory effectiveness versus IAV (IC_50_ range 12.4–45.6 μM) relative to ribavirion (IC_50_ 2.0 nM). In the cytotoxicity MTT assay, **12**, **13**, and **17** revealed weak potential versus HepG2 cell (IC_50_ 18.4, 24.7, and 24.6 μM, respectively) in comparison to paclitaxel (IC_50_ 6.3 nM), besides, **12** was weakly active versus BGC823 and NCI-H460 cell lines (IC_50_s 21.9 and 15.8 μM, respectively) [67]. These metabolites are terpenoid-polyketide hybrids that were proposed to be originated from ilicicolin B, which was established to be biosynthesized from farnesyldiphosphate and orsellinic acid (Scheme 1). 

**Scheme 2:**
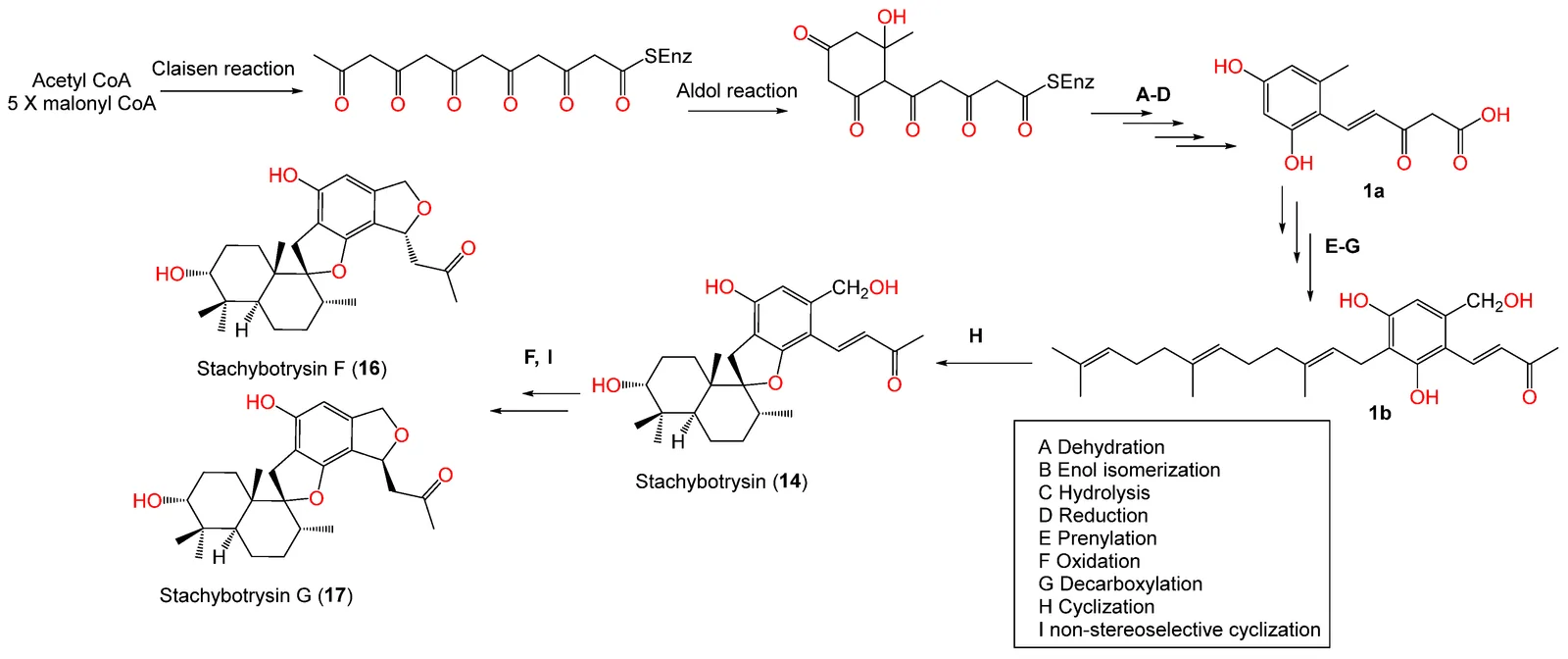
Biosynthetic pathway of **14**, **16**, and **17** [67].

On the other side, **14**, **16**, and **17** featured an additional C3 chain bound to the phenolic unit, revealing their origin from a non-orsellinic acid precursor (Scheme 2). It was suggested that they had a polyketone precursor (**1a**) derived from an acetyl-CoA (starter) and 5 malonyl-CoA (extenders). The polyketone farnesylation, oxidation, and decarboxylation yield **1b**. The latter undergoes several enzymatic cyclization to afford **14** and subsequent oxidation and non-stereoselective cyclization to form **16** and **17** [67].

Further, new phenylspirodrimanes; stachybotrysins H and I (**18** and **19**) and stachybotrin E (**31**) along with stachybotrylactam (**29**) were separated from *S*. *chartarum* CGMCC-3.5365 mycelia and filtrate EtOAc extract by SiO_2_ CC and HPLC that were established by NMR and ECD analyses, as well as optical rotation [71]. They have 2R/3S/5S/8R/9R/10S/8’R, 2R/3S/5S/8R/9R/10S/8’S, and 3R/5S/8R/9R/10S configurations, respectively. Kv1.3 (voltage-gated K channel) controls both non-excitable and excitable cell membrane potential and has a remarkable contribution in Ca^2+^ signaling regulation [116]. It has been signaled as a pronounced target for remedial intervention, particularly for multiple sclerosis, type 1 diabetes, psoriasis, and autoimmune disorders [116]. Compounds **18** and **19** possessed Kv1.3 inhibitory effectiveness (IC_50_ 13.4 and 10.9 μM, respectively) compared to clofazamine (IC_50_ 2.01 μM) and PAP-1 (5-(4-phenoxybutoxy)psoralen, IC_50_ 0.17 μM) in the Kv1.3 FLIPR (fluorometric imaging plate reader) thallium flux assay using CHO-Kv1.3 stable cell line [71].

**Figure 1:**
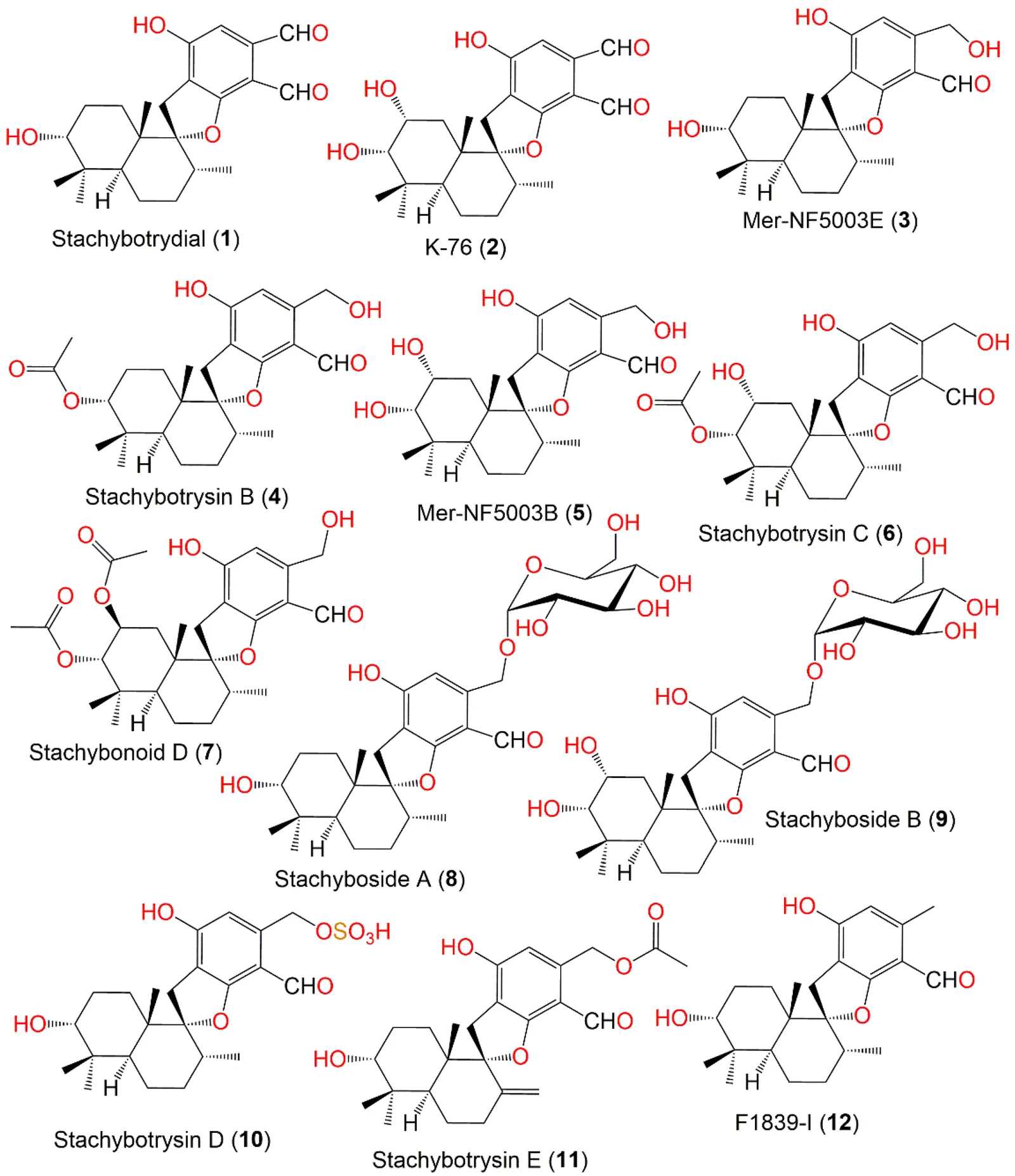
Structures of phenylspirodrimane derivatives (**1**–**12**) reported from *S*. *chartarum*.

Seven novel K-76 (**2**) derivatives, K-76-1 (**36**), K-76-2 (**37**), K-76-3 (**59**), K-76-4 (**60**), K-76-5 (**61**), K-76-6 (**62**), and k-76-7 (**63**), were purified from ethyl-methyl-ketone extract by flash SiO_2_ CC and HPLC and elucidated by NMR and MS spectroscopic tools (Figure 1). They are drimane sesquiterpenoids having phenylspirodrimane moiety linked with an α,β-unsaturated γ-lactam ring. They differed in the hydroxylation at C-2 and the nature of the N-linked substituent of the γ-lactam ring [77]. These metabolites were assessed for their potency to prohibit the ATP ^33^P incorporation into the Tie2 intracellular tyrosine kinase portion in the auto-phosphorylation reaction. All metabolites notably prohibited Tie2 kinase receptor (IC_50_s ranging from 0.025 to 0.146 mM), where **60** was the most powerful one (IC_50_ 0.025 mM) [77].

**Figure 2:**
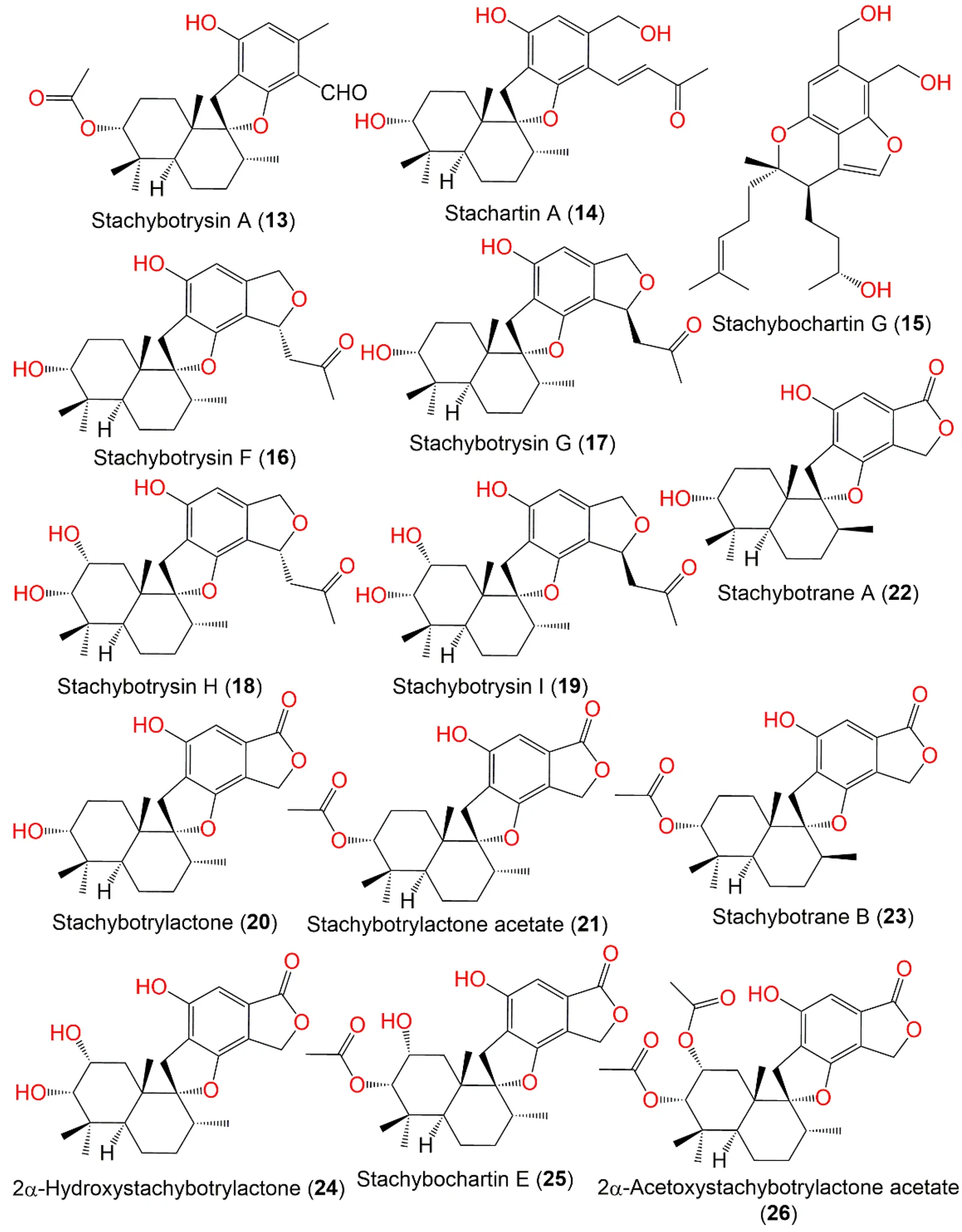
Structures of phenylspirodrimane derivatives (**13**–**26**) reported from *S*. *chartarum*.

From the wetland strain, new phenylspirodrimanes, stachybotranes A–D (**22**, **23**, **27**, and **58**) and formerly reported **20**, **21**, **24**, **25**, and **32,** were purified by RP-18 and Sephadex CC and HPLC and assigned via spectroscopic, X-ray, and CD analyses. Compounds **22**/**23** and **27**/**58** had 3R,5S,8S,9R,10S and 2R,3S,5S,8S,9R,10S configurations, respectively (Figure 2). Compounds **21, 22**, and **23** were moderately cytotoxic (IC_50_ ranging from 9.23 to 31.22 μM) versus A-549, SMMC-7721, MCF-7, SW-480, and HL-60 in the MTT assay compared to cisplatin (IC_50_ 1.25–17.63 μM). The structure/activity relation demonstrated that the lactone groups had a positive influence on cytotoxicity compared with the lactam ring [72].

**Scheme 3:**
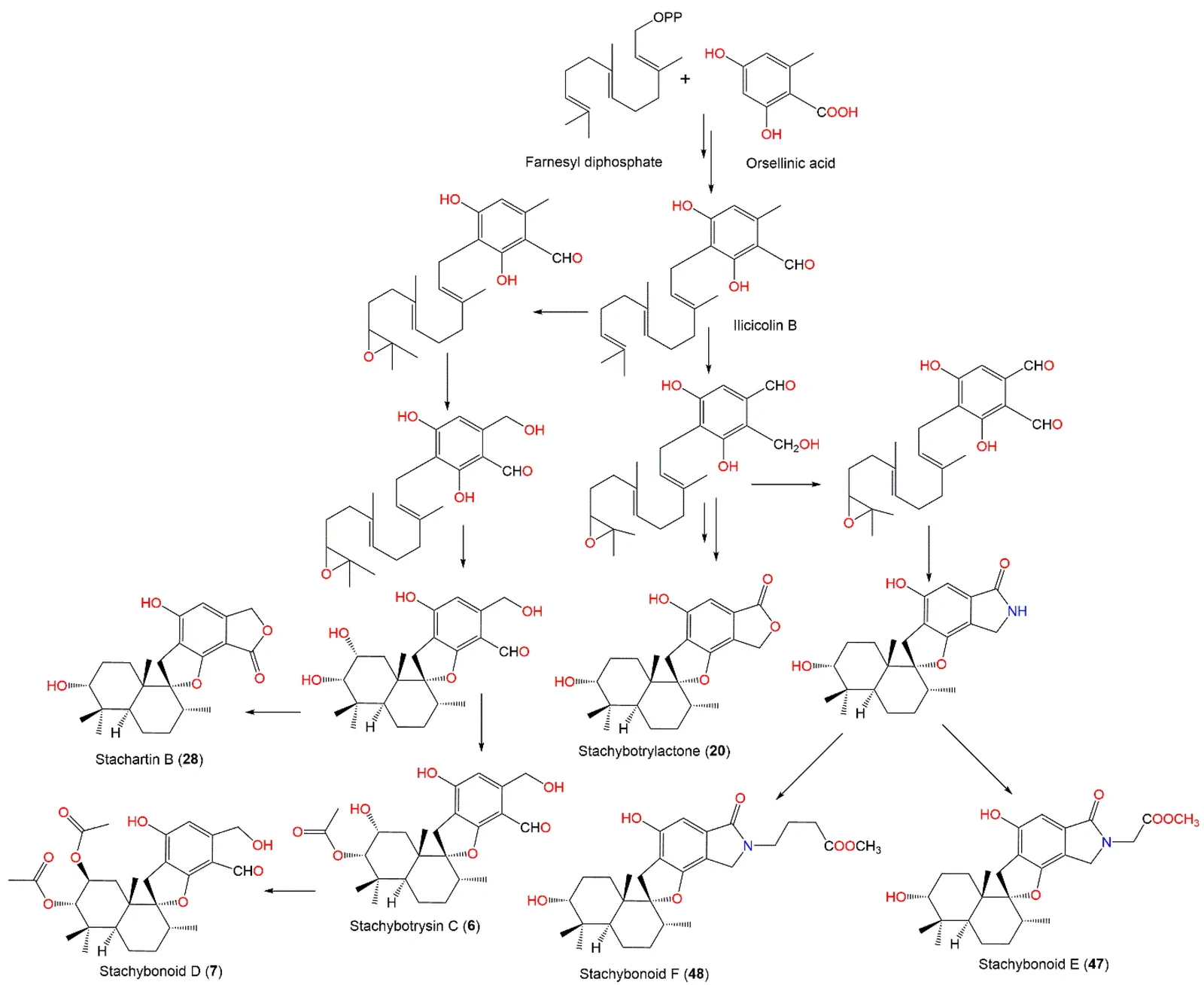
Biosynthetic pathway of **6**, **7**, **20**, **28**, **47**, and **48** [68].

The new phenylspirodrimanes; stachybonoids D–F (**7**, **47**, and **48**), in addition to stachybotrysin C (**6**), stachybotrylactone (**20**), and stachartin B (**28**), were separated using HPLC and SiO_2_ and Sephadex LH-20 CC and characterized based on spectral, X-ray, and ECD. These metabolites shared the same 2S,3S,8S,10S-configurations. Their inhibition capacity versus LPS-boosted NO production in RAW264.7 cells was estimated in the Griess assay. It was found that **6**, **20**, and **48** displayed moderate inhibition of NO production (IC_50_ 27.2, 17.9, and 52.5 μM, respectively) compared to indomethacin (IC_50_ 37.5 μM), while **7**, **47**, and **28** were inactive [68]. Zhang et al. assumed that these metabolites are generated from the addition reaction of farnesyl diphosphate and orsellinic acid to produce an intermediate ilicicolin B (Scheme 3). After that, the epoxidation of the prenyl group terminal olefinic bond takes place and the aromatic OH group reacts with C2 of the prenyl group. This is followed by a series of reactions, including cyclization, addition, oxidation, and acetylation, to produce **6**, **7**, **20**, **28**, **47**, and **48** [68].

**Figure 3:**
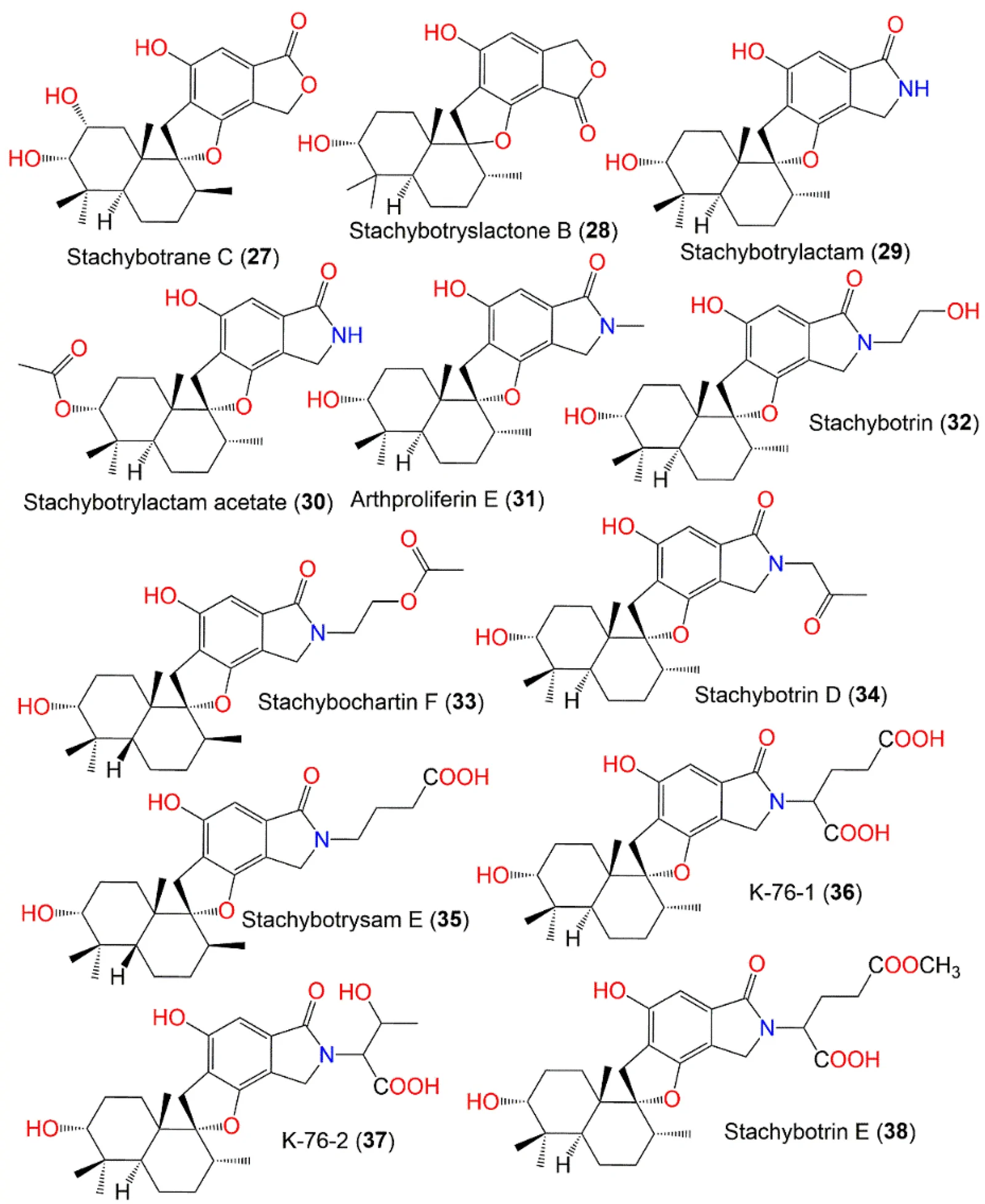
Structures of phenylspirodrimane derivatives (**27**–**38**) reported from *S*. *chartarum*.

From *Xestospongia testudinaris*-associated *S. chartarum,* the new derivatives, stachybosides A (**8**) and B (**9**), stachybotrins D-F (**34**, **38**, and **39**), and stachybocins E (**113**) and F (**114**), along with **3**, **5**, **12**, **36**, and **51,** were separated (Figure 3) and elucidated using spectroscopic tools and their configuration was estimated based on alpha D, modified Mosher‘s and Marfey‘s methods, and chemical hydrolysis. Compound **34** was like **36,** except the side chain on the N-atom was replaced by an acetonyl group and had 3R/5S/8R/9R/10S configuration, whilst **38** and **39** are methylated derivatives **36** having the same alpha D but differing in the position of the methyl group. Besides, **113** and **114** are dimers that were structurally like **34** except for the replacement of the acetonyl moiety at nitrogen with two and three methylenes, respectively. 

On the other side, **8** and **9** have glycosylated hydroxymethyl moiety instead of the hydroxymethyl group in **3**, whereas **9**, a 2-OH congener of **8**, has 2R/3S/5S/8R/9R/10S configuration. Compound **34** prohibited HIV-1 replication towards 5 NNRTI-resistant and wild-type HIV-1 strains by targeting reverse transcriptase (EC_50_ 6.2–23.8 μM) compared to nevirapine (EC_50_ 0.023–51.9 μM) in the luciferase assay system [66] (Table 2).

**Figure 4:**
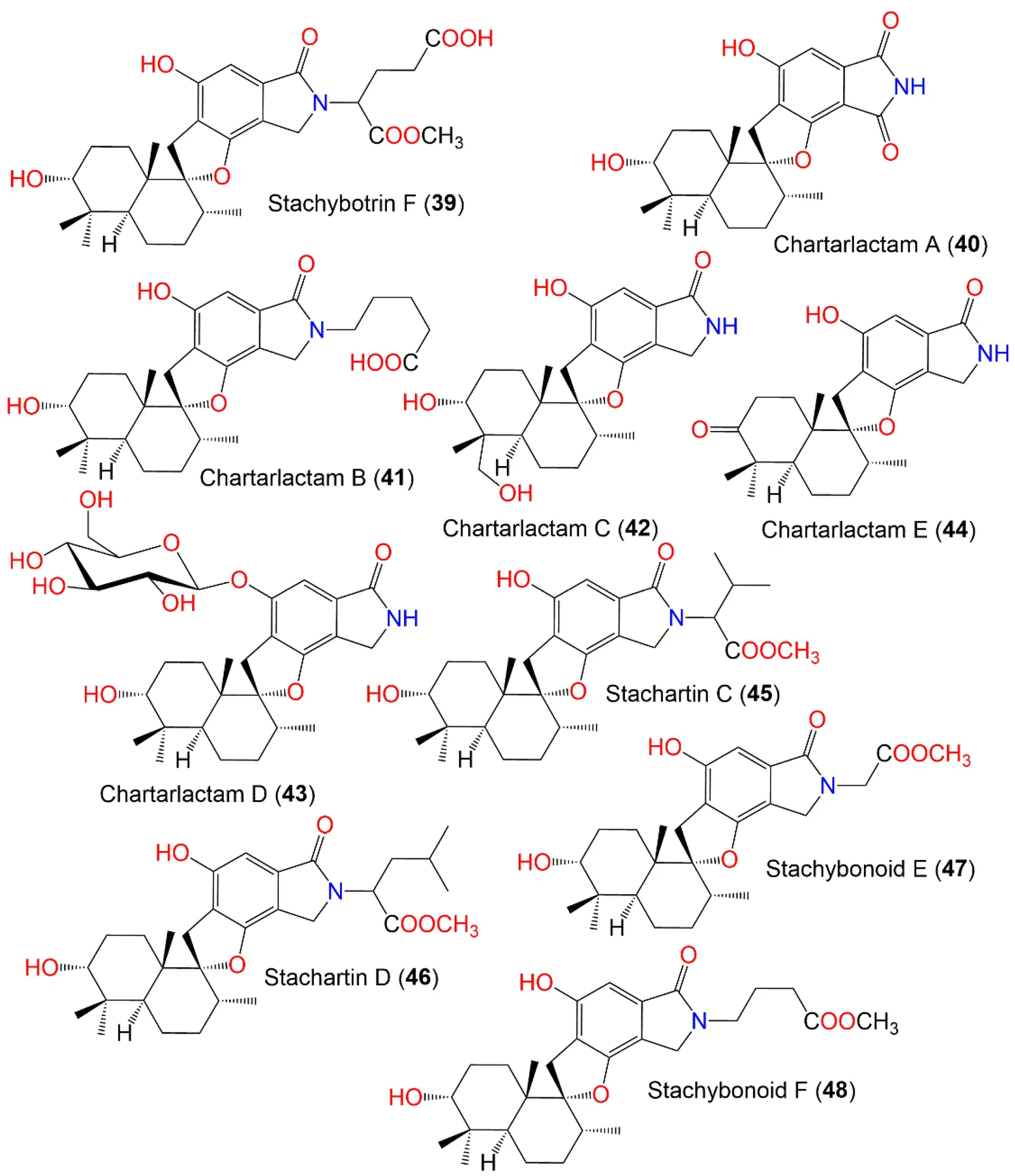
Structures of phenylspirodrimane derivatives (**39**–**48**) reported from *S*. *chartarum*.

Yang et al. (2021) purified and characterized undescribed phenylspirodrimane derivatives, stachybotrolide (**20**), stachybotrylactam acetate (**30**), arthproliferin E (**31**), and formerly reported **32**, **44**, **51**, **64**, and **65** from *Sinularia* sp.-associated *S. chartarum* SCSIO4-1201 EtOAc extract (Figure 4). These metabolites were evaluated for their antimicrobial activity versus *A. baumannii* ATCC-19606, *K. pneumonia* ATCC-13883, *E. coli* ATCC-25922, *A. hydrophila* ATCC-7966, *S. aureus* ATCC-29213, and *E. faecalis* ATCC-29212 in the serial dilution technique using 96-well microtiter plates and for cytotoxicity against MDA-MB-231, C4-2B, MGC803, MDA-MB-468, and A549 cell lines in the CCK-4 assay. Compounds **30** and **31** showed weak inhibitory activity (MICs 325 and 125 µg/mL, respectively) towards methicillin-resistant *S. aureus* ATCC-29213 compared to ampicillin (MIC 10.0 µg/mL) and weak cytotoxic activity versus MDA-MB-231, C4-2B, MGC803, MDA-MB-468, and A549 [73].

**Figure 5:**
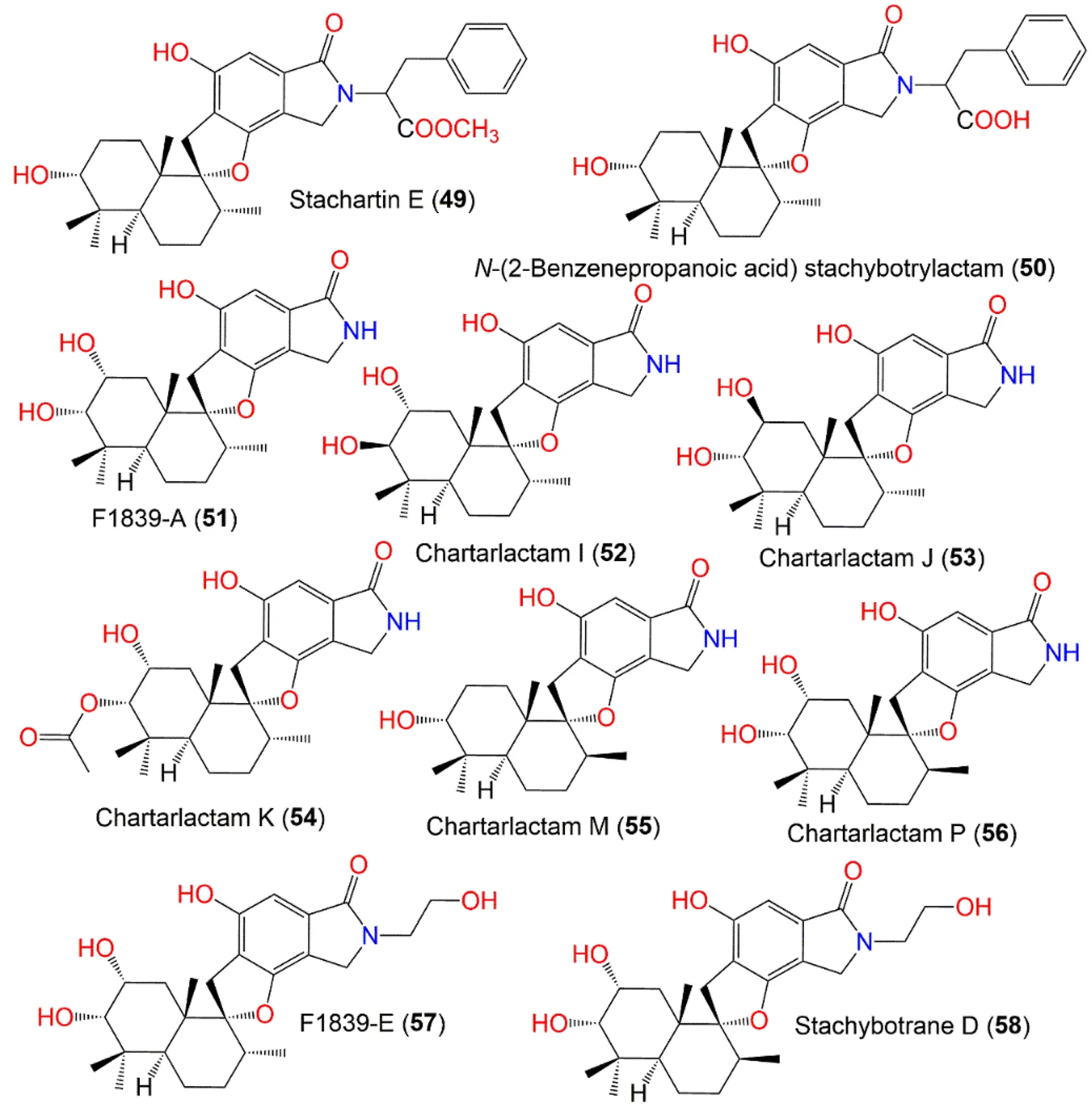
Structures of phenylspirodrimane derivatives (**49**–**58**) reported from *S*. *chartarum*.

Chemical investigation of the solid culture of the *S. chartarum* isolated from *Niphates recondita* using SiO_2_ and ODS gel CC and HPLC resulted in the separation of sixteen new phenylspirodrimanes, chartarlactams A–P (**40**–**44**, **52**–**56**, **65**–**69**, and **72**) along with **29**, **30**, **32**, **50**, **51**, **57**, **64**, and **71** (Figure 5). Their structures and configuration were verified using spectral tools and single-crystal X-ray diffraction, respectively. This represented the first report of 8β-CH_3_ analogs, and **72** had a new framework that was formed by **40** dimerization. 

**Figure 6:**
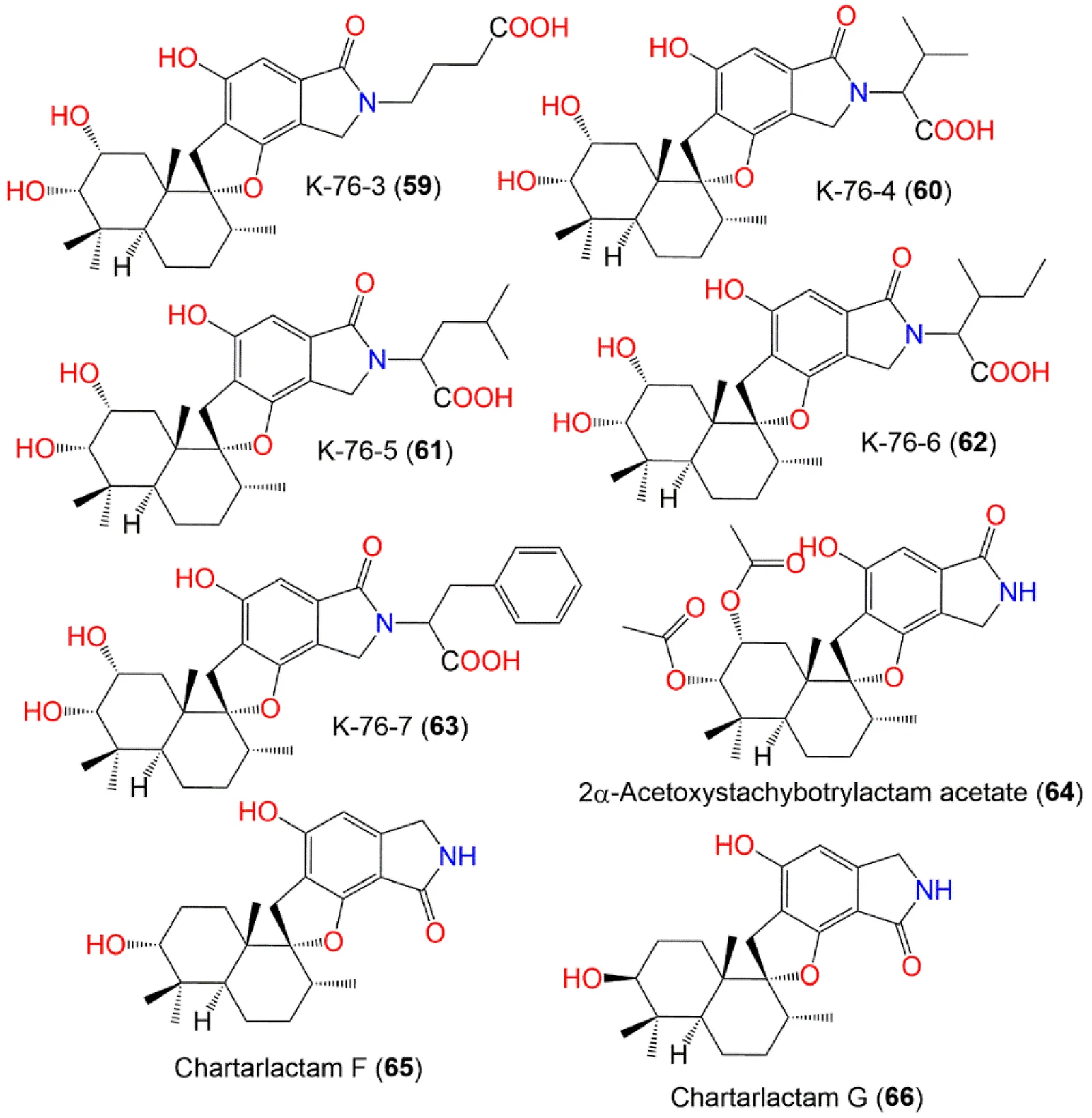
Structures of phenylspirodrimane derivatives (**59**–**66**) reported from *S*. *chartarum*.

The antihyperlipidemic effects of **29**, **30**, **40**–**44**, **50**–**57**, **65**, **67, 69**, **71**, and **72** (Figure 6) in HepG2 cells were estimated utilizing a cell-based lipid accumulation assay. The results revealed that **43**, **44**, **50**, **54**, **65**, **68**, **69**, and **72** (Conc. 10 μM) possessed significant lipid-lowering potential in HepG2 cells. Besides, **44**, **50**, **54**, **65**, and **69** displayed remarkable prohibition of intracellular TG (triglyceride) levels, whereas **43**, **44**, **50**, **65**, **68**, and **72** dramatically lessened TC (total cholesterol). The structure–activity relation revealed that the 8α-CH_3_ analogs with alkyl N-substituted (e.g., **30**, **32**, **41**, and **67)** had weak potential, except **50,** that has a benzene-propanoic acid substituent. In addition, the C-8‘ γ-lactam carbonyl as in **65** and **69** boosted the inhibitory potential compared with **29** and **55**, which have C-7‘carbonyl. Compound **43**, with a C-2‘glucosyl moiety, possessed only inhibition on TC, whereas **54** selectively prohibited TG. The analogs with 2,3-diol had no activity, whereas C-3 acetoxy analogs (e.g., **54**) had a selective inhibitory effect [74].

**Figure 7:**
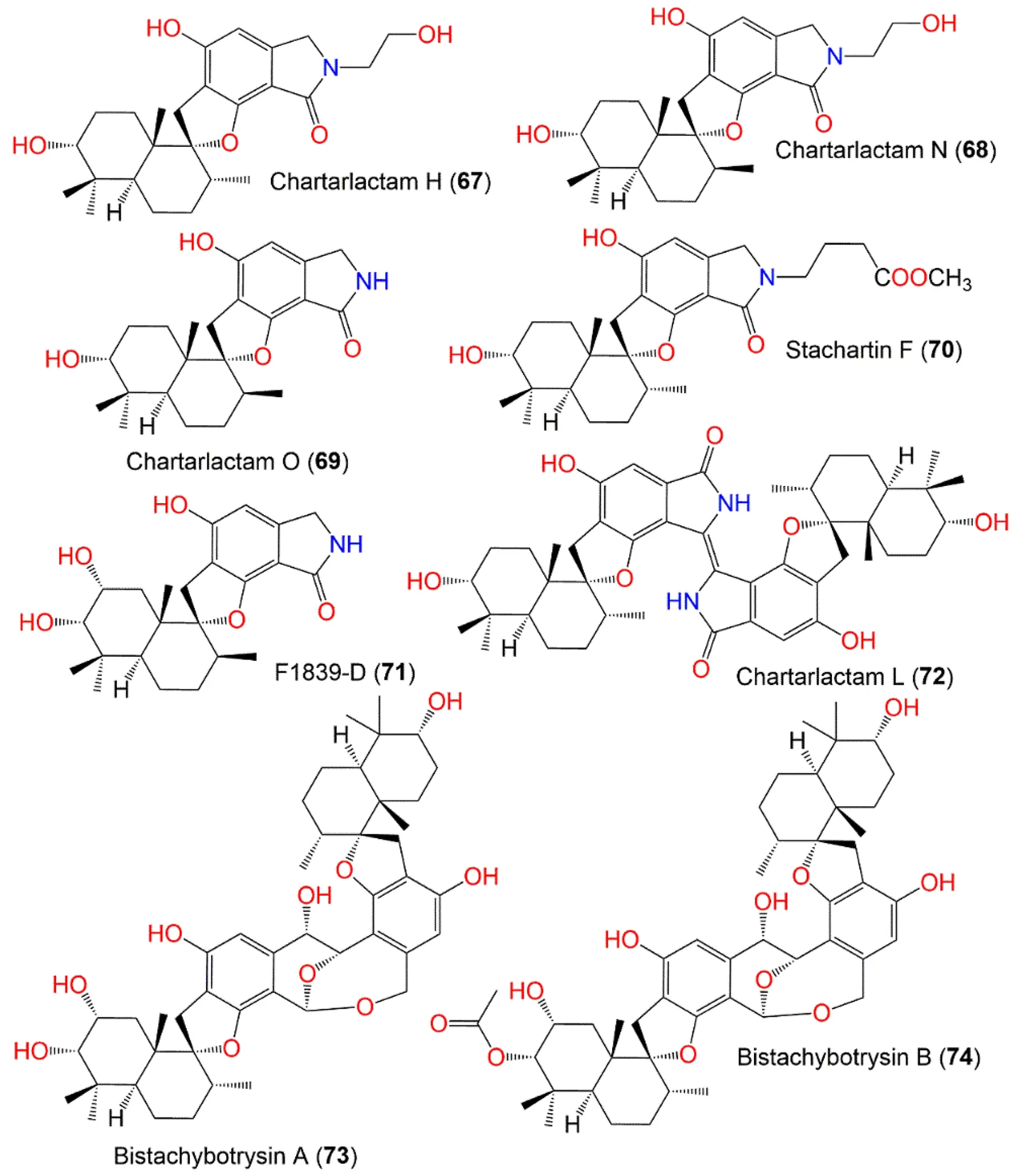
Structures of dimeric phenylspirodrimane derivatives (**67**–**74**) reported from *S*. *chartarum*.

Zhao et al. reported the separation of three dimers: bistachybotrysins A–C (**73**–**75**) from *S. chartarum* CGMCC-3.5365 mycelia EtOAc extract by SiO_2_ CC and HPLC that were assigned based on spectroscopic and ECD analyses. These metabolites are phenylspirodrimane dimers having [6,6,7,6]-tetracyclic scaffold with 6/7 oxygen heterocycle linkage and a central 2,10-dioxabicyclo[4.3.1]decan-7-ol core fused with two phenyl moieties (Figure 7). 

Their in vitro cytotoxic potential versus HCT-116 (colorectal carcinoma), NCI-H460 (lung carcinoma, BGC823 (gastric carcinoma), Daoy (medulloblastoma), and HepG2 (liver carcinoma) in the MTT assay was assessed. 

**Figure 8:**
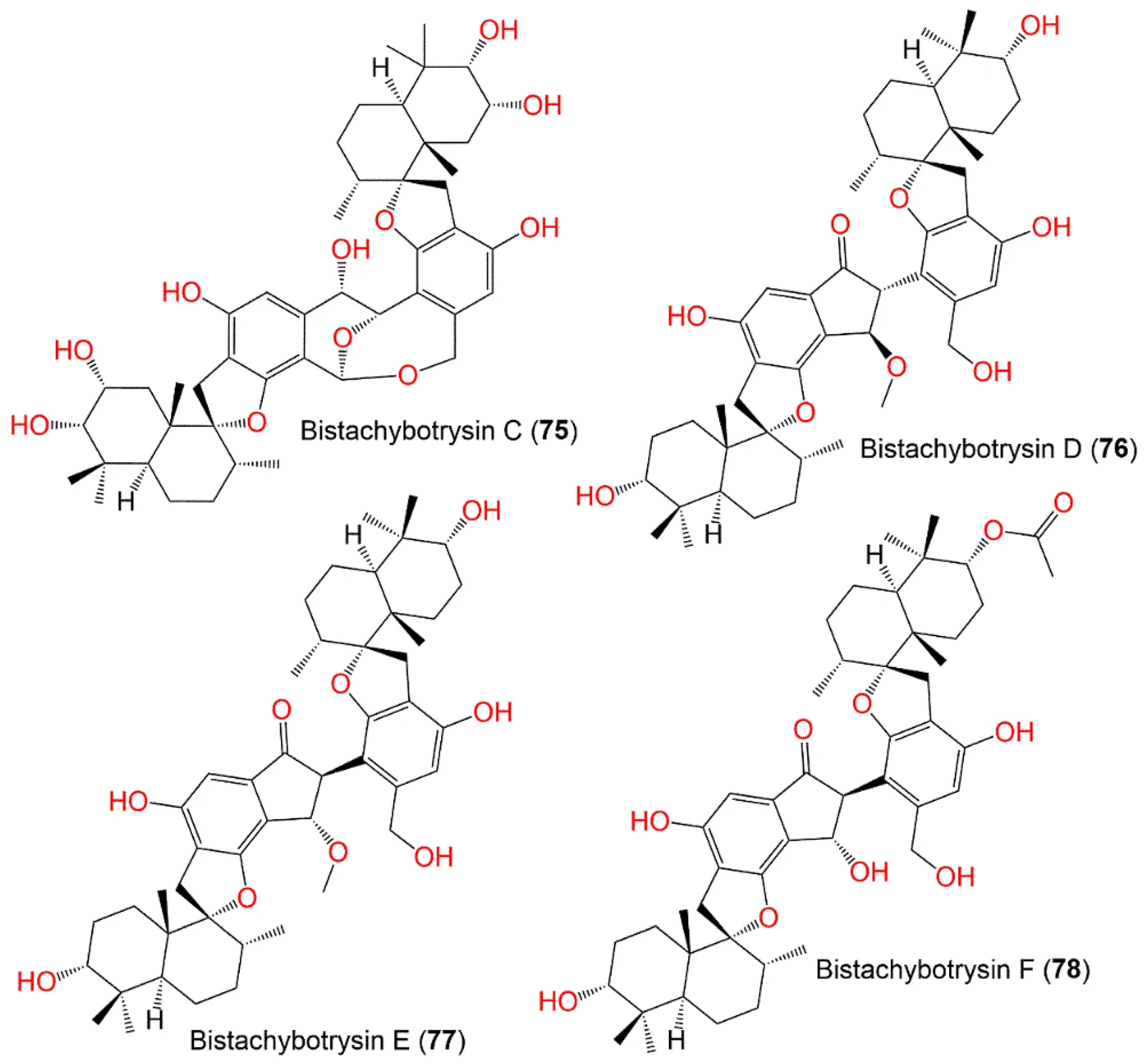
Structures of dimeric phenylspirodrimane derivatives (**75**–**78**) reported from *S*. *chartarum*.

Compound **73** revealed powerful inhibitory capacity versus Daoy, HCT-116, and NCI-H460 (IC_50_S 2.8, 6.7, and 7.5 μM, respectively), whereas **74** had potent activity versus Daoy, NCI-H460, and BGC823 (IC_50_S 4.2, 5.5, and 6.6 μM, respectively). On the other side, **75** demonstrated weak cytotoxic effectiveness versus all cell lines. It was observed that **73** was more potent versus Daoy and HCT-116 than **74**, while **74** possessed better influence versus BGC823 and NCI-H460 than **73**, indicating that different substituents at C-3 might influence the selectivity towards tumor cell lines and substituent at 2’-OH might lessen the activity (Figure 8). 

**Scheme 4:**
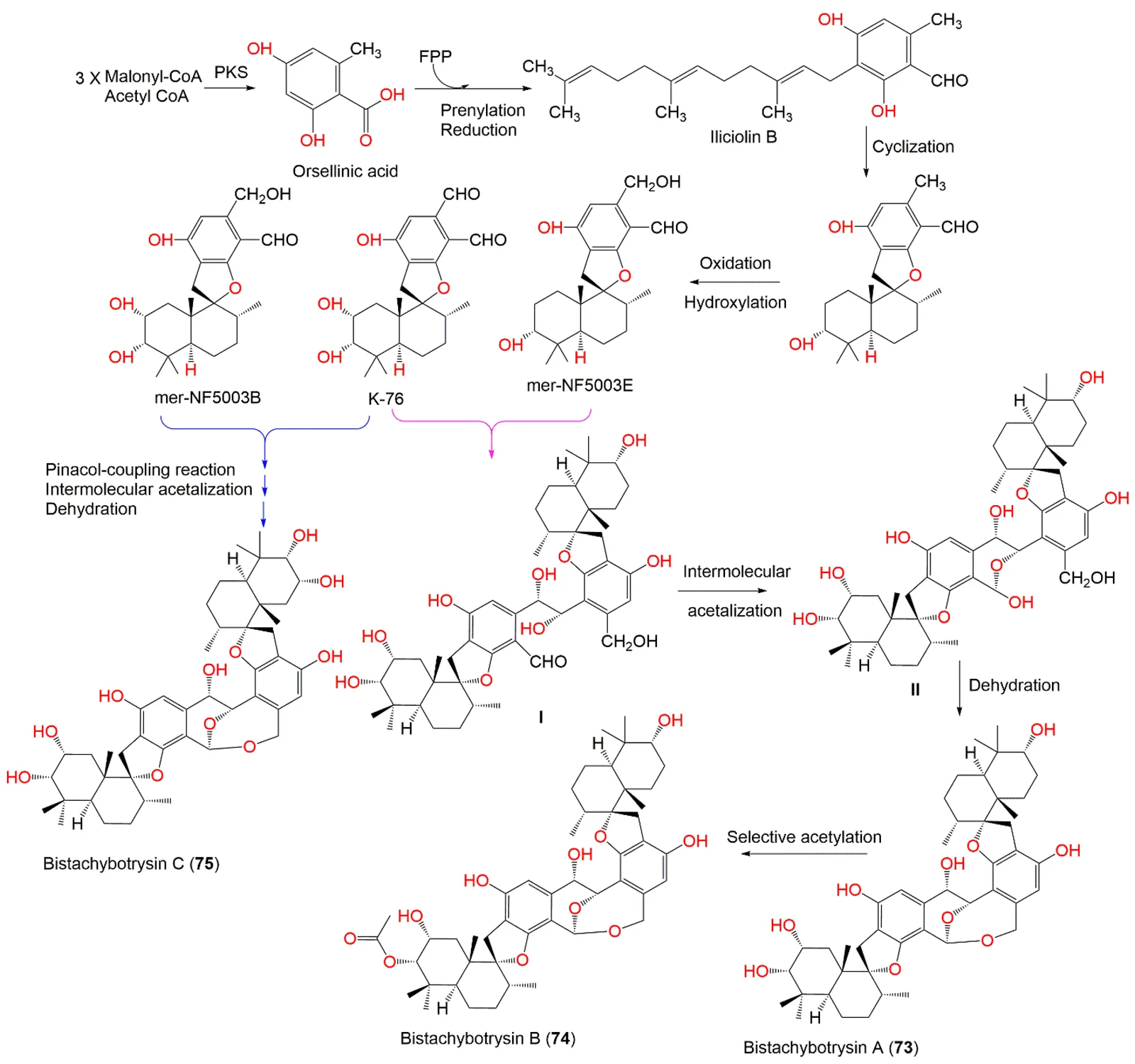
Biosynthetic pathway of bistachybotrysins A–C (**73**–**75**) [79].

Further investigation is worth pursuing for **73** and **74** as new promising antitumor lead compounds [79]. They were postulated to be originated from farnesyl-diphosphate and orsellinic acid that give phenylspirodrimane monomer (Scheme 4). Then, a pinacol coupling reaction among the two CHO groups of **3** and **2** gives a vicinal-diol intermediate **I**. After that, one OH group of the 22,23‘-diol moiety fuses further with the other CHO of **2** to produce **II** with a six-membered oxygen heterocyclic ring through acetalization intermolecularly. Subsequently, the dehydration of the mer-NF5003E (**3**) CH_2_OH and hemiacetal OH groups yields **73** through 6/7 oxygen heterocyclic linkage. Further, **74** is resulted from bistachybotrysin A by selective acetylation, whereas **75** is generated from **5** and **2** through pinacol-coupling reaction, intermolecular acetalization, and dehydration [79]. 

Bistachybotrysins D (**76**) and E (**77**), stereoisomeric phenylspirodrimane dimers, have a central [6,5,6]-tricyclic carbon skeleton involving a cyclopentanone ring. Their structures were verified by extensive spectral analysis, and their configurations were established by ECD. Compound **76** demonstrated [6,5,6]-tricyclic carbon scaffold that dimerized through 3-(hydroxymethyl)cyclopentanone core C-C connected to one phenyl ring and fused to the other. Compound **77** has the same structure as **76** with 23S/23’S instead of 23R/23′R in **76**. These metabolites (IC_50_ ranged from 6.7 to 11.6 μM) had pronounced cytotoxic potential versus Daoy, HCT116, BGC823, and HepG2 cell lines. Besides, **76** possessed neural anti-inflammatory potential by prohibiting NO production in BV2 cells induced by LPS compared to curcumin (inhibitory rate 61.1 and 67.6%, respectively, at Conc. 10 μM) [80]. 

**Scheme 5:**
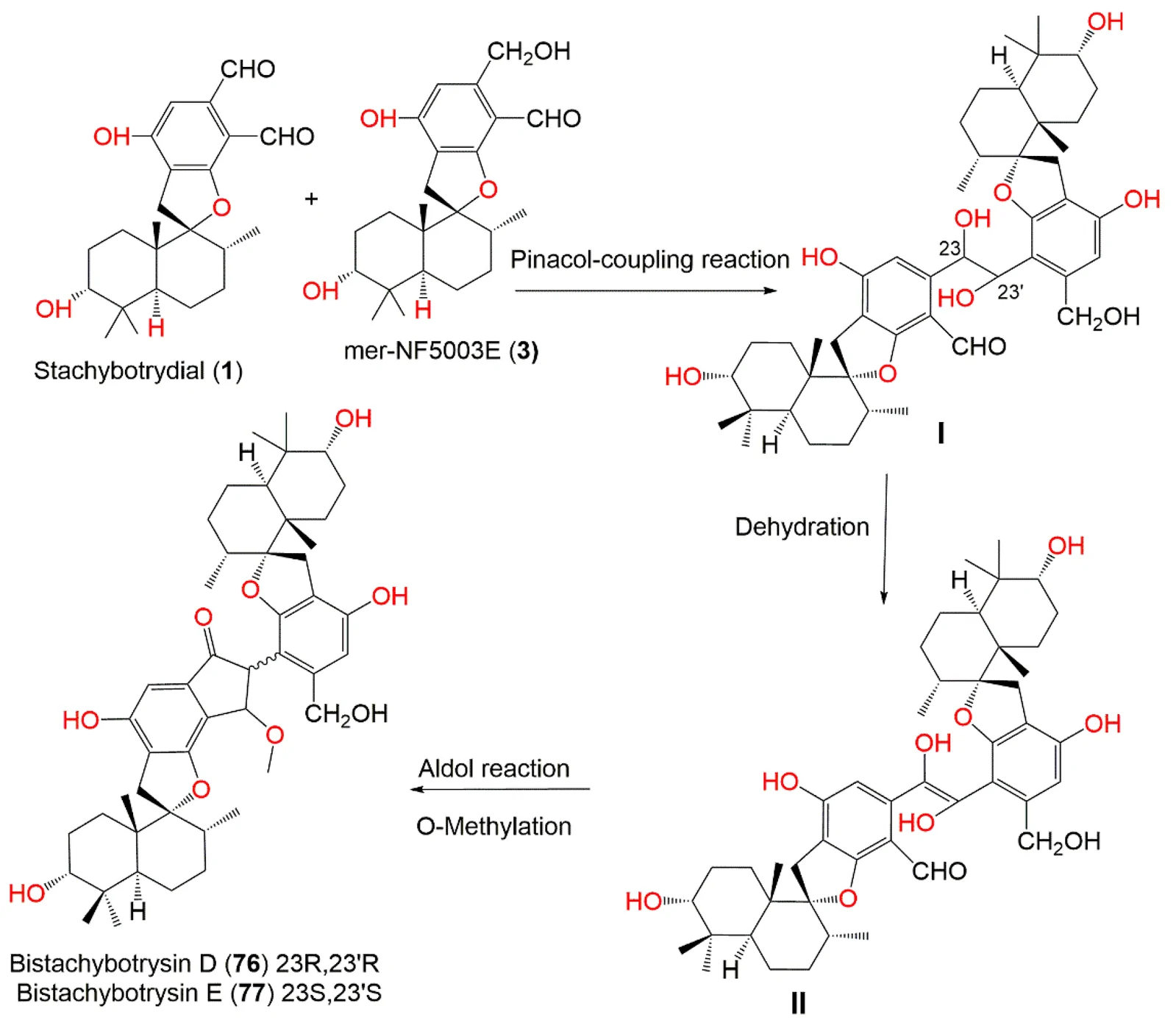
Biosynthetic pathway of bistachybotrysins D (**76**) and E (**77**) [80].

The monomers are biosynthesized from FPP (farnesyldiphosphate) and orsellinic acid by reduction, prenylation, and cyclization. Further, hydroxylation and oxidation form **3** and **1** that undergo a pinacol coupling reaction between the two aldehydic groups to produce **I** (vicinal diol intermediate) that is dehydrated by H-22 and OH-23′ to yield **II**. Subsequently, the non-stereoselective aldol reaction of **II** gives a stereoisomer pair, and further O-methylation affords **76** and **77** (Scheme 5) [80].

**Figure 9:**
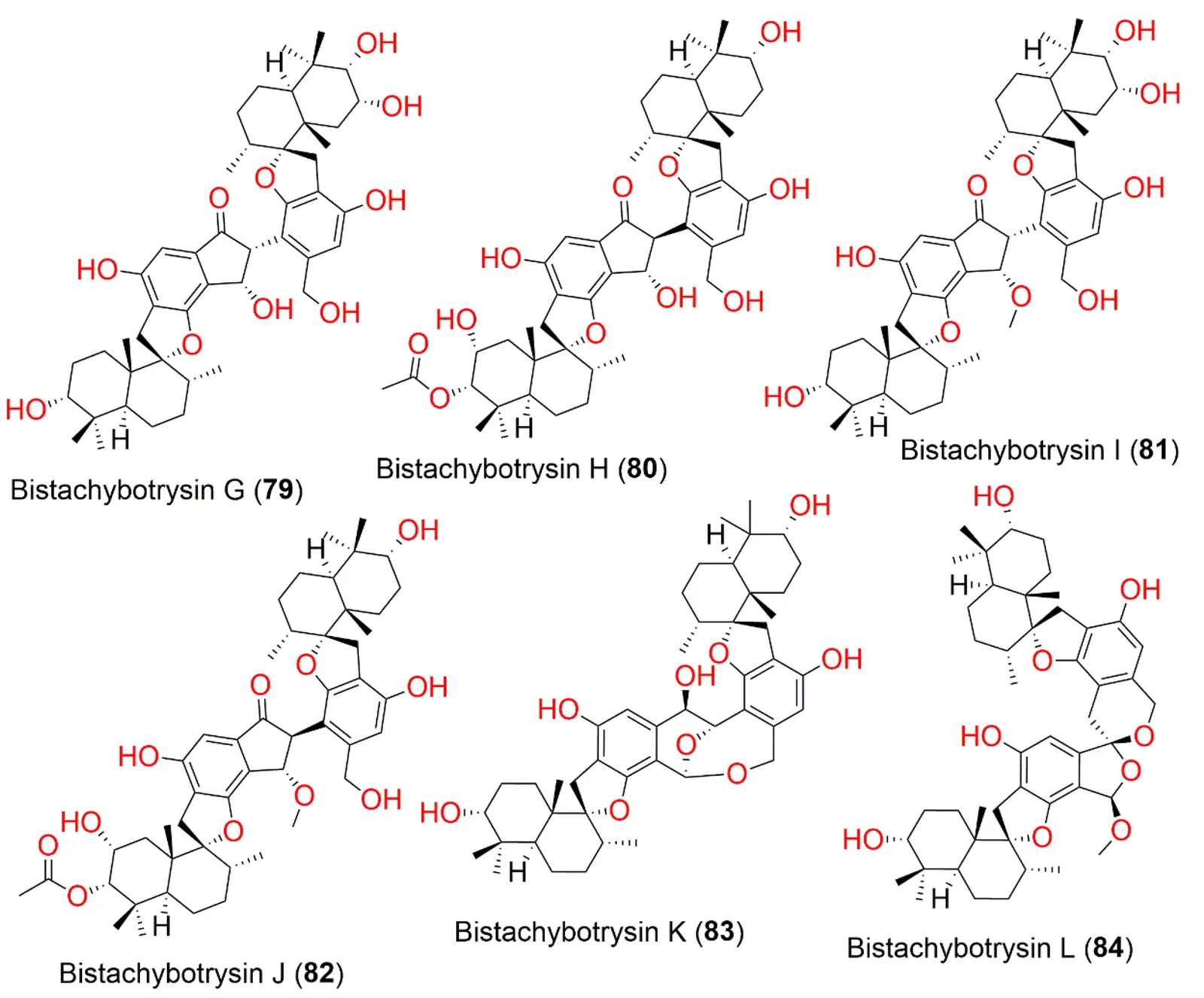
Structures of dimeric phenylspirodrimane derivatives (**79**–**84**) reported from *S*. *chartarum*.

Based on HPLC-UV/MS guided analyses, five new dimers, bistachybotrysins F–J (**78**–**82**), having cyclopentanone ring linkage were purified and characterized using SiO_2_CC/HPLC and NMR/HRMS/ECD, respectively [81]. Compound **78** displayed the same structural features as **77** and **111**. Besides, **78**, **80**, and **82** had 23R/23’R configuration, whereas **79** and **81** possessed 23S/23’S configuration (Figure 9). 

**Scheme 6:**
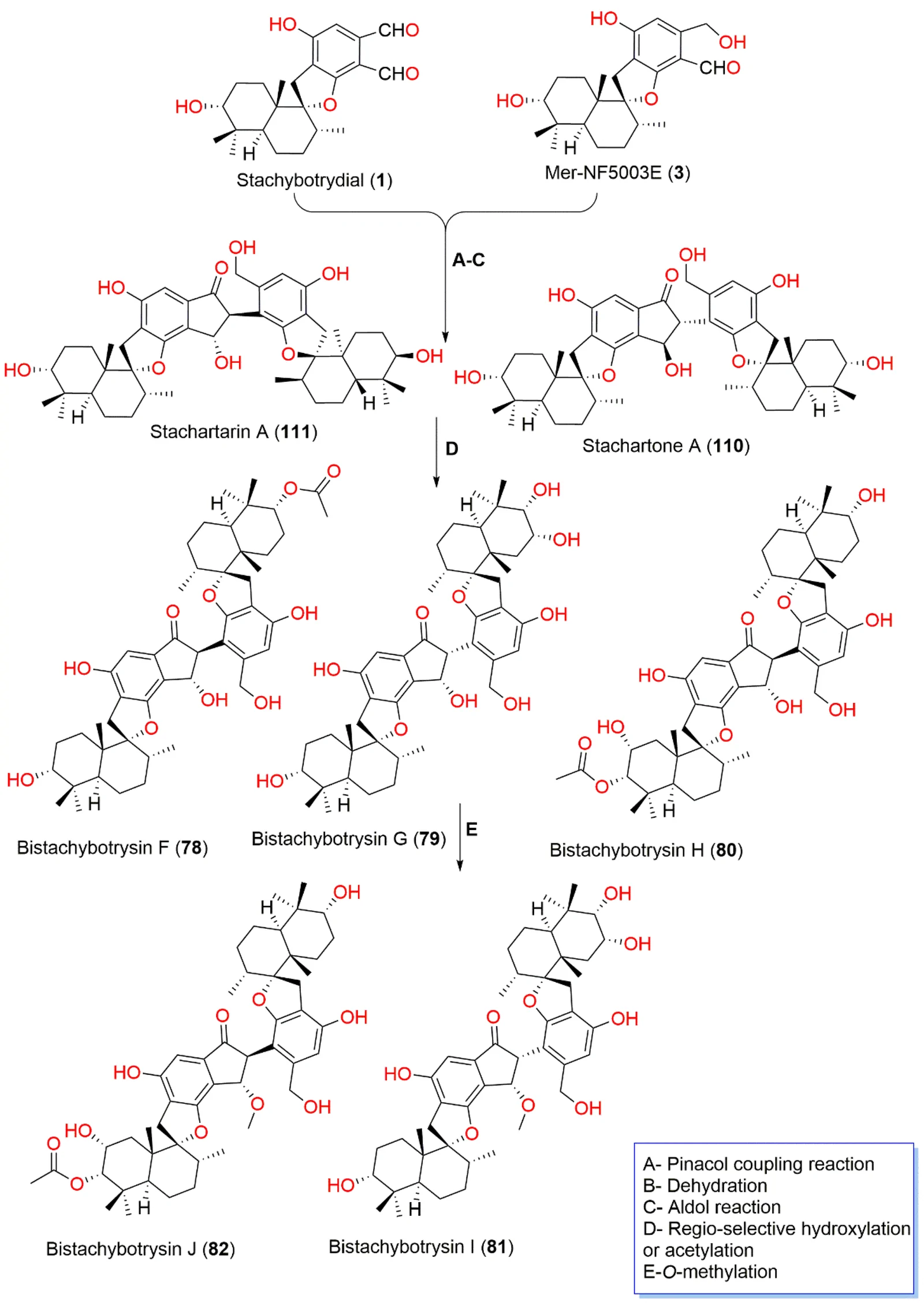
Biosynthetic pathway of bistachybotrysins F–J (**78**–**82**) [81].

Feng et al. postulated that bistachybotrysins F–J (**78**–**82**) are generated from **1** and **3**. They undergo a pinacol coupling reaction among the two CHO to yield a vicinal diol intermediate, subsequently, **110** and **111** are formed through dehydration and non-stereoselective aldol reaction. After that, the regioselective hydroxylation (C-2 or C-2’) affords **78**–**80** and/or acetylation (OH-3’ or OH-3). Further, **79** and **80** O-methylation produces **81** and **82**, respectively (Scheme 6) [81]. Additionally, these metabolites were evaluated for cytotoxic potential versus HCT116, NCI-H460, BGC823, Daoy, and HepG2. It was found that **78** and **80**–**82** were moderately active (IC_50_ 9.1–22.8 μM) versus HepG2, NCI-H460, BGC823, and HCT116 [81]. 

**Figure 10:**
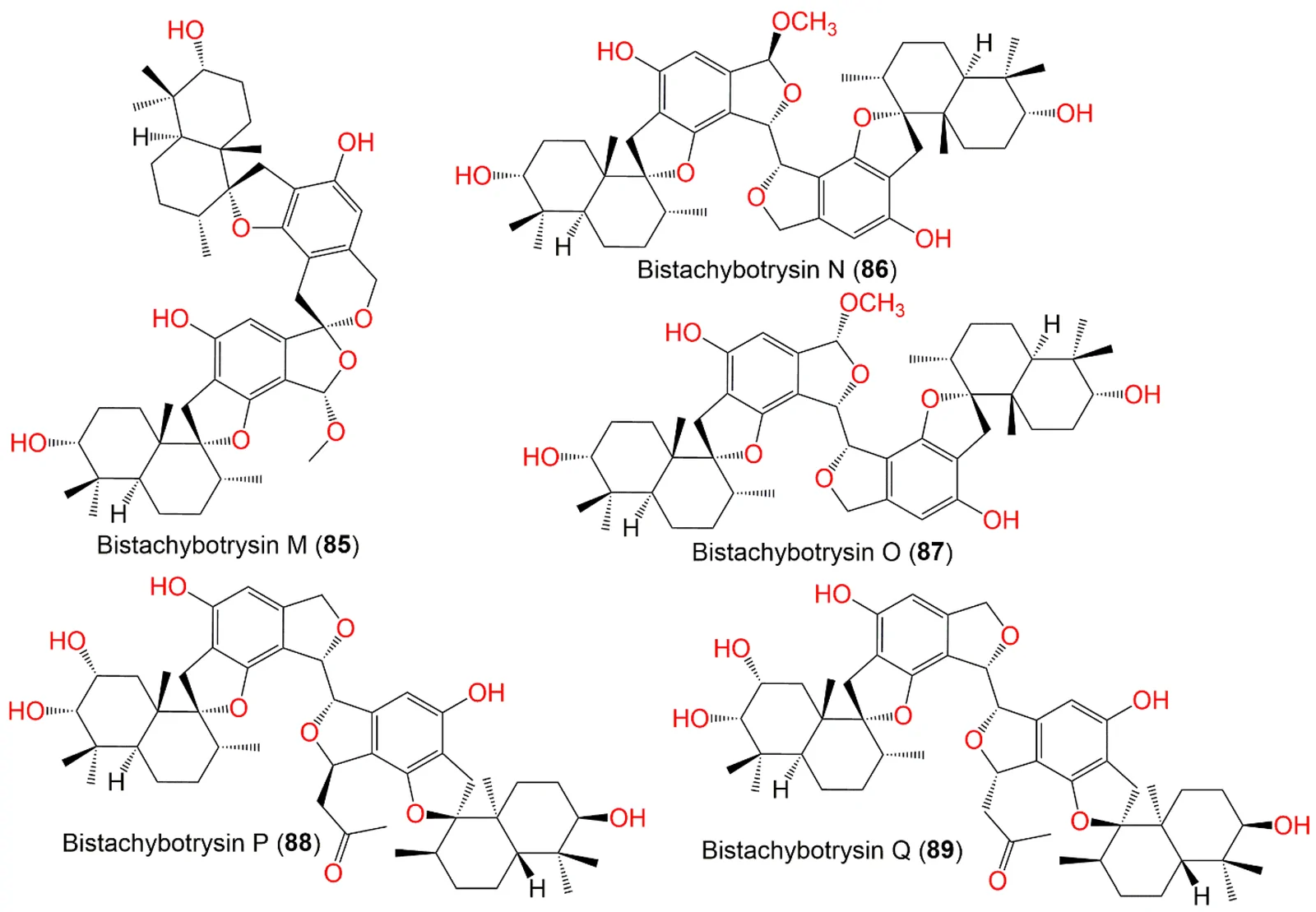
Structures of dimeric phenylspirodrimane derivatives (**85**–**89**) reported from *S*. *chartarum*.

Bistachybotrysin K (**83**) is a new phenylspirodrimane dimer with a central 6/7 oxygen heterocyclic core. It displayed potent cytotoxic capacity versus NCI-H460, HCT116, Daoy, BGC823, and HepG2 (IC_50_ ranged from 1.1 to 4.7 μM) in the MTT assay, revealing its potential as lead for promising anti-tumor [82]. Using SiO_2_ CC and RP-HPLC, novel dimeric phenylspirodrimanes, bistachybotrysins L–V (**84**–**94**), were separated from the mycelia broth and extracts. Their structures and configurations were secured utilizing spectroscopic, X-ray, and ECD analyses (Figure 10). 

**Figure 11:**
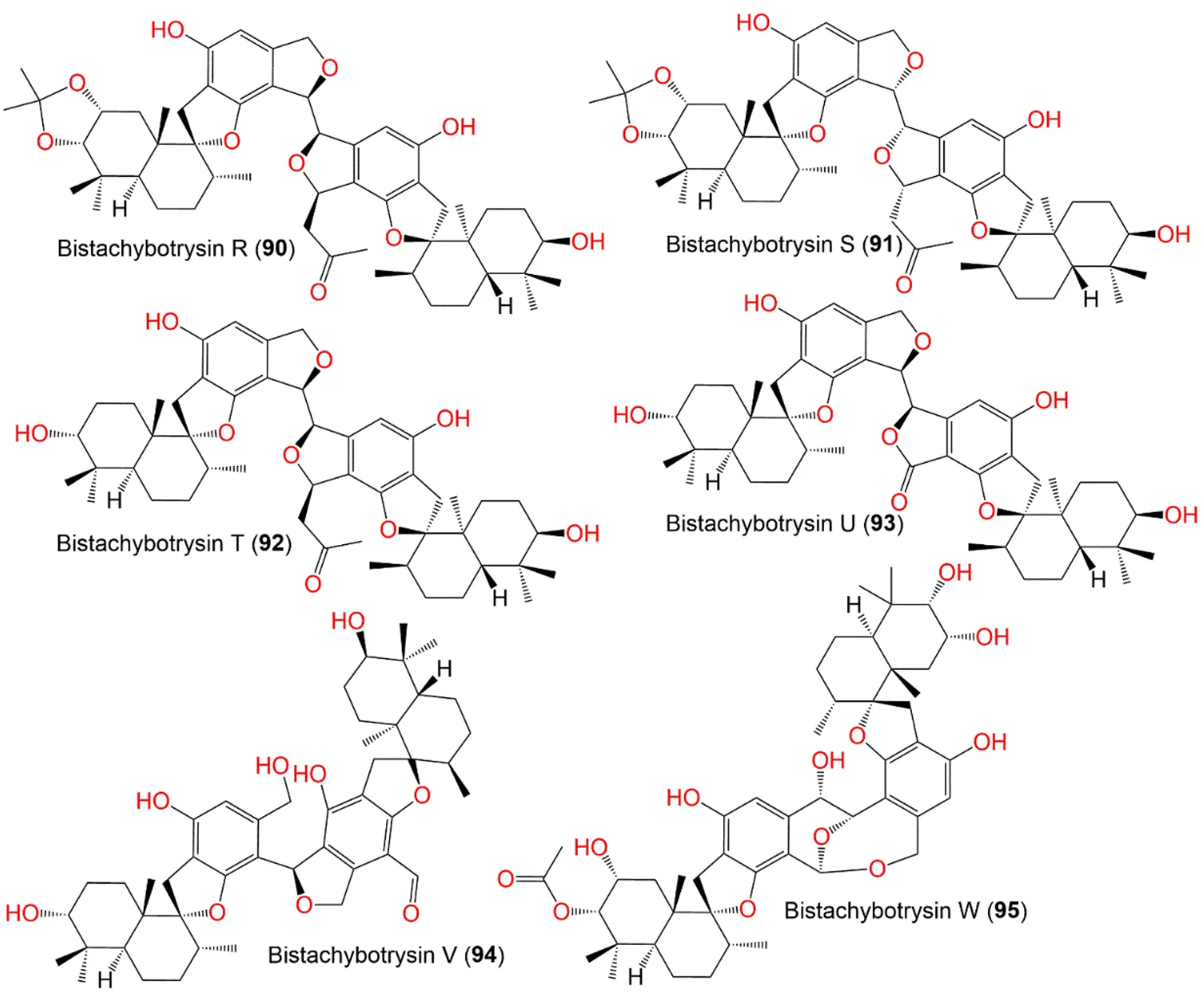
Structures of dimeric phenylspirodrimane derivatives (**90**–**95**) reported from *S*. *chartarum*.

Compounds **84** and **85** are stereoisomeric dimers possessing an unusual [5,6]-spiroketal aromatic skeleton with a 2-methoxy-1,6-dioxaspiro[4,5]decane central core fused with 2 phenyl moieties. On the other side, **86**–**92** possess two linked-furan rings, whereas **93** and **94** featured a rare lactone/furan unit and one furan ring linkages, respectively (Figure 11). It is noteworthy that **91** and **92** selectively inhibited the proliferation of NCI-H460, HCT116, Daoy, BGC823, and HepG2 cell lines (IC_50_ ranged from 1.8 to 11.8 μM) in the MTT assay. 

Furthermore, **85**, **86**, and **92** (Conc. 10 μM) revealed potent neuroprotection potential versus glutamate-caused toxicity in SK-N-SH cells through raising cell viability (17.4, 17.6, and 17.4%, respectively), comparable to resveratrol (16.1% at Conc. 10 μM). Additionally, **91** possessed anti-inflammatory potential by repressing LPS-produced NO production in BV2 cells (inhibition rate 54.2% at Conc. 10 μM) relative to curcumin (67.6%) at the same concentration [83].

**Figure 12:**
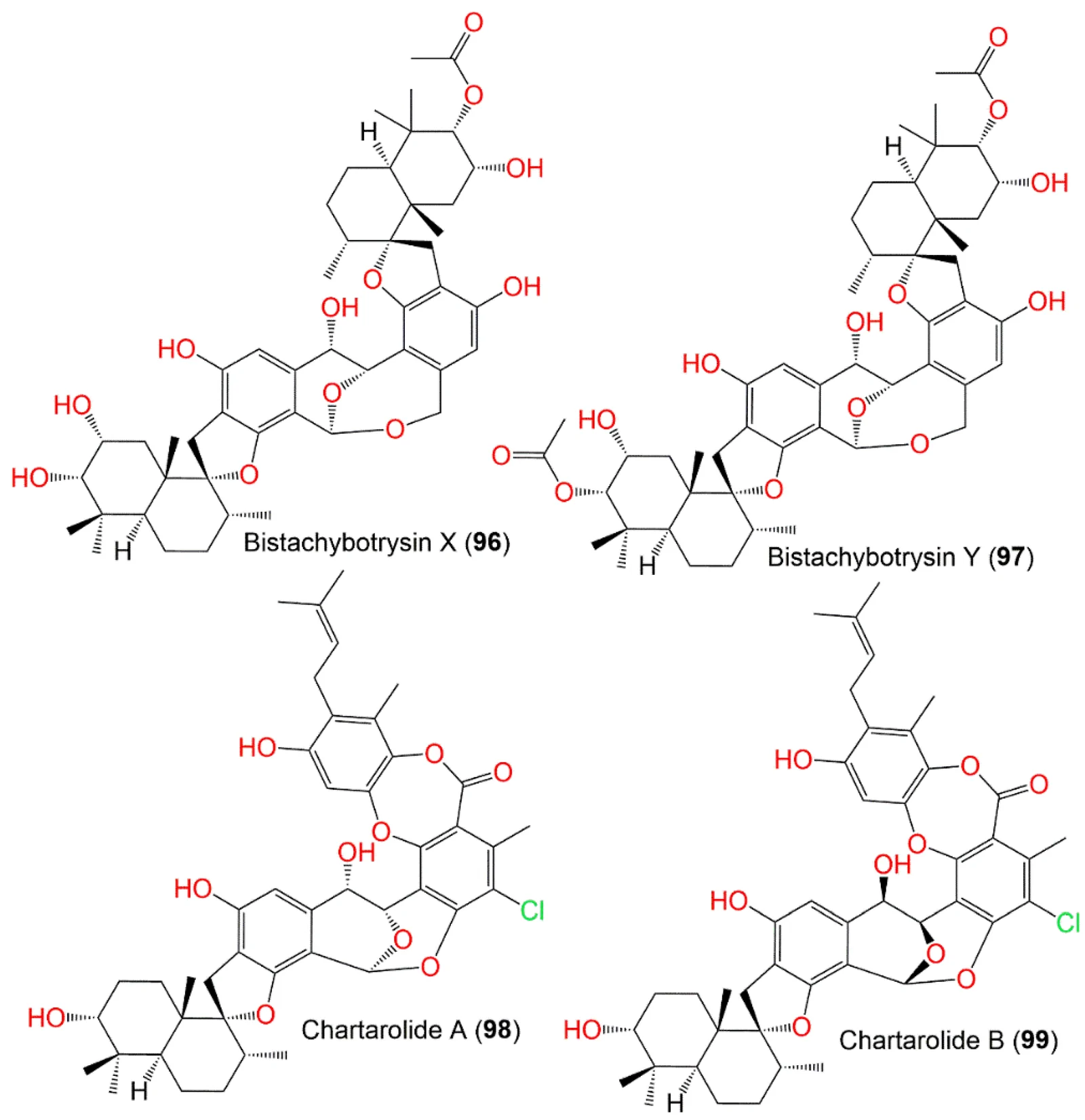
Structures of dimeric phenylspirodrimane derivatives (**96**–**99**) reported from *S*. *chartarum*.

Liu et al. reported the separation of new phenylspirodrimane dimers, bistachybotrysins W–Y (**95**–**97**), having 6/7 oxygen heterocyclic unit using SiO_2_ and Sephadex CC and HPLC that were characterized through spectroscopic and ECD analyses (Figure 12). Compounds **95** and **97** demonstrated selective cytotoxic potential versus HepG2, Daoy, and HCT116 cell lines (IC_50_ 5.9–12.1 μM) [84].

Novel phenylspirodrimanes chartarolides A–C (**98**–**100**) were purified from *S. chartarum* isolated from *Niphates recondite* by SiO_2_ andRP-18 CC and RP-HPLC and assigned relying on spectroscopic analyses, optical rotation, and TDDFT-ECD calculation. Compounds **98** and **99** possess mollicelin J and phenylspirodrimane linked via dioxabicyclononane core formation. These metabolites exerted notable prohibition versus HCT-116, BGC-823, HepG2, A2780, NCIH1650, and MCF7 (IC_50_ ranged from 1.4 to 12.5 µM) in the MTT assay. Compound **99** (IC_50_ 1.6–3.8 µM) had weaker potential than **98** (IC_50_ 1.3–1.9 µM) versus HCT- 116, BGC-823, HepG2, A2780, and MCF7, revealing the direct influence of the dioxabicyclononane moiety‘s configuration on the activity. Additionally, they possessed inhibitory effectiveness versus tumor-linked kinases, FGFR3, IGF1R, PDGFRb, and TrKB (IC_50_s 2.6–21.4 µM), compared to satratoxin H (**144**) (IC_50_ ˂0.5 µM) [85].

**Figure 13:**
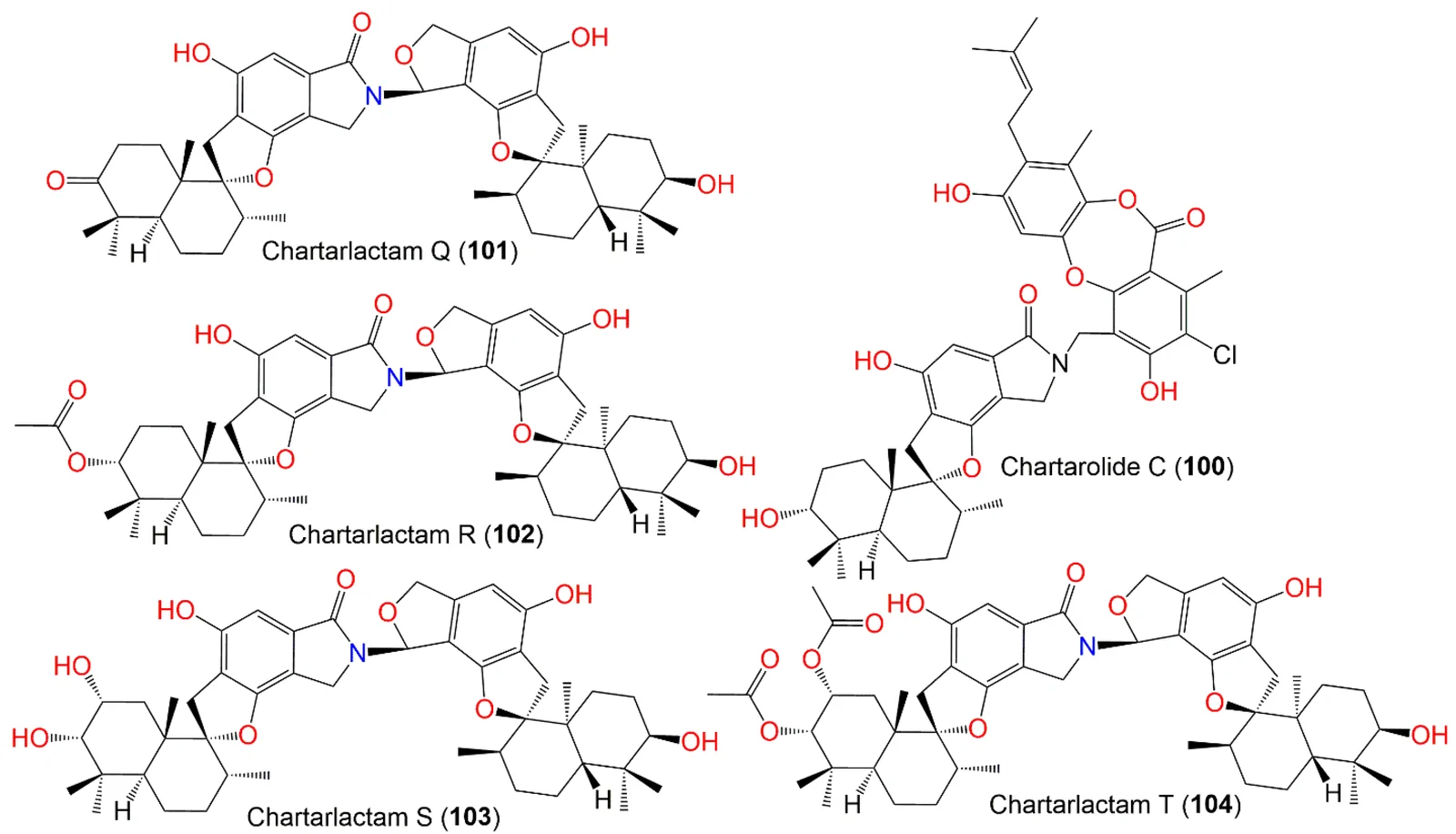
Structures of dimeric phenylspirodrimane derivatives (**100**–**104**) reported from *S*. *chartarum*.

In 2020, Liu et al. purified new dimeric derivatives, chartarlactams Q–T (**101**–**104**), in addition to **109** from the broth of a sponge-derived *S. chartarum* WGC-25 C-6 using SiO_2_ and ODS CC and HPLC (Figure 13). Their structures and configurations were specified by various spectral tools, along with ECD, optical rotation, and Mosher’s method. These metabolites have two N–C linked phenylspirodrimane units without an alkyl chain. 

**Scheme 7:**
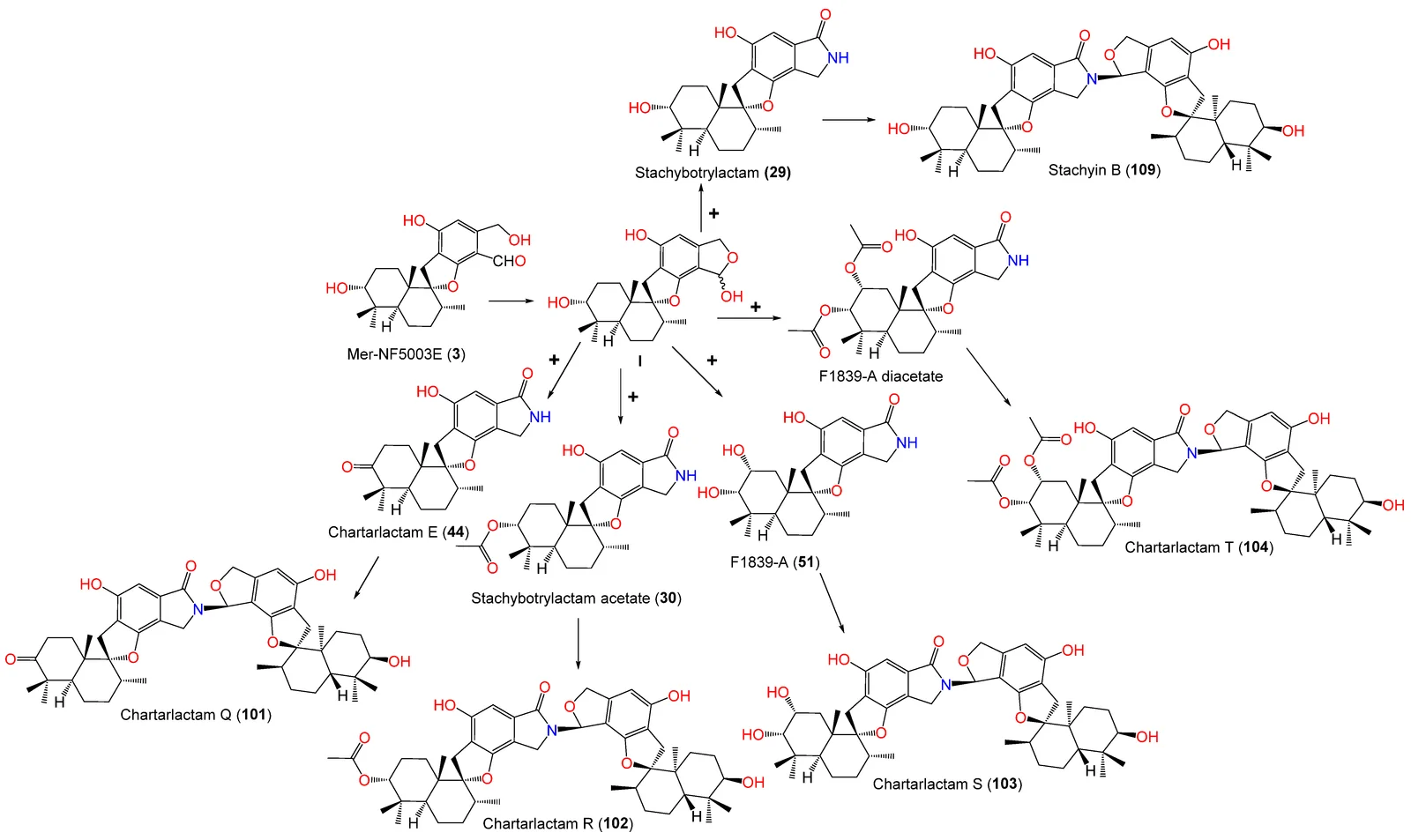
Biosynthetic pathway of chartarlactams Q–T (**101**–**104**) and stachyin B (**109**) [86,88].

Compounds **101**–**103** and **109** possessed inhibitory potential versus *S. aureus* (MIC 8, 16, 4, and 4 μg/mL, respectively), compared with chloramphenicol (MIC 1.0 μg/mL). Additionally, their anti-ZIKV virus capacity was assessed on ZIKV (African-lineage MR766 strain) infected A549 cells. It was found that **104** significantly prohibited NS5 and E protein expression (conc. 10 μM), whereas **101**–**103** and **109** were inactive. Envelope (E) protein in ZIKV virus has a remarkable role in membrane fusion and binding to target the host cells receptors, whereas NS5 protein a ZIKV genome encoding non-structural proteins that serves as template for the (+)ssRNA genomic RNA synthesis and viral polyprotein synthesis. Hence, this metabolite prohibited ZIKV virus by multi-targets to block RNA replication and viral entrance [86]. Biosynthetically, they are generated from **3** that undergoes nucleophilic cyclization to give **I** (hemiacetal intermediate). The latter reacts by dehydroxylation with various monomers (chartarlactam E (**44**), stachybotrylactam acetate (**30**), F1839-A (**51**), F1839-A diacetate, and stachybotrylactam (**29**), respectively) to produce **101**–**104** and **109** (Scheme 7).

**Figure 14:**
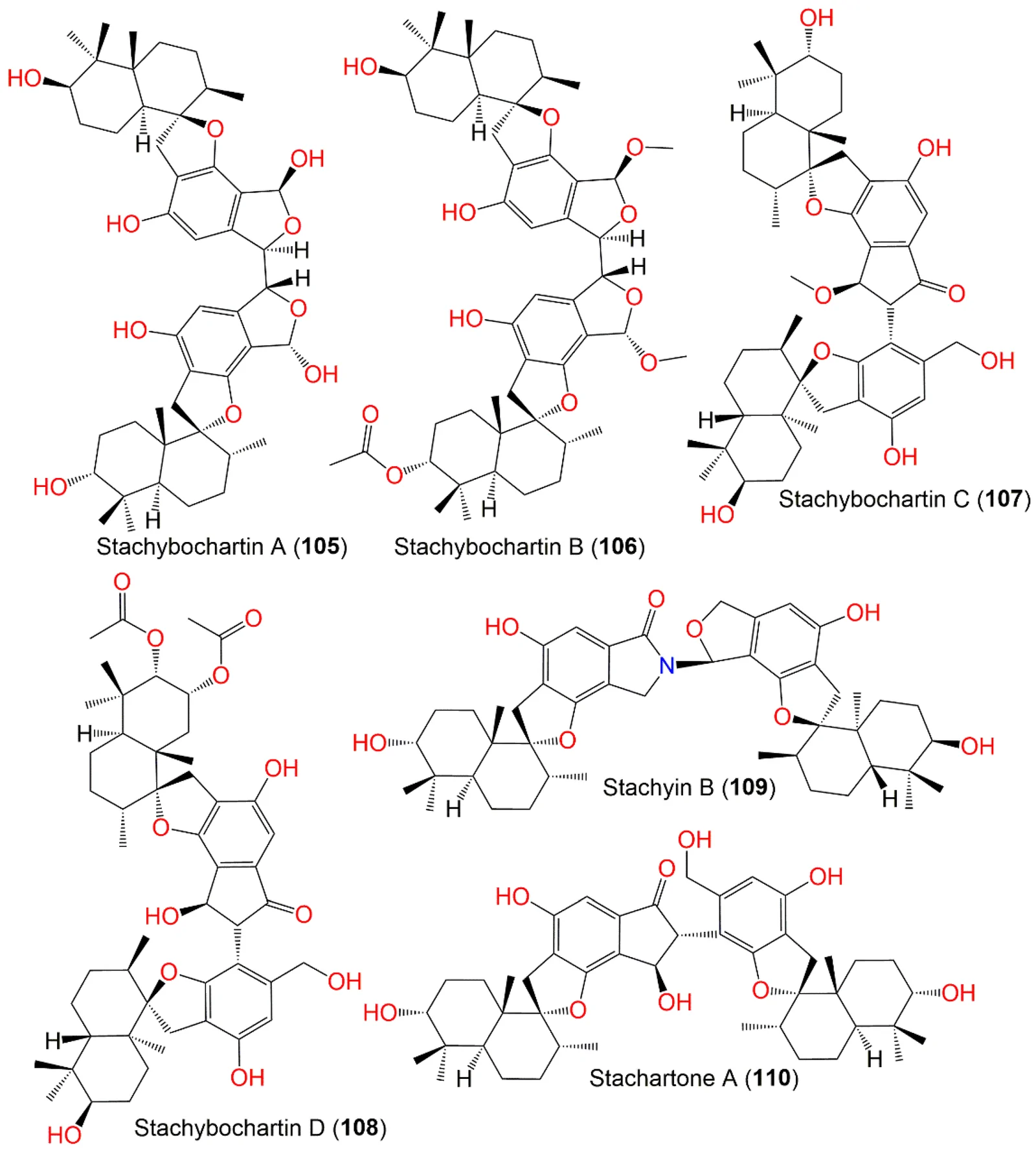
Structures of dimeric phenylspirodrimane derivatives (**105**–**110**) reported from *S*. *chartarum*.

*S. chartarum*-associated with *Pinellia ternata* biosynthesized undescribed phenylspirodrimane derivatives, stachybochartins A (**105**), B (**106**), C (**107**), D (**108**), E (**25**), F (**33**), and G (**15**), that were separated using SiO_2_ CC and RP-HPLC (Figure 14). 

Their assignments and configurations were accomplished through spectroscopic analysis as well as modified Mosher’s and ECD-calculations/ECD-exciton chirality methods. Compounds **105**–**108** are uncommon C–C coupled dimeric derivatives, where the monomeric units are C7’-C7’’’- (e.g., **105** and **106**) and C5’’’-C8‘‘-coupled (e.g., **107** and **108**). On the other side, Stachybochartin G (**15**) possessed a seco-bisabosqual framework with 3’S/6’R/7’S configuration. Further, their cytotoxic potential was assessed versus MDA-MB-231, MCF-7, and U-2OS cell lines in the MTT assay. It was found that **15** and **105**–**108** had cytotoxic potential versus U-2OS and MDA-MB-231 (IC_50_s 4.5–21.7 µM). Besides, **15** and **107** demonstrated powerful anti-proliferation and anti-apoptosis effectiveness versus U-2OS cells, while all metabolites had no influence (IC_50_ >50 µM) versus MCF-7 cells [70]. These data may provide evidence for further research into the anticancer activities of these compounds.

**Figure 15:**
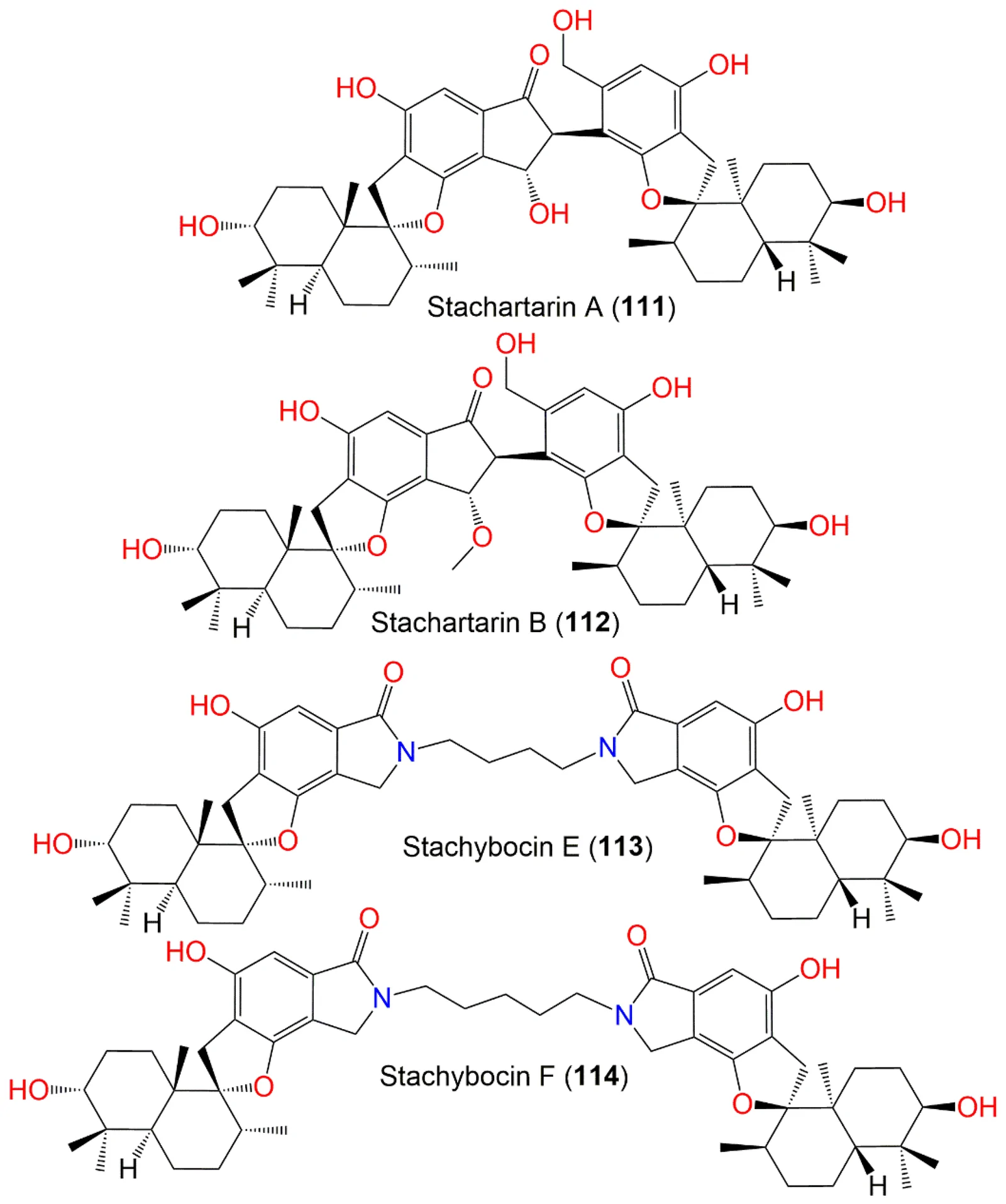
Structures of dimeric phenylspirodrimane derivatives (**111**–**114**) reported from *S*. *chartarum*.

A new phenylspirodrimane dimer, stachartarin A (**111**), was separated from tin mine tailings associated *S. chartarum* culture and elucidated by means of spectroscopic methods (Figure 15). 

**Scheme 8:**
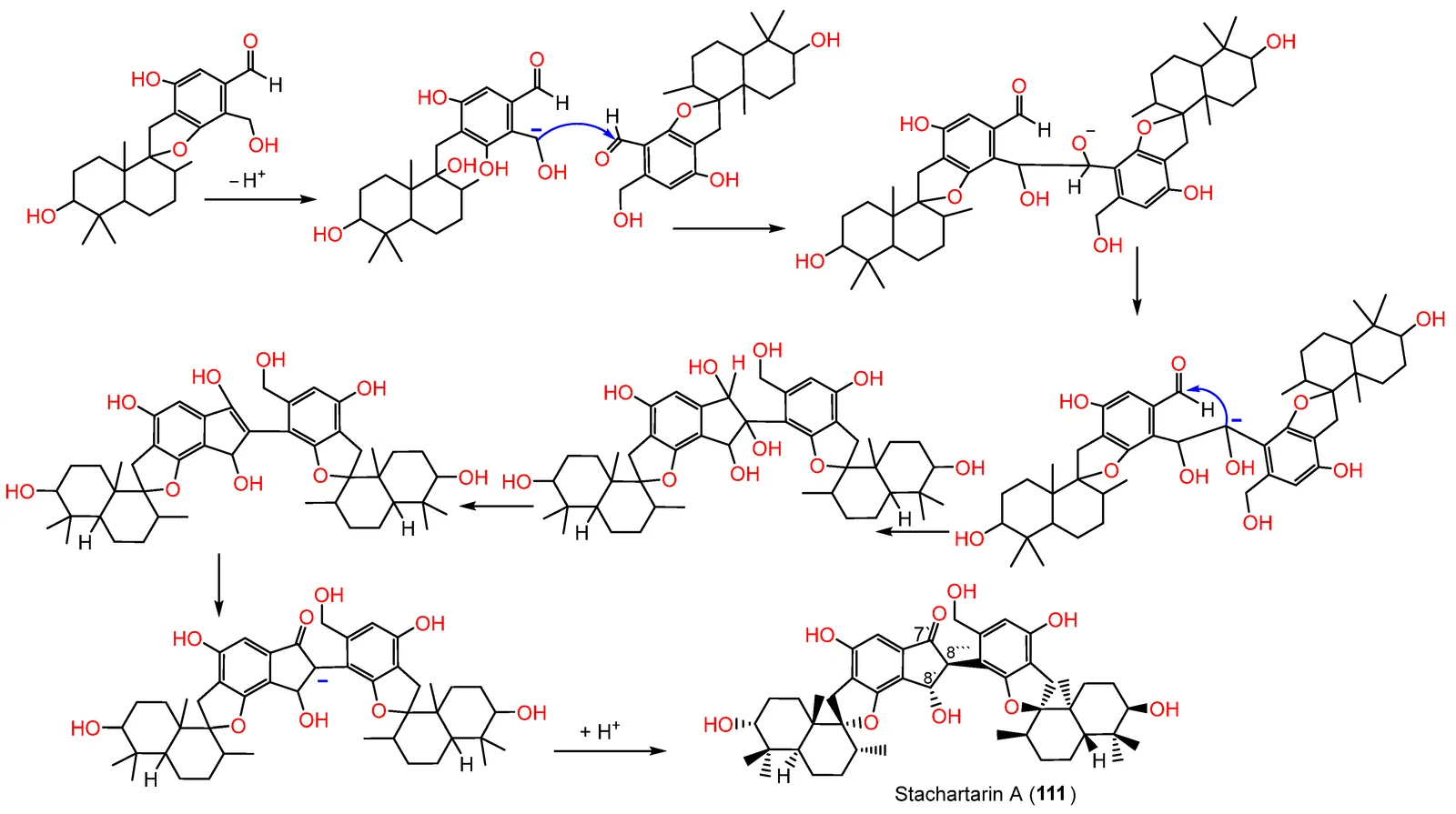
Biosynthetic pathway of stachartarin A (**111**) [88].

It differed from stachartin B (**28**) by the absence of a lactone group in **28** and the existence of an oxymethylene and ketone carbonyl in **111**. Compound **111** had (IC_50_ >40 μM) no remarkable effectiveness versus MCF-7, HL-60, SMMC-7721, SW480, and A-549 cells in the MTT assay [88]. Ding et al. proposed that **111** is biosynthesized from two phenylspirodrimane derivative units through a reduction–oxidation reaction by C-C bond linkage among C-7′/C-8‴ and C-8′/C8‴, respectively, as illustrated in Scheme 8 [88].

### 3.2. Trichothecenes 

Trichothecenes are sesquiterpenoid-related mycotoxins commonly produced by fungi. They possess a marked cytotoxic potential versus eukaryotic organisms through the prohibition of DNA and protein synthesis as well as mitochondrial electron transport system [92]. They have common structural features: 12,13-epoxide moiety, 9,10-double bond, and various ring oxygenation patterns [117]. Structurally, they involve three main groups: trichothecenes type A, having hydrogen, hydroxyl, or isovaleryl functionalities at C-8; trichothecenes type B, having a C-8 carbonyl group; and macrocyclic trichothecenes, having a cyclic di or tri-ester ring binding C-4 to C-15 [118,119]. Macrocyclic trichothecenes are the most powerful toxic group [120].

**Figure 16:**
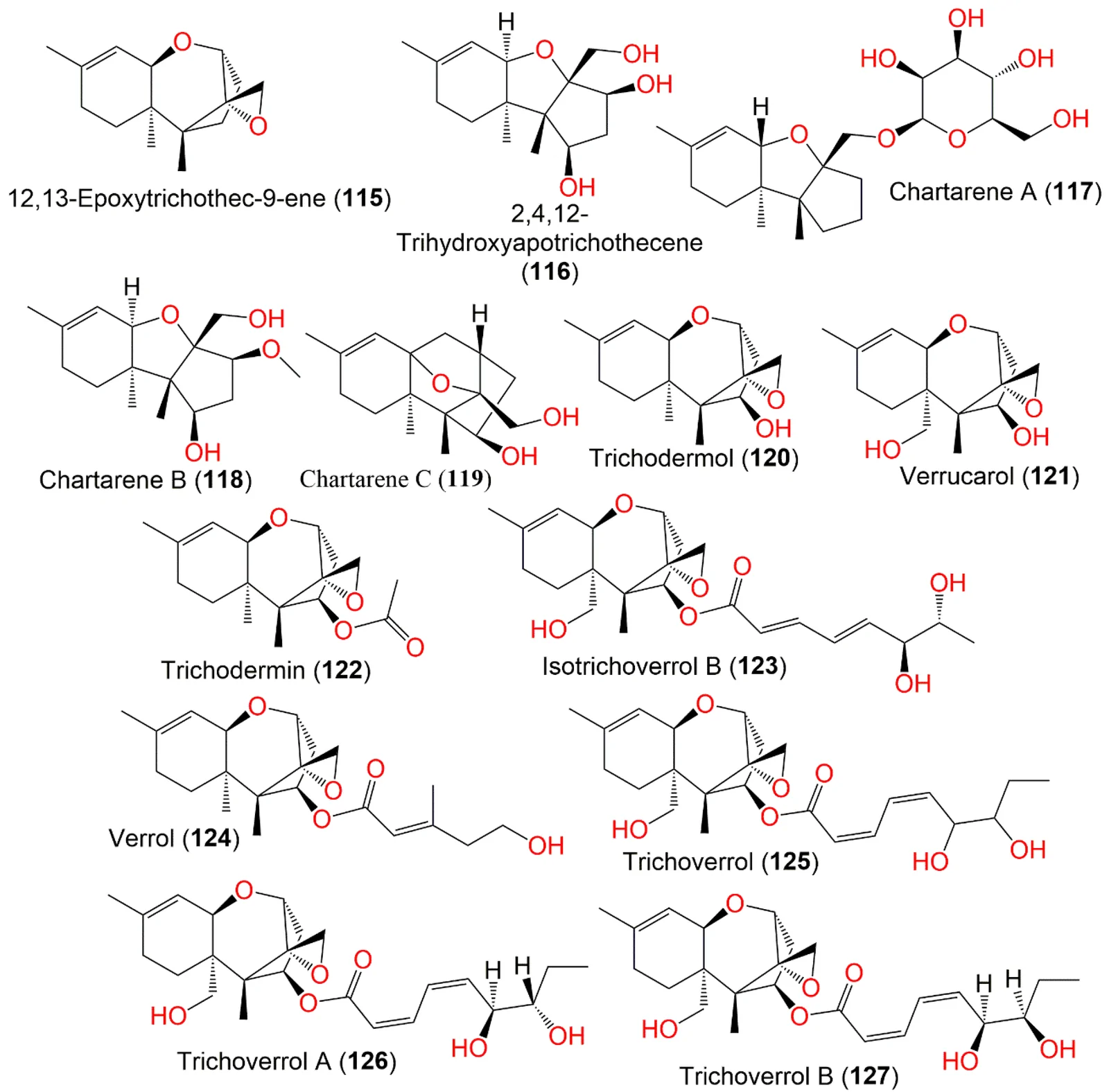
Structures of trichothecenes (**115**–**127**) reported from *S*. *chartarum*.

**Figure 17:**
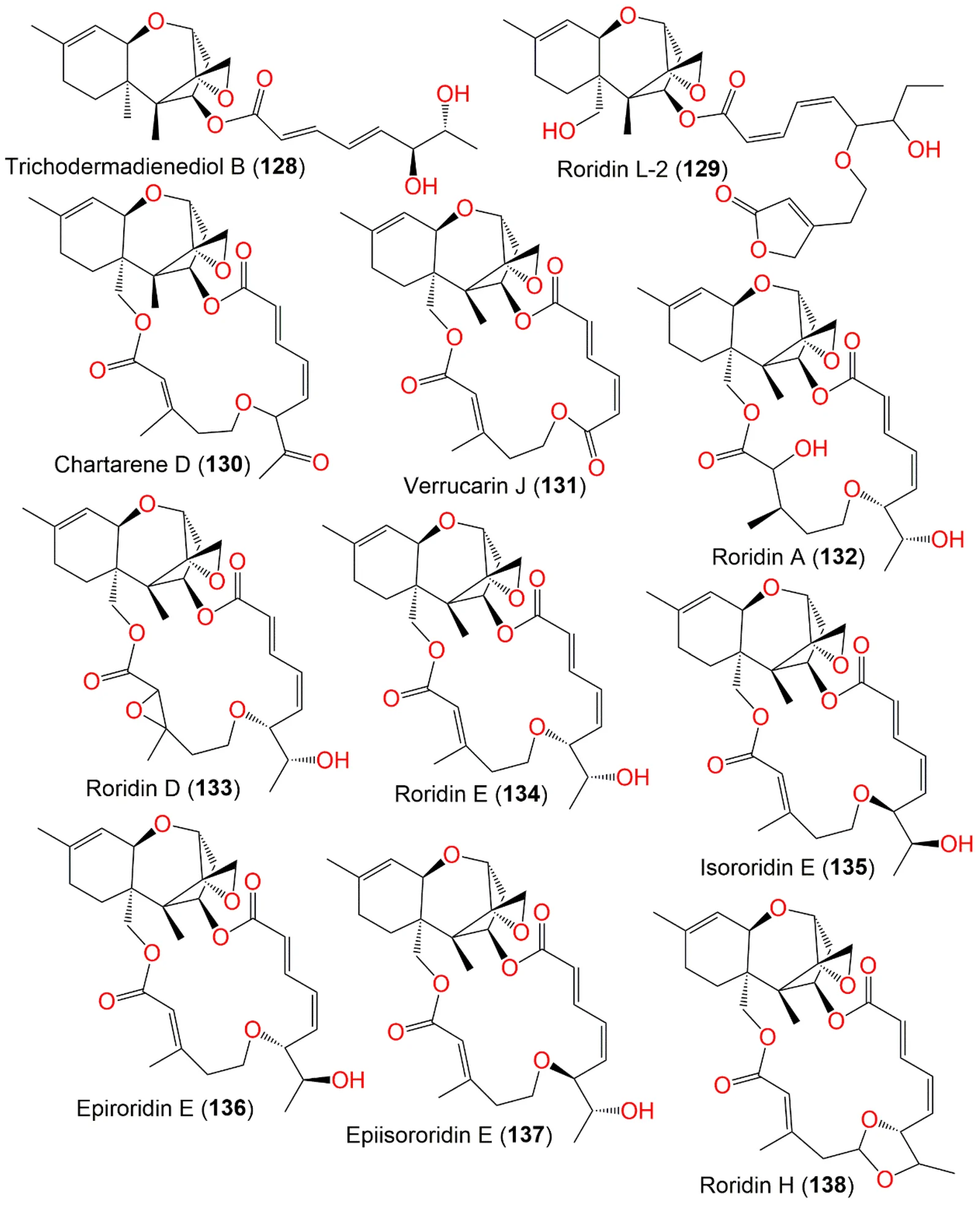
Structures of trichothecenes (**128**–**138**) reported from *S*. *chartarum*.

Bio-guided separation of the EtOAc fraction of *Niphates recondite*-associated *S. chartarum* using SiO_2_ and RP-18 CC and HPLC afforded four new trichothecene-related sesquiterpenes, chartarenes A–D (**117**–**119** and **130**), and eleven known analogs, **116**, **120**, **123**, **124**, **128**, **129**, **134**, **139**, **142**, **144**, and **146,** that were verified based on spectroscopic and X-ray analyses in addition to chemical methods [92] (Figure 16 and Figure 17). 

**Figure 18:**
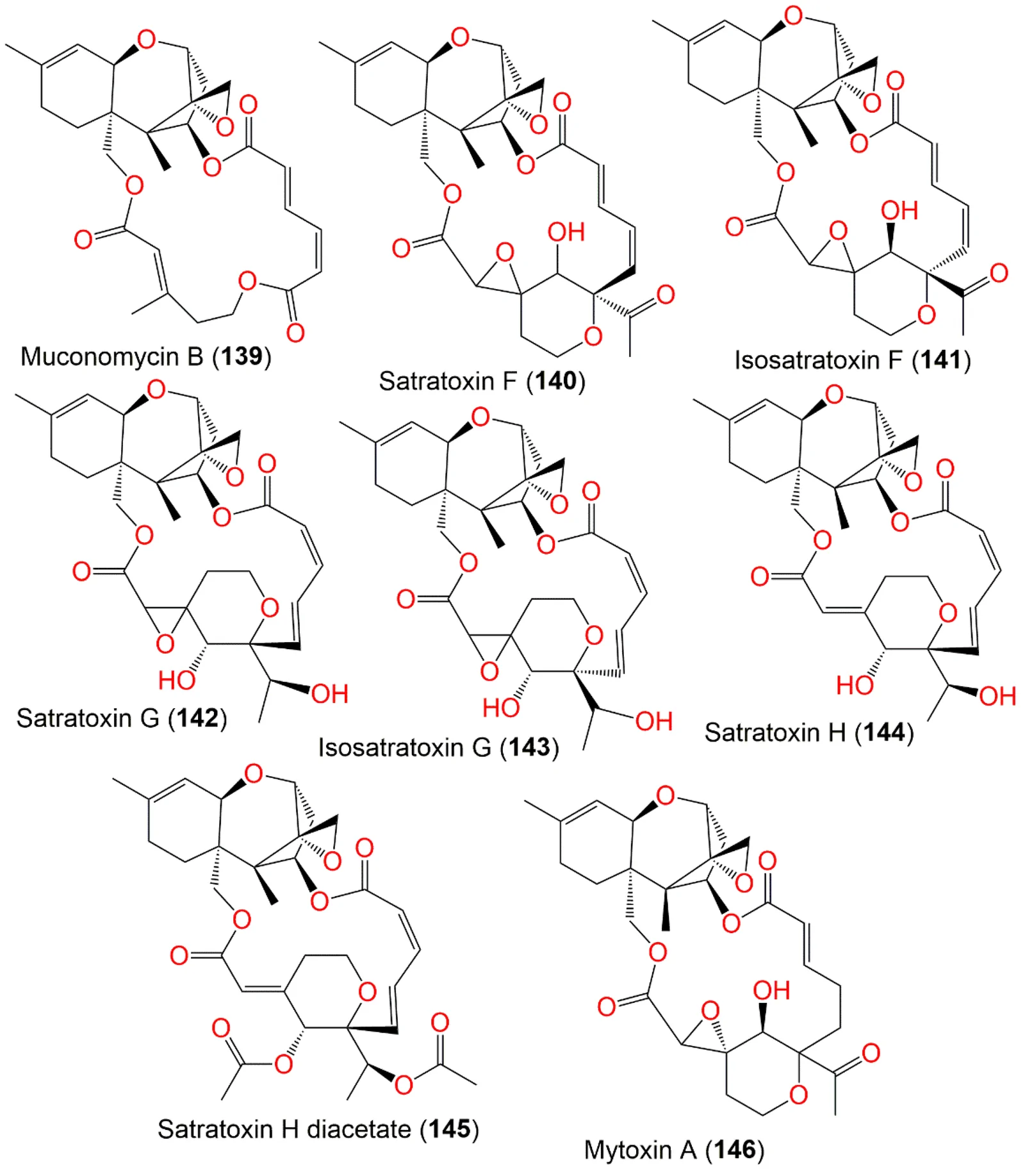
Structures of trichothecenes (**139**–**146**) reported from *S*. *chartarum*.

They were tested versus a panel of human cancer cell lines (e.g., HCT-116, HepG2, BGC-823, NCI-H1655, and A2780) in the MTT method. It is noteworthy that they had selective inhibitory influences versus all cell lines with **134, 142**, **144**, and **146** (IC_50_ < 10 nM) having potent effectiveness (Figure 18). The structure–activity relation study demonstrated that this effect is related to the scaffolds and substitution pattern. 

The apotrichothecene-related analogs, **117** with a C-12 sugar and without C-4 and C-2 hydroxy group (IC_50_ 2.38–3.95 µM) or **118** with a C-2 methoxy group, had a weaker influence than that of the 2,4,12-trihydroxy analog, **116** (IC_50_ 0.65–0.87 µM). On the other side, trichodermol-type analogs, having a macrocyclic ring alongside a tetrahydropyran ring (e.g., **142**, **144**, and **146**), possessed remarkable potential; however, those without a tetrahydropyran-attached macrocyclic moiety (**130** and **139**) exhibited less cytotoxicity. Compounds **123**, **124**, **128**, and **129** with a C-14 or C-4 linear moiety had reduced effects compared with **146**. Additionally, their antitumor mechanism was investigated versus tumor growth-related tyrosine kinases (e.g., FGFR3 (fibroblast growth factor receptor 3), IGF1R (insulin-like growth factor 1 receptor), PDGFRb (β-type platelet-derived growth factor receptor), and TRKB (tropomyosin receptor kinase B)). They were found to exert powerful inhibitory action, except for **117** and **123,** having a weak activity (IC_50_ > 20 μM). FGF signaling has a role in various disorders such as cancer, involving anti-apoptosis, proliferation, angiogenesis, drug resistance, and invasion. Therefore, targeting FGFR is a promising topic in the clinical oncology field [92]. 

Satratoxin G (**142**) and its biosynthetic precursor **129** were isolated from the culture acetonitrile extract using Michel–Miller SiO_2_ CC and HPLC and characterized by ESI-CID (electrospray/ionization/collision-induced/dissociation). Compound **142** had a C-4 to C-15-linked cyclic ester ring, however, **129** possessed a C-4 extended carbon chain and no C-15-substituent. Satratoxin G was found to cause apoptosis of the nose and brain OSN (nasal-olfactory sensory neurons) of mice upon intranasal exposure [121]. Additionally, it induced apoptosis in PC-12 (neuronal cell model) through marked upregulation of p53, BAX, and PKR [122]. In 2009, Islam et al. compared the neurotoxic potential of both metabolites in vivo and in vitro. The study revealed that **142** (conc. 10 to 25 ng/mL) caused PC-12 cells apoptotic death, while **129** (Conc. up to 1000 ng/mL) had no effect using Alamar blue, flow cytometry, and agarose DNA fragmentation assays. Similarly, **142** (dose 100 mg/kg/body weight, intra-nasal) produced remarkable OSN apoptosis and olfactory epithelium atrophy, whereas **129** showed no influence at the same dose. These results suggested that **129** had a weak potential to adversely affect health in comparison to **142** [98].

Satratoxin F (**140**) and satratoxin H (**144**) were separated by Yang et al. using RP-18 and HPLC CC. Compound **140** displayed moderate inhibitory activity against methicillin-resistant *S. aureus* ATCC-29213 (MIC 39 µg/mL) [73]. Besides, **140** (IC_50_s < 39 nM) displayed strong cytotoxic potential versus MDA-MB-231, C4-2B, MGC803, MDA-MB-468, and A549 cell lines in the CCK-4 assay [73].

### 3.3. Isoindoline derivatives

**Figure 19:**
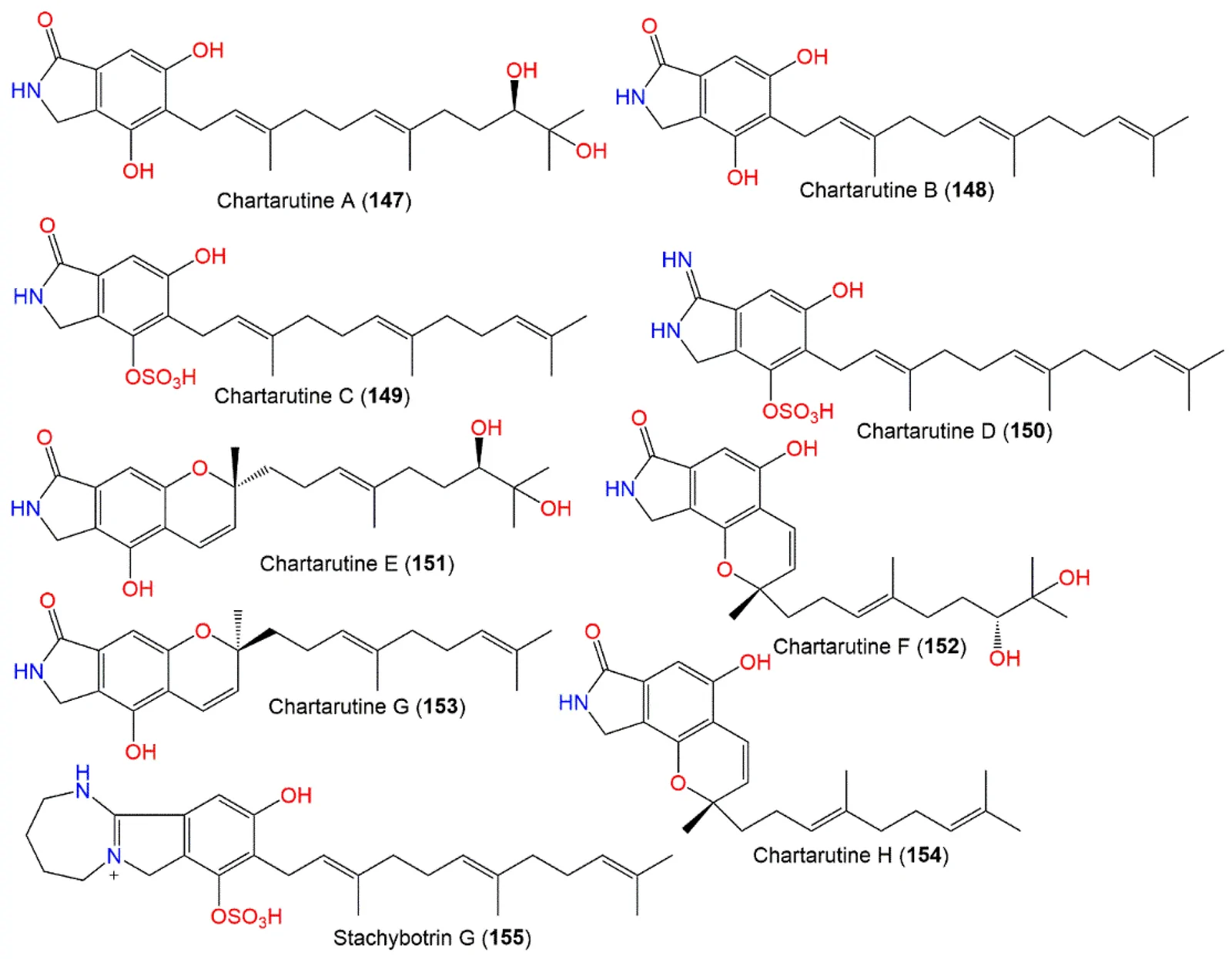
Structures of isoindoline derivatives (**147**–**155**) reported from *S*. *chartarum*.

Mai et al. isolated **155** using SiO_2_ and C18 ODS CC of the mycelia EtOAc extract *S. chartarum* MXH-X73-associated with *Xestospongia testudinaris* that was characterized by HRESIMS, NMR, and X-ray. This metabolite is a cyclized iminoisoindoline, having farnesylated decahydropyrrolo[1,2-a][1,3]diazocine moiety (Figure 19). 

**Scheme 9:**
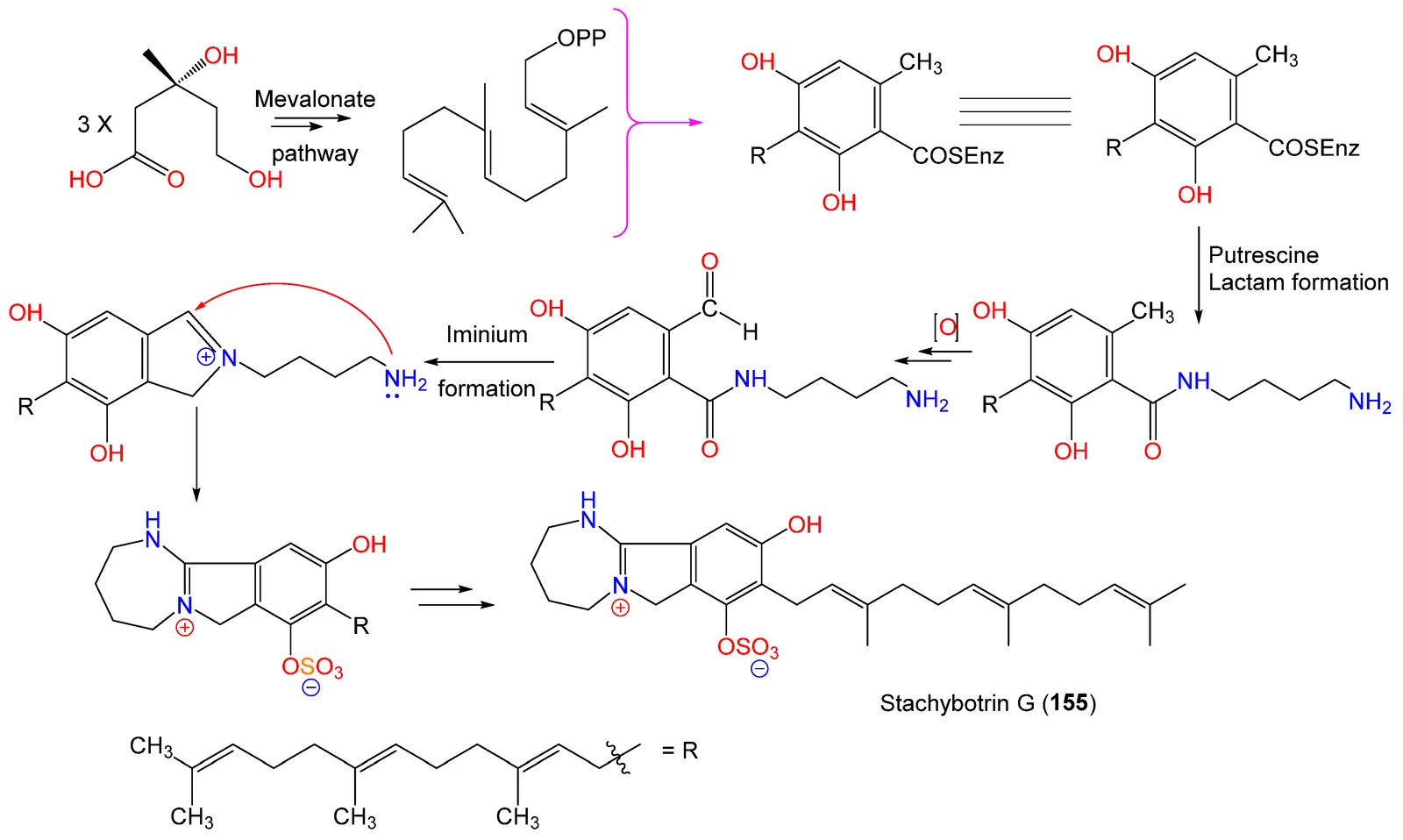
Biosynthetic pathway of stachybotrin G (**155**) [105].

It had no cytotoxic (versus P388, A-549, HL-60, BEL-7402, and Hela cells), antiviral (versus HIV and H1N1), and antibacterial (versus *M. phlei*, *S. aureus*, *Colibacillus* sp., and *Blastomyces albicans*) potential [105]. Ma et al. proposed that **155** is generated through both mevalonate and polyketide pathways, with subsequent modifications, including amidation, cyclizing, and sulfation (Scheme 9).

Li et al. purified eight new derivatives: chartarutines A–H (**147**–**154**) from the mycelia EtOAc extract of *Niphates* sp.-associated *S*. *chartarum* using SiO_2_ and RP-18 CC and HPLC that were elucidated by spectroscopic tools, along with Mosher method and CD analysis. Compound **148** is 1’,11’-dehydroxy-10’,11’-ene derivative of **147**, whereas **150** featured an isoindolinimine unit instead of an isoindolamine unit in **149**. Besides, **151**/**153** and **152**/**154** had pyrano-isoindolinone rings linked to C5-C-3’ and C-3-C-3‘, respectively. Compounds **147**–**154** revealed anti-HIV potential (IC_50_s 4.90–74.00 μM), whereas **148**, **153**, and **154** had the potent effectiveness (IC_50_ 4.90–5.58 μM) in the luciferase assay with lower cell cytotoxicity (CC) than efavirenz (CC_50_ 40 μM), suggesting their further investigation as lower cytotoxic anti-HIV candidates. The structure-activity relation indicated that the variation in side chain directly influenced the inhibitory potential. The analogs having triene (**153**, **154**) or diene (**148**) side-chain had more enhanced potential than vicinal diol analogs (**147**, **151**, and **152**), whereas the variation in scaffold (e.g., **148**, **153**, and **154**) had no influence on the activity. Replacing 3-OH by sulfate (e.g., **149** and **150**) reduced the activity [104].

**Figure 20:**
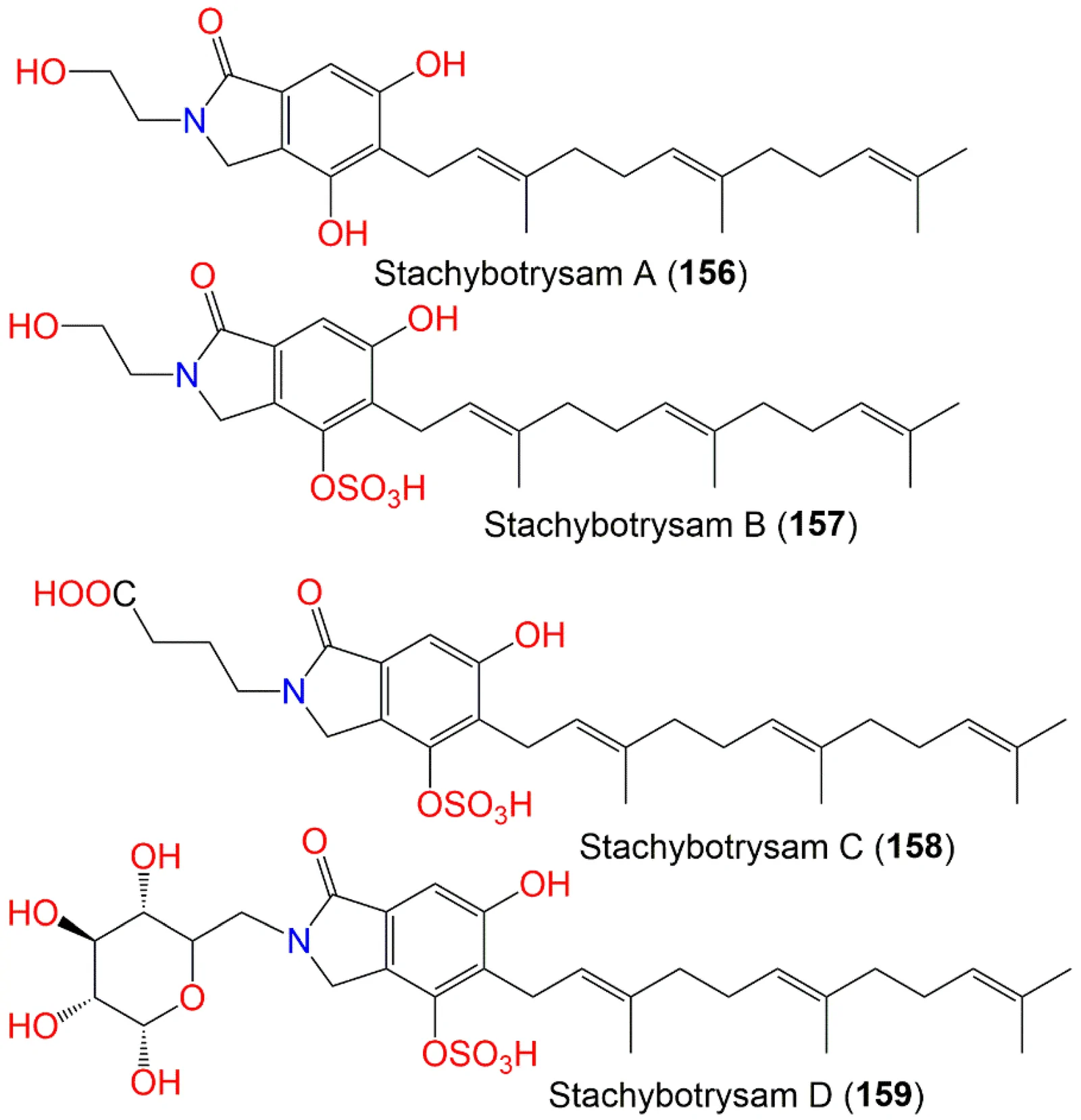
Structures of isoindoline derivatives (**156**–**159**) reported from *S*. *chartarum*.

Stachybotrysams A–D (**156**–**159**), new farnesylated isoindolinone derivatives, and new cyclized-farnesyl, stachybotrysam E (**35**), along with **148** were separated using SiO_2_ CC and HPLC (Figure 20) and elucidated based on spectral data analysis and optical rotation. Compounds **156**–**158** featured prenylated isoindolinone core with N-linked side chain with (e.g., **157** and **158**) or without (e.g., **156**) C-4 sulphate moiety, whereas **159** has N/C-6″ linked glucose moiety. On the other side, **35** has a cyclized farnesyl moiety as chartarlactam N (**68**), except having N-linked butyric acid unit instead of the ethyl alcohol moiety. In the HIV-inhibition luciferase assay, **156**–**158** demonstrated promising inhibitory potential against HIV-1 virus (IC_50_ 9.3, 1.0, and 9.6 μM, respectively) compared to efavirenz (IC_50_ 2.0 nM) using 293T cells. It was reported that the farnesyl chain cyclization reducing the effectiveness and the N-attached side chain, as well as sulphate moiety existence, could influence the activity [76].

### 3.4. Atranones and Dolabellane Diterpenoids

**Figure 21:**
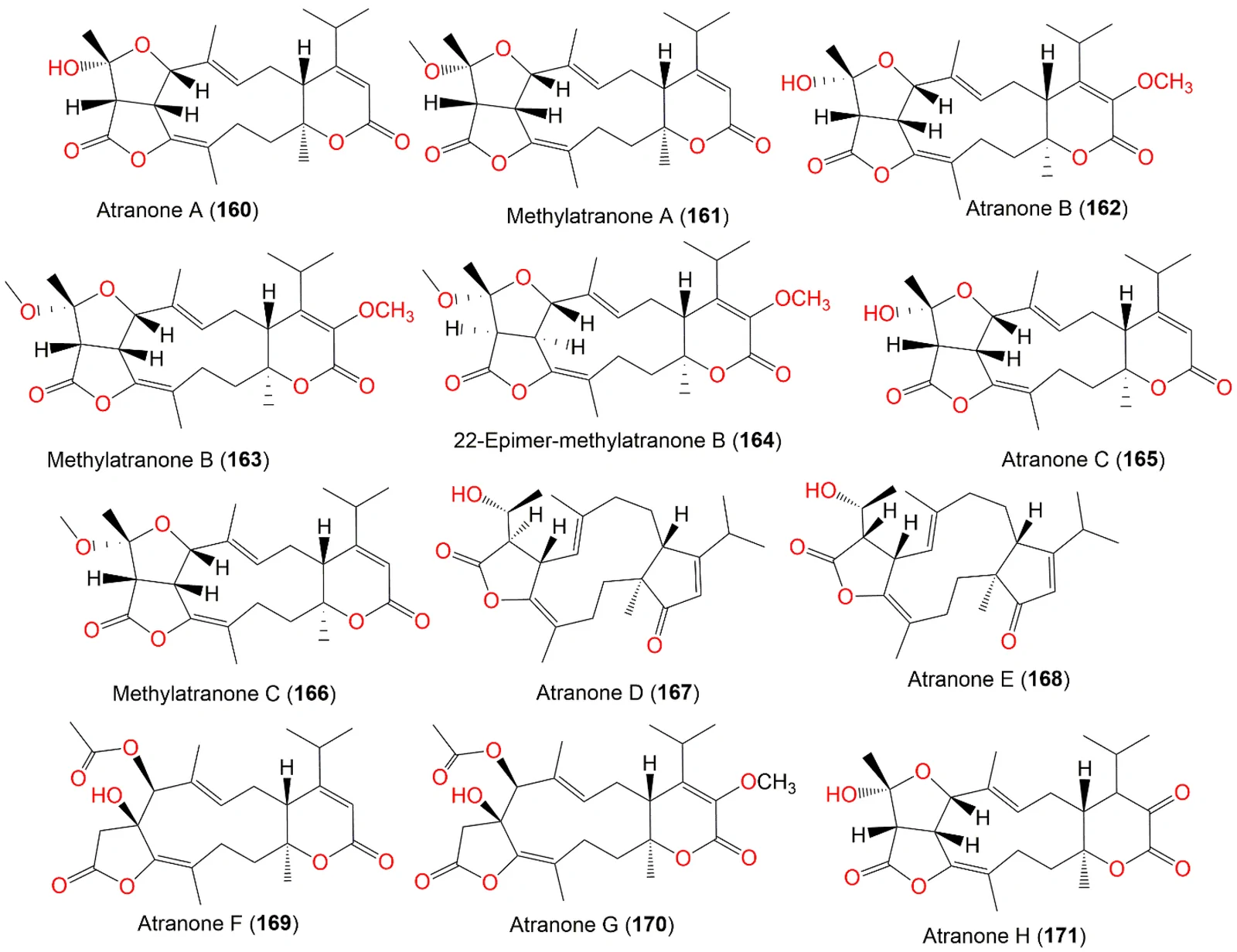
Structures of atranones (**160**–**171**) reported from *S. chartarum*.

Atranones are an uncommon type of C-alkylated dolabellanes that are featured by a 5/11-fused bicyclic carbon skeleton. They comprise three groups: C22, C23, and C24 atranones. Li et al. separated seven new atranones, **164**, **175**–**179**, and **190** in addition to formerly reported analogs **161**–**163,** from soybean and rice culture media CHCl_3_ fraction of *S. chartarum* using Sephadex LH-20 and SiO_2_ CC and HPLC (Figure 21). 

**Figure 22:**
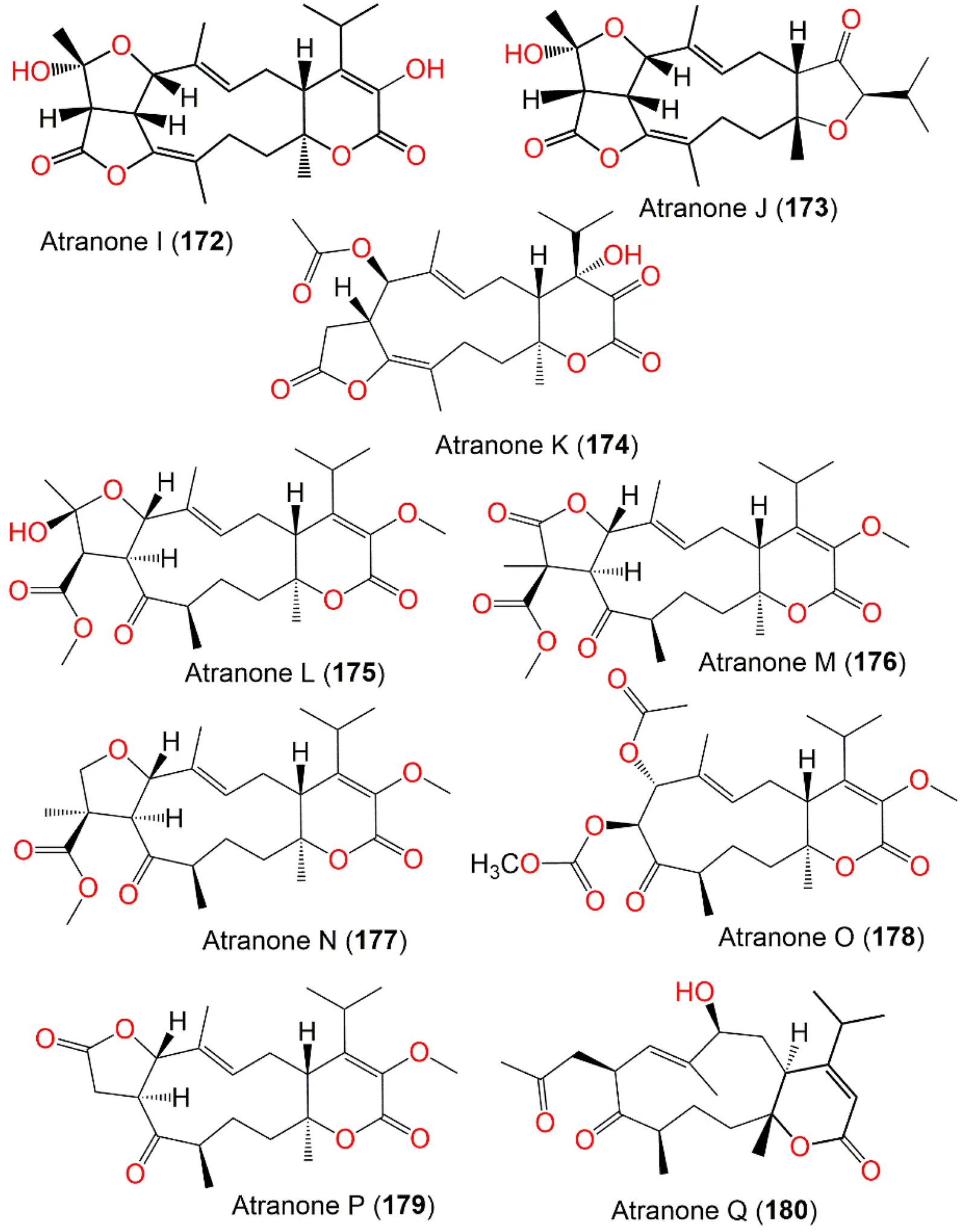
Structures of atranones (**172**–**180**) reported from *S. chartarum*.

Their structures were established by spectral and X-ray analyses, as well as ECD (electronic circular dichroism) calculations and optical rotation. DRG (Dorsal root ganglia) neuron is commonly utilized for assessing the growth capacity of neurons and drugs’ therapeutic potential in peripheral nerve regeneration [108]. For successful peripheral nerve regeneration, the first step is the axonal regrowth from injured neurons. The axonal regeneration potential of **162** was estimated using primary DRG neurons. Compound **162** boosted neurite outgrowth from adult dorsal root ganglia neurons remarkably without influencing cell survival (conc. 1 μg/mL) Besides, **161**–**164**, **175**, **176**, **178**, and **179** (Figure 22) had no cytotoxic potential versus MDA-MB-435 in the MTT [108].

**Figure 23:**
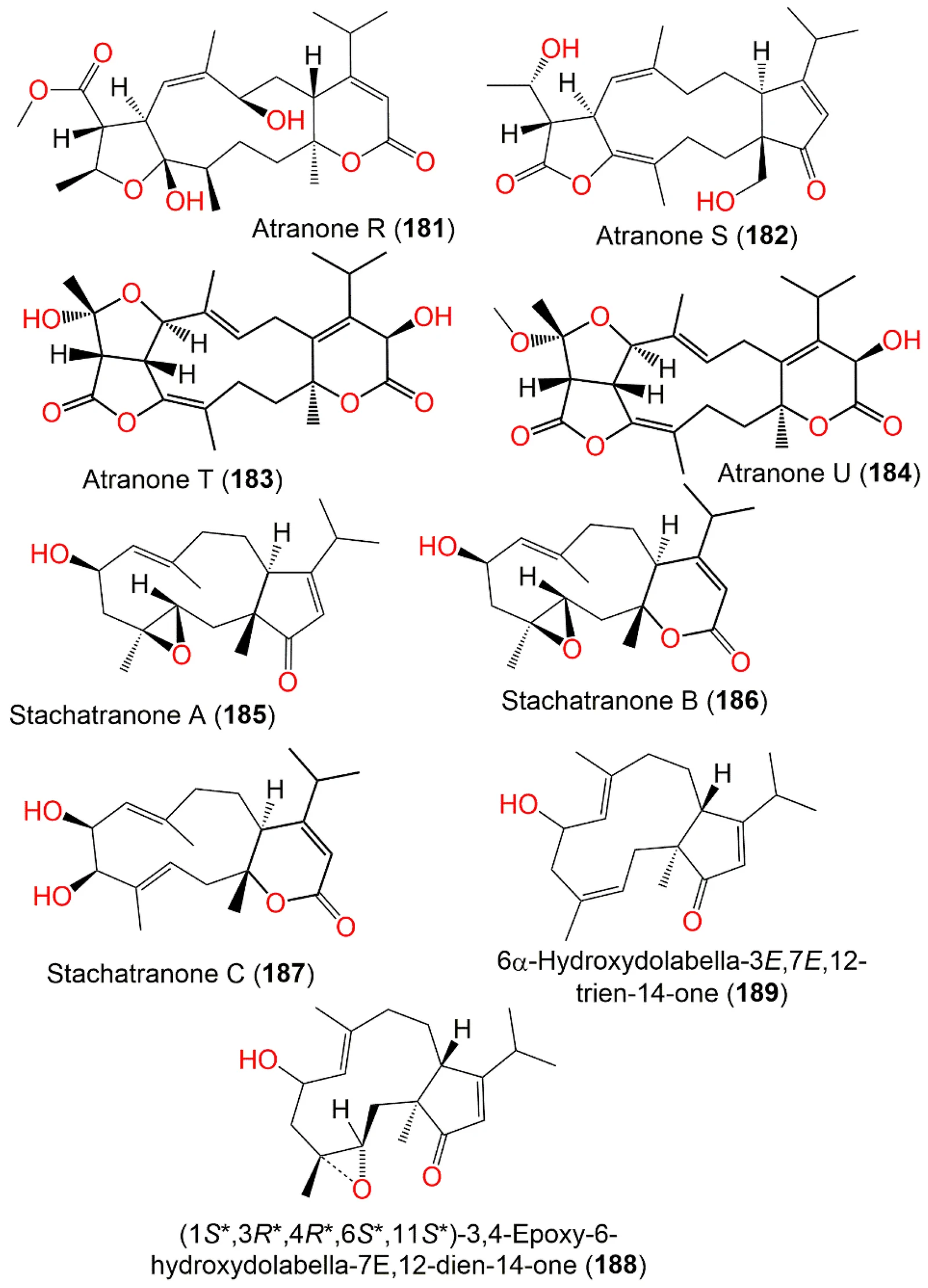
Structures of atranones (**181**–**184**) and dolabellanes (**185**–**190**) reported from *S. chartarum*.

In 2019, Yang et al. purified new atranones Q-S (**180**–**182**) and new dolabellane diterpenoids: stachatranone A–C (**185**–**187**) from the EtOAc extract of a marine-derived *S. chartarum* by SiO2 CC and RP-HPLC that were characterized by extensive spectral and X-ray analyses. Structurally, **186** and **187** possess 1,14-seco-dolabellane diterpenoid skeleton, whereas **180** featured C23 atranone having propan-2-one moiety connected via C-C bond to a dolabellane diterpenoid and **181** is C_24_ atranone with a 2-methyltetrahydrofuran-3-carboxylate unit linked at C5–C6 to dolabellane diterpenoid. Compound **186** had antimicrobial potential versus *E. faecalis* and *A. baumannii* (MIC 32 and 16 μg/mL, respectively), whereas **180** possessed noticeable inhibition versus MRSA, *C. albicans*, and *E. faecalis* (MICs 32, 8, and 16 μg/mL, respectively). Treatment of *C. albicans* with **180 **(8 μg/mL) induced obvious agglutination in cytoplasm and thinning, deformity, wrinkling, and irregularity of the cell wall, whereas at 16 μg/mL it resulted in the appearance of cytoplasmic vacuoles, deformation of the cell membrane and wall, and complete or partial leakage of the cell contents in TEM (transmission electron microscopy). This revealed that **180** had a destructive potential on *C. albicans* cell wall and cell membrane [61] (Figure 23).

**Scheme 10:**
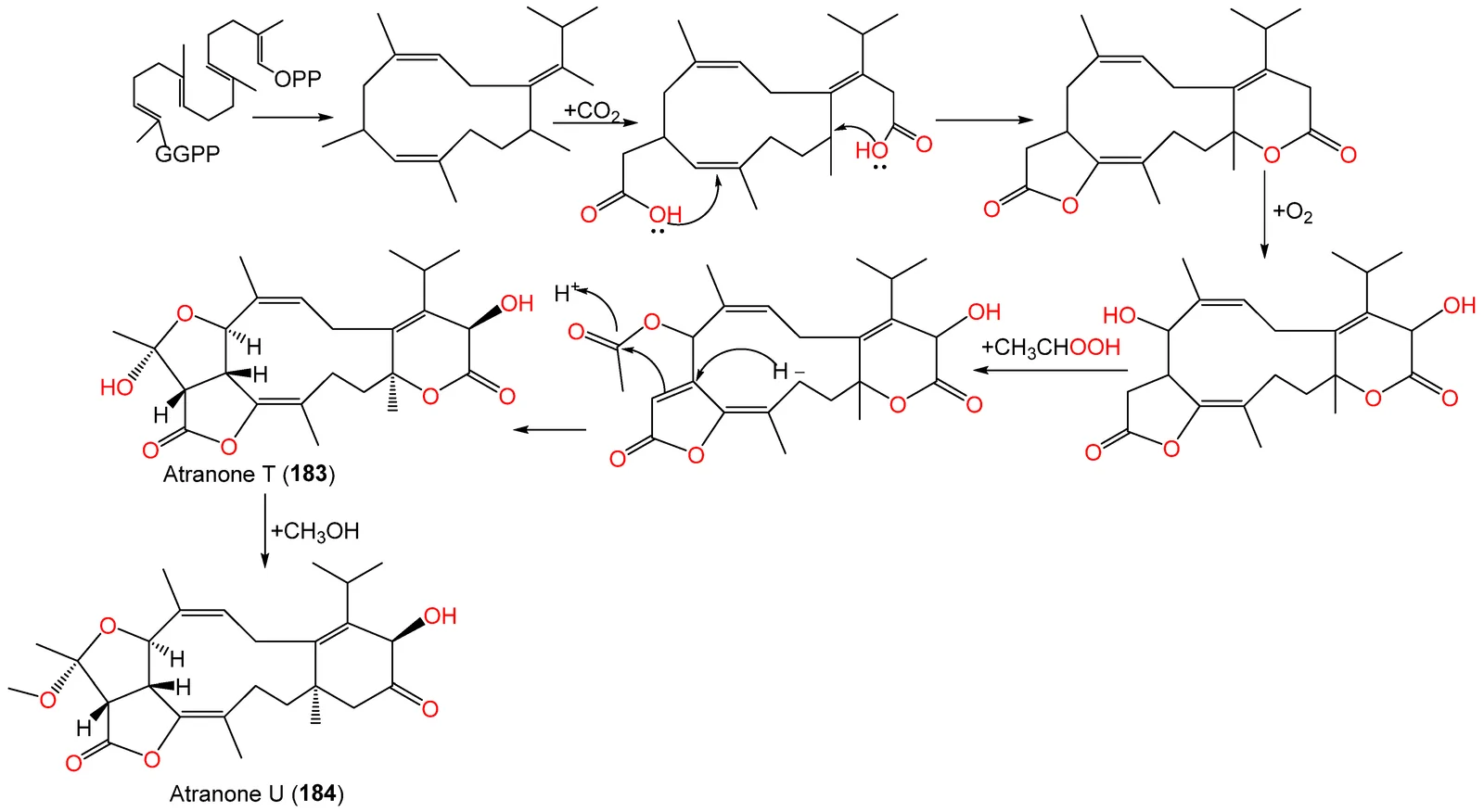
Biosynthetic pathway of atranones T and U (**183** and **184**) [62].

Two new atranones, T (**183**) and U (**184**), and three formerly reported analogs, **162**, **180**, and **187,** were purified from the broth and mycelia EtOAc extract of *S. chartarum* by SiO_2_ CC and RP-HPLC. They were structurally verified by spectroscopic and calculated ECD analyses. Compound **183** is atranone C (**165**) C-13-hydroxy analog with 1S/6R/7R/13R/21S/22R configuration, however, **184** is a C22 methoxylated derivative of **183**. It was proposed that these metabolites are biosynthesized starting with GGPP (geranylgeranyl pyrophosphate), as shown in Scheme 10. The cytotoxic evaluation versus MG-63 (human osteosarcoma cells) revealed that **180** (IC_50_ 8.6 μM) possessed promising cytotoxic capacity than 5-FU (5-fluorouracil, IC_50_ 10.4 μM) in the MTT. Further, **180** efficiently promoted MG-63 apoptosis, accompanied with G0/G1 cell cycle arrest. Thus, this compound could be further developed to a promising antitumor agent [62].

### 3.5. Chromenes

**Figure 24:**
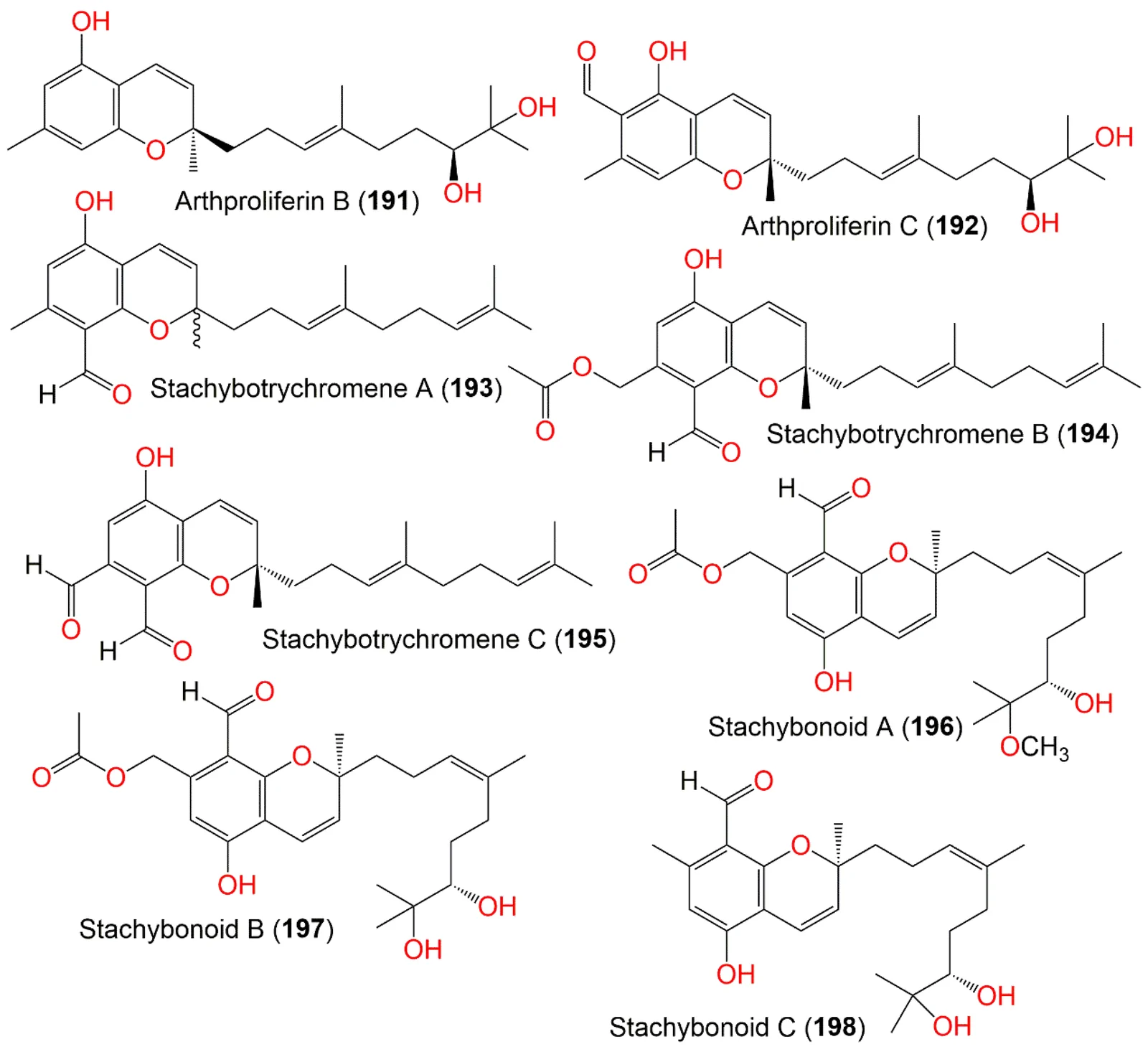
Structures of chromene derivatives (**191**–**198**) reported from *S. chartarum*.

Three novel meroterpenoids having a chromene moiety were joined with an isoprenoid chain; stachybotrychromenes A–C (**193**–**195**) were separated using SiO_2_ and RP-HPLC UV. Their cytotoxic potential was assessed versus HepG2 in the Alamar Blue assay. Compound **194** (IC_50_ 28.2 μM) had a more potent reduction in the cell survival than **193** (IC_50_ 73.7 μM), however, **195** was inactive [64]. It is noteworthy that **195** was assumed to be generated via the intermediates **193** and **194** (Figure 24).

The new chromene metabolites, stachybonoids A–C (**196**–**198**), were purified using HPLC and SiO_2_ and Sephadex LH-20 CC and characterized based on spectral, ECD, and X-ray analyses as well as Mosher’s method [68]. They were assessed for anti-dengue virus potential in luciferase assay using 293T cells. Compound **196** lessened the prM dengue virus protein expression. It is noteworthy that **196** inhibited the replication of dengue virus, whereas **197** increased the formation of dengue protein, when the only difference between their structures was whether 17-OH was methylated or not. 

**Scheme 11:**
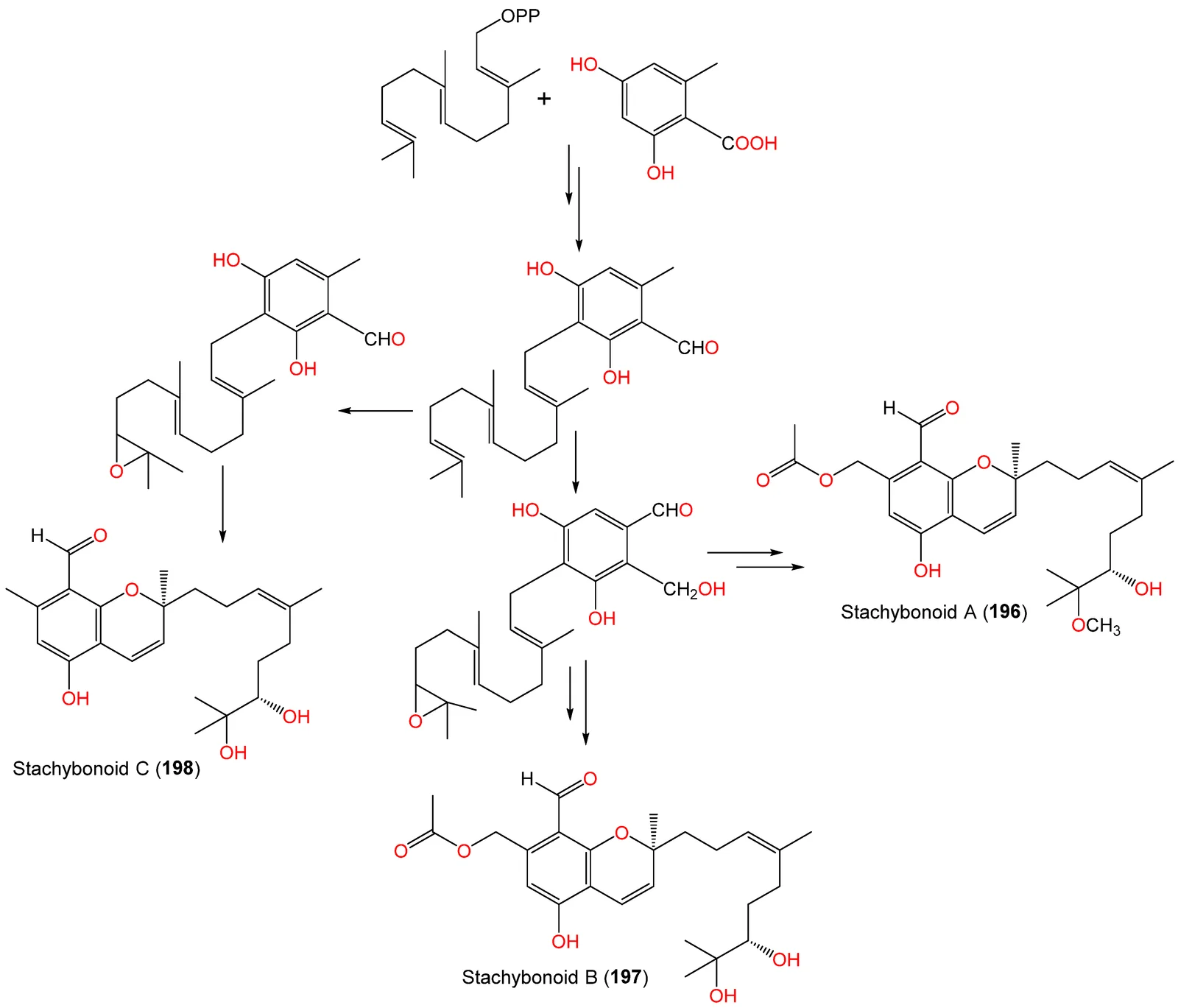
Biosynthetic pathway of stachybonoids A–C (**196**–**198**) [68].

It was proposed that they are generated from the addition reaction of farnesyl diphosphate and orsellinic acid to produce an intermediate ilicicolin B (Scheme 11) [68]). After that, the ilicicolin B prenyl group terminal olefinic bond is epoxidized to produce an intermediate. Subsequently, the linkage of the aromatic OH group to C-3 of the prenyl group occurs through electrophilic addition forms **196**–**198** [68]. 

*Sinularia* sp.-associated *S. chartarum* SCSIO4-1201 EtOAc extract yielded undescribed polyketide derivatives, arthproliferins B and C (**191** and **192**), that were separated using RP18 CC and HPLC. They were characterized through X-ray, spectroscopic, and ECD analyses. Compound **191** was 9R-configured and possessed structural similarity to daurichromenic acid previously reported from *Rhododendron dauricum* [123], except that the C16-C-17 double bond was dehydroxylated, whereas **192** was like **191** but it had an aldehydic group at C-2 and 9S configuration. Compounds **192** had no cytotoxic and antimicrobial potential [73].

### 3.6. Cochlioquinones

Cochlioquinones are a class of phytotoxic metabolites that have been firstly purified from the plant pathogenic fungi belonging to *Cochliobolus* genus [124]. They are meroterpenoids having quinone/or quinol moiety and displayed various bioactivities [125].

Chemokines, small signaling proteins, are secreted by various cells such as innate lymphocytes, stem, B, T, myeloid, dendritic, and stromal cells that are involved in diverse biological processes (e.g. leukocyte migration, chemotaxis, and inflammation) [126]. They are substantial for destructive or protective immune and inflammatory operations, as well as for the immune system development and homeostasis [126]. Moreover, they are linked with various illnesses such as viral infections, cancer, and autoimmune and inflammatory diseases [127]. The chemokine-induced pathway activation needs the selective chemokines binding to their target cell surface receptors. Chemokines and their receptors are found to be substantial targets by different antagonists for treating a variety of illnesses [128].

The quinoid derivatives, 11-*O*-methyl-epi-cochlioquinone A (**200**) and *epi*-cochlioquinone A (**199**), were purified from hexane fraction utilizing Rp-HPLC. Compound **200** was an 11-O-methyl derivative of **199**. Their C-11 α-configuration was deduced based on *J*_9α-11_ (9 Hz). These metabolites effectively competed with MIP-1α (macrophage inflammatory protein-1) for binding to human CCR5 (chemokine C-C motif-receptor 5) (IC_50_ 7.0 and 4.0 μM, respectively) [109].

### 3.7. Xanthones

**Figure 25:**
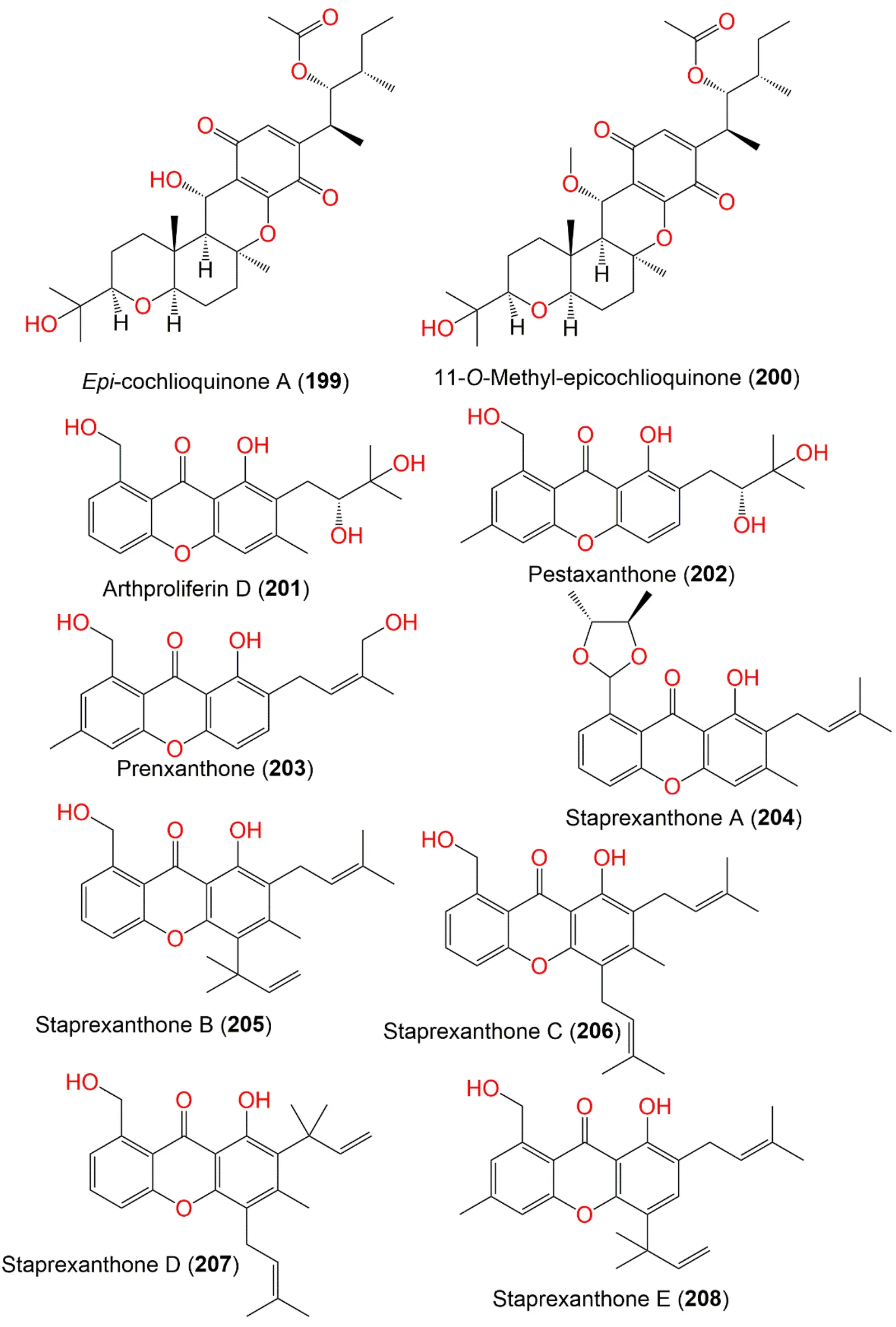
Structures of cochlioquinones (**199** and **200**) and xanthones (**201**–**208**) reported from *S. chartarum*.

A new xanthone arthproliferin D (**201**) and formerly reported pestaxanthone (**202**) and prenxanthone (**203**) were biosynthesized by *Sinularia* sp.-associated *S. chartarum* SCSIO4-1201. Compound **201** was like **202** but differs in the position of CH_3_ groups at C-4 and C-12 and its 16R-configuration was established by the CD spectrum (Figure 25). However, **202** possessed weak effectiveness (MIC 125 µg/mL) towards methicillin-resistant *S. aureus* ATCC-29213, compared to ampicillin (MIC 10.0 µg/mL). All had no cytotoxic capacity versus MDA-MB-231, C4-2B, MGC803, MDA-MB-468, and A549 cell lines in the CCK-4 assay [73].

Gan et al. reported the separation of new prenylxanthones, staprexanthones A–E (**204**–**208**) using SiO_2_ CC and RP-HPLC from the mycelia EtOAc extract of the mangrove-harbored *S. chartarum* HDN16-358. Their structures were secured by NMR and ECD analyses. Compound **204** features a C-8-linked 4,5-dimethyl-1,3-dioxolane moiety with 13R,14R-configuration, whereas **205**–**208** are rare mono-oxygenated prenylated xanthones. Compounds **204**, **205**, and **208** remarkably increased the number of β-cells in the zebrafish model in vivo. Further, **205** and **208** boosted the mass expansion of β-cells by increasing the existing β-cells proliferation via promoting cell-cycle progression at the G1/S phase, where **205** (Conc. 25 μM) was the most active stimulator with a 10% increased existing cell proliferation/day. This indicated the potential of these metabolites as drug leads for anti-diabetes agents through stimulating regeneration of β-cells [110].

### 3.8. Cyclosporins

Cyclosporins as immune-suppressant agents were established to be efficient not only in suppressing the transplanted-organ rejection but also in treating various autoimmune disorders that do not respond to current therapy [129]. 

**Figure 26:**
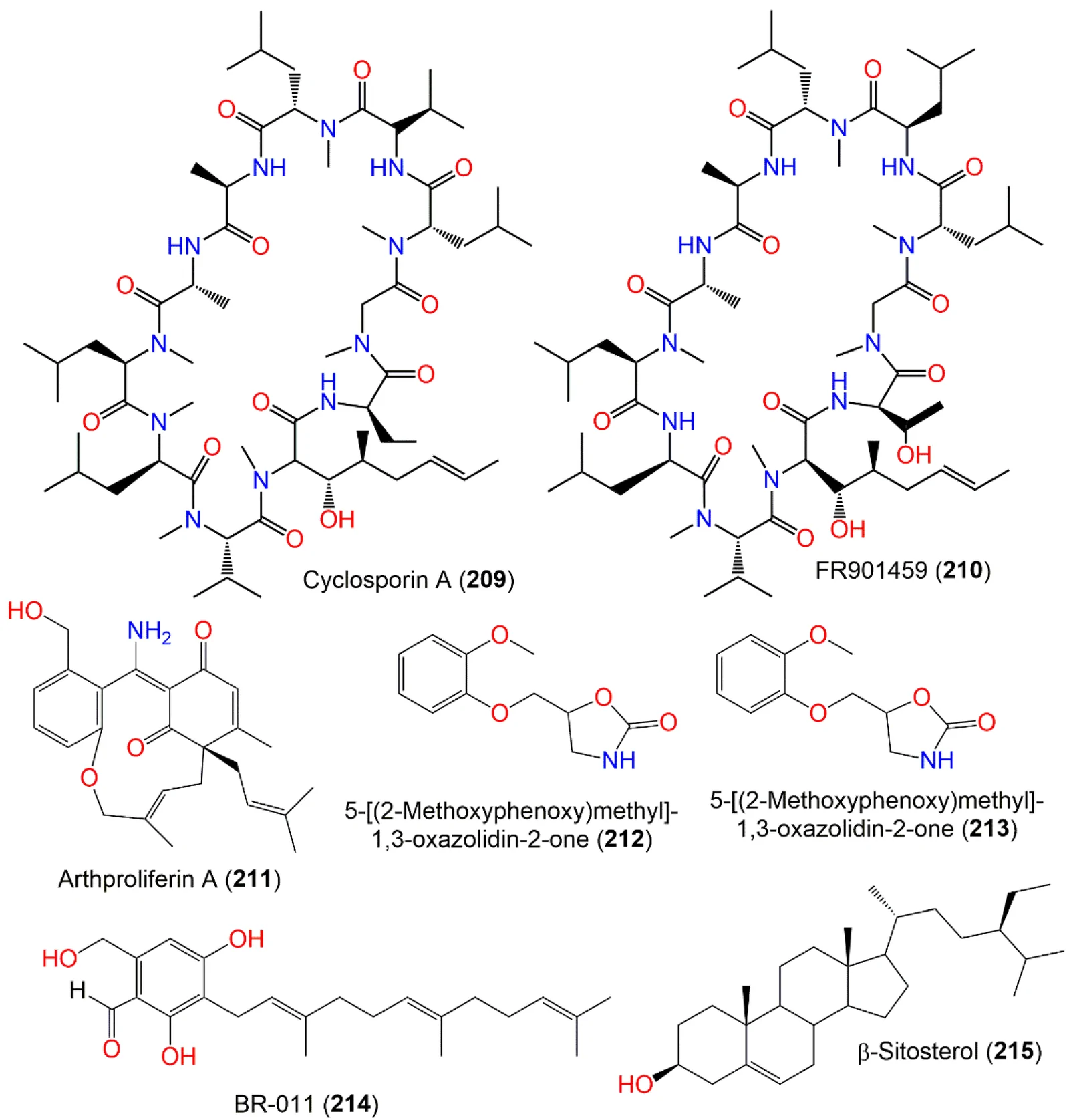
Structures of cyclosporins (**209** and **210**), nitrogenous compounds (**211**–**213**), phenolic (**214**), and sterol (**215**) reported from *S. chartarum*.

FR901459 (**210**), a novel immunosuppressant, had been purified along with cyclosporin A (CsA, **209**) from *S. chartarum* No.19392 fermentation by Diaion HP-20, activated carbon, and SiO_2_ CC. It gave positive results with I_2_ ceric SO_4_, KMnO_4_, and Dragendorff‘s reagents and negative ninhydrin (Figure 26). Its structure varied from other cyclosporins in having Leu at position 5 instead of Val relied on various spectral tools [111]. FR901459 possessed lymphocyte proliferation inhibitory effectiveness (IC_50_ 26.8 ng/mL) that was one-third the effectiveness of CsA (IC_50_ 9.9 ng/mL). It repressed IL-2 production as well as the ConA-produced mitogenic responses (IC_50_ 50.1 ng/mL) compared to CsA (IC_50_ 21.9 ng/mL). FR901459 (doses 100 and 320 mg/kg, oral) and CsA (doses 32 and 100 mg/kg, oral) prolonged skin allograft survival in rats. Therefore, it had one-third of the CsA potency in the in vitro and in vivo immune-suppression assays [111].

### 3.9. Other metabolites 

Arthproliferin A (**211**) is an undescribed polyketide derivative separated from *Sinularia* sp.-associated *S. chartarum* SCSIO4-1201. It was found to be like coleophomone B formerly reported from *Coleophoma* sp. [130], except it has C-1 oxymethylene instead of ketone group. Compounds **211** demonstrated moderate influence (MIC 78 µg/mL) versus methicillin-resistant *S. aureus* [73].

Korpi et al. reported that TSGC (thermal desorption-gas chromatography) and HPLC analyses of air samples of *S*. *chartarum* obtained from incubation chambers revealed the existence of 1-hexanol, 2-methy-1-propanol, formaldehyde, 3-octanone, 3-methy-1-butanol, acrolein, 1-octanol, 3-methylanisole, and geosmin [131].

In addition, trichodiene, a volatile sesquiterpene that is structurally related to trichothecene mycotoxins, was specified by GCMS in the *S. chartarum* headspace [132]. Further, MVOCs (microbial volatile organic compounds) of three *S. chartarum* strains obtained from water damaged homes by GCMS analysis included alcohols (e.g., 2-ethyl-1-hexanol, 2-butanol, 2-methyl-3-buten-2-ol, 2-methyl-1-butanol, 3-methyl-3-buten-1-ol, 2-ethyl-1-hexanol, and 2-propanol), ketones (e.g., 2-butanone, 2,2-dimethyl-3-pentanone, cyclopentanone, 3-cyclohepten-1-one, and 2-(1-cyclopent-1-enyl-1-methylethyl) cyclopentanone, and terpenes (e.g., α-farnesene, 7-dimethyl-1,3,6-octatriene, β-himachalene, β-cedrene, β-pinene, β-myrcene, β-bisabolene, terpinolene, and limonene) [133].

## 4. Extract Bioactivities and Nanoparticles

*S. chartarum* spores MeOH extract had cytotoxic effectiveness, prohibited proliferation, and induced cell death towards MH-S (murine alveolar macrophage cell line) through induction of DNA damage and p53 activation [134]. FIP-sch3, a FIP (fungal immunomodulatory protein), was identified from *S. chartarum* and expressed in *E. coli*. rFIP-shc3 (recombinant FIP-sch3) exhibited a potent anti-tumor potential versus MCF-7, H520, A549, HeLa, and HepG2 but had no effect in 293 (normal human embryonic kidney) cells. It significantly inhibited cell migration and proliferation via the induced apoptosis in A549 cells [135]. Li et al. stated that the solid rice culture EtOAc extract of *S. chartarum*, harboring *Niphates recondite* sponge, displayed noticeable cytotoxic capacity (IC_50_ <10 mg/mL) versus a panel of tumor cell lines (e.g., BGC-823, HCT-116 NCI-H1650, A2780, and HepG2) [92]. The fermentation broth EtOAc extract of the fungal species isolated from *Himerometra magnipinna* revealed moderate anti-inflammation capacity (IC_50_ 36 µM) by suppressing LPS-produced NO production in RAW264.7 cells [108].

Recently, nanotechnology has become one of the emerging research areas for developing a variety of nanomaterials [1,3]. It is considered an economical alternative for physical and chemical methods for nanoparticles’ (NPs) synthesis. The ordinary methods for NPs’ synthesis are non-eco-friendly, costly, and toxic, which necessitates the need for clean, reliable, safe, and eco-friendly methods [136]. The microorganism’s utilization in the NPs’ synthesis emerges as an exciting and eco-friendly approach. Different kinds of NPs have been synthesized using various fungal species [2,3,5,39]. Fungi are advantageous over bacteria in NPs’ preparation because of easy handling, simple nutrient requirement, great wall-binding potential, and metal intracellular uptake capacities [137]. Mohamed synthesized *S. chartarum* silver NPs (AgNPs) using AgNO_3_ that possessed more potent potential versus bacteria than fungi [138]. 

## 5. Conclusions

**Figure 27:**
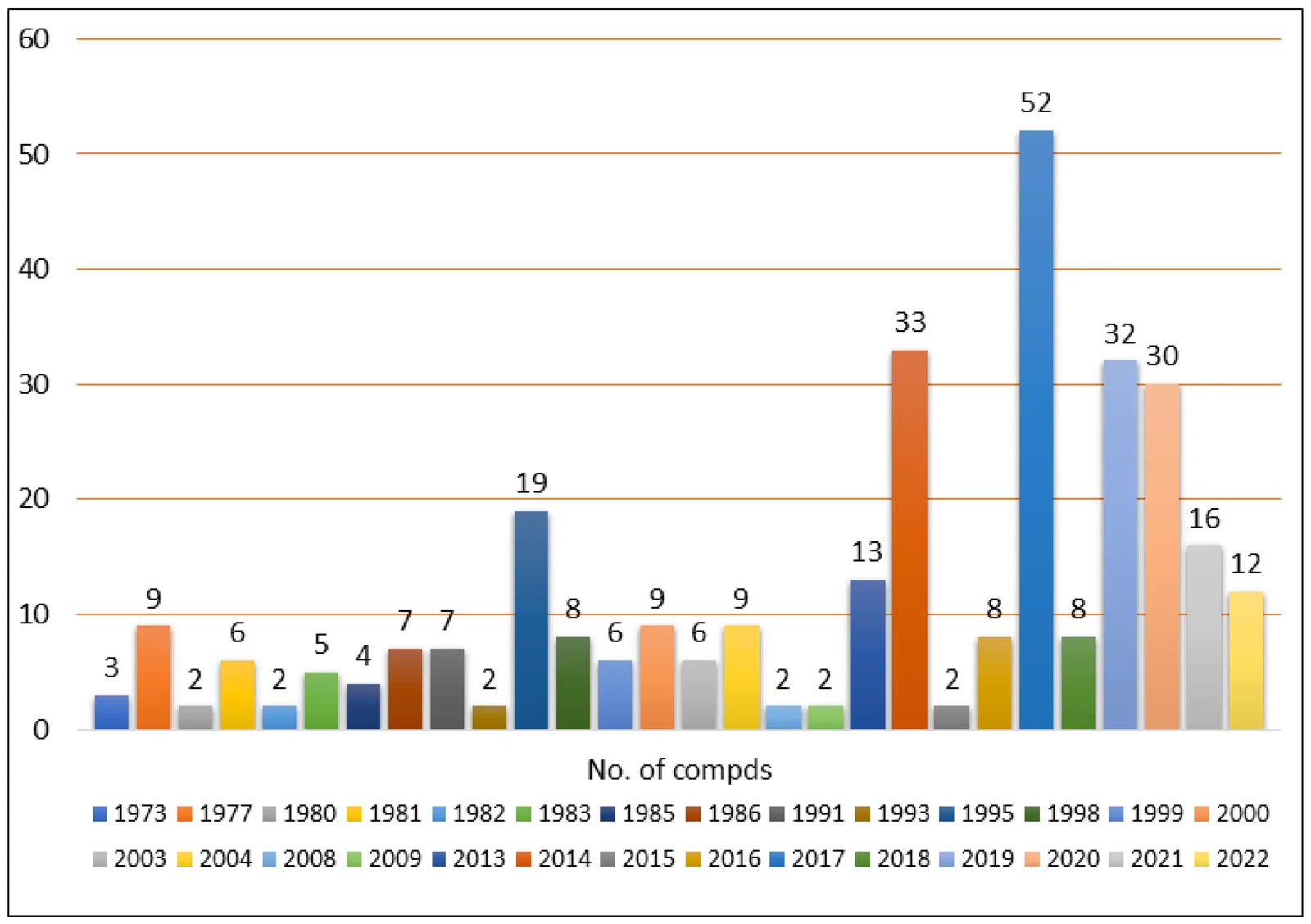
Number of metabolites per year reported from *S. chartarum*.

**Figure 28:**
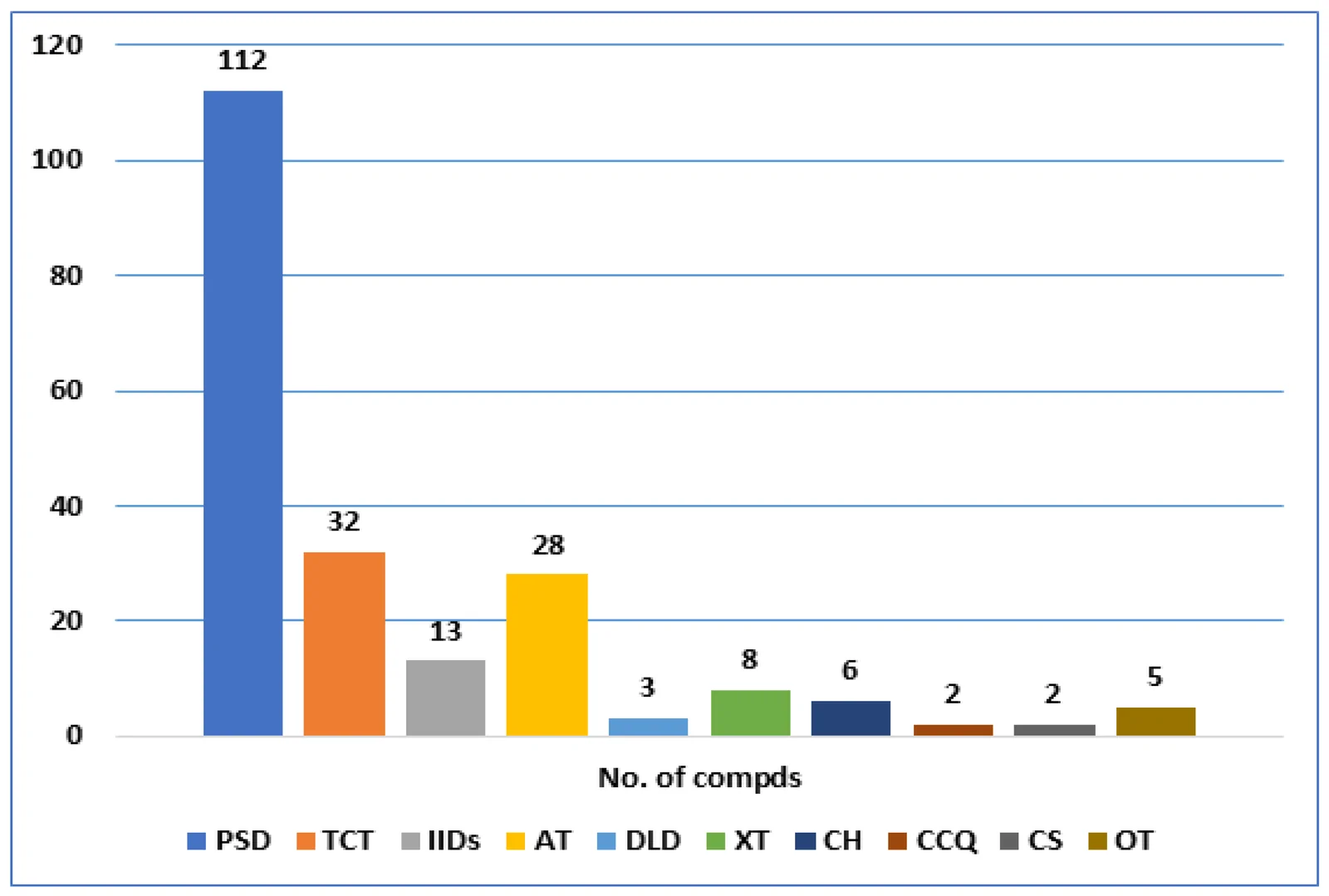
Different classes of metabolites reported from *S. chartarum.* PSD: Phenylspirodrimanes: TCT: Trichothecenes; IIDs: Isoindoline derivatives; ATs: Atranones; DLDs: Dolabellane diterpenoids; XTs: Xanthones; CHs: Chromenes; CCQ: Cochlioquinones; CS: Cyclosporins; OT: Others.

Fungi represent huge reservoirs of structural varied secondary metabolites and biotechnologically useful enzymes. *S. chartarum* is a toxigenic fungus species that is separated from water-damaged buildings as well as plants, soil, and marine sponges and is known to have life-threatening health impacts on humans and animals. From this fungus, 215 metabolites with diverse and unique structural features have been separated from the period 1973 through 2022 (Figure 27), including phenylspirodrimanes (112 compounds), trichothecenes (32 compounds), atranones (28 compounds), and isoindoline derivatives (13 compounds) as major metabolites (Figure 28).

**Figure 29:**
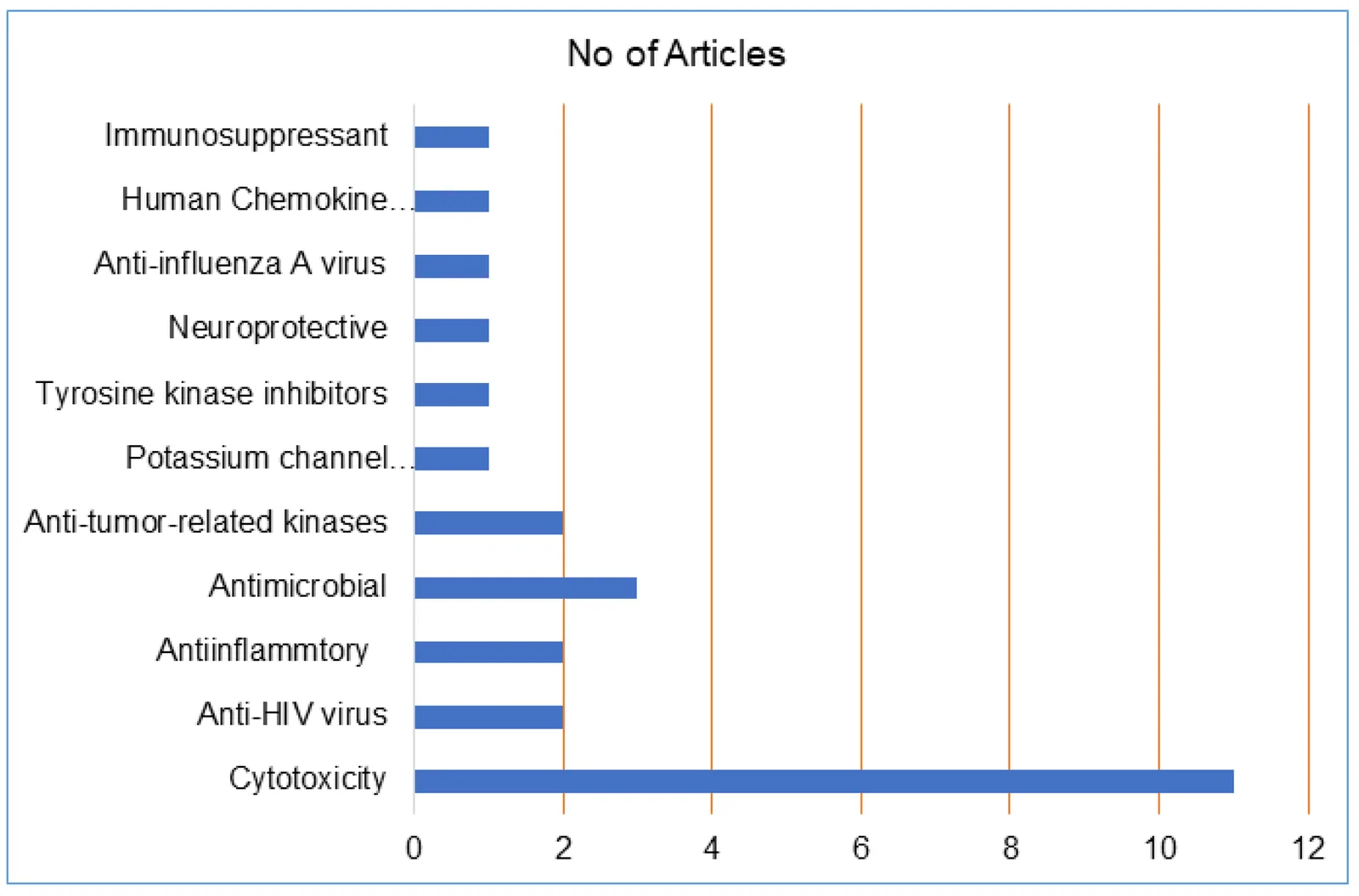
Biological activities of reported metabolites and the number of articles.

These metabolites have been evaluated for various bioactivities such as cytotoxicity, anti-HIV virus, anti-inflammatory, antimicrobial, potassium channel inhibition, tyrosine kinase, and tumor-related kinase inhibitory, neuroprotective, anti-influenza A virus, human chemokine antagonist, and immunosuppressant characteristics (Figure 29). The unique scaffold of trichothecenes sesquiterpenoids may be considered one of the characteristics of this fungus. Additionally, some trichothecenes and atranones could be promising leads for the development of antitumor agents. 

Stachybonoid A (**196**) exhibited anti-dengue potential; therefore, it could be a promising metabolite for developing dengue virus inhibitory agents. Staprexanthones B (**205**) and E (**205**) markedly stimulated the regeneration of β-cells; hence, they have the potential as drug leads for anti-diabetes agents. It is noteworthy that the structural variation among these metabolites directly influences their bioactivities. It is noteworthy that this fungus possesses potential and multiple enzymatic systems that might contribute to the diversity of its metabolites. Nevertheless, the enzymes and genes accountable for these unique metabolites’ biosynthesis are a fruitful area of future research. 

Further, this fungus has an amazing capacity to produce diverse enzymes that could be beneficial for biotechnological and industrial applications. In addition, they could represent an eco-friendly biodegradation tool that can exchange dangerous wastes into advantageous products. It was found that certain enzymes modification could boost their activity, revealing a new area of future research in this fungus’ enzymes. 

There is an only one report on the synthesis of NPs using this fungus. Thus, further studies to synthesize various types of NPs from *S. chartarum* and possible applications of these synthesized NPs can be carried out.

An obvious theme that could be garnered from this work is that, although there is remarkable progress towards the characterization of *S. chartarum* metabolites, we think that there remain many hidden metabolites that need to be discovered. Moreover, further biological evaluations of the reported metabolites and their biosynthetic pathway studies are required. Additionally, the limited in vivo and mechanistic studies of these metabolites necessitate the research focus in this direction. Many of the reported metabolites either are not tested or possessed no observed effectiveness in some of the evaluated activities, therefore, an in silico screening for the potential bioactivities, as well as their derivatization, should clearly be the goal of future research. 

Finally, we believe that the therapeutic potential and chemical diversity of this fungus’ metabolites, with more in-depth research, will provide medicinal chemists and biologists with a more promising sustainable treasure-trove for drug discovery. 

## Figures and Tables

**Table 1:** List of compounds isolated from *Stachybotrys chartarum*.

Compound Name	Mol. Wt.	Mol. Formula	Host (Part, Family)	Place	Ref.
Stachybotrydial (**1**)	386	C_23_H_30_O_5_	Cultured	-	[65]
K-76 (**2**)	402	C_23_H_30_O_6_	Cultured	-	[65]
Mer-NF5003E (**3**)	388	C_23_H_32_O_5_	*Xestospongia testudinaris* (Sponge, Petrosiidae)	Xisha Island, China	[66]
	-	-	Cultured	-	[67]
Stachybotrysin B (**4**)	430	C_25_H_34_O_6_	Cultured	-	[67]
Mer-NF5003B (**5**)	404	C_23_H_32_O_6_	*Xestospongia testudinaris* (Sponge, Petrosiidae)	Xisha Island, China	[66]
Stachybotrysin C (**6**)	446	C_25_H_34_O_7_	*Himerometra magnipinna* (Crinoids, Himerometridae)	Zhanjiang Mangrove National Nature Reserve, Guangdong, China	[68]
	-	-	*Himerometra magnipinna* (Crinoids, Himerometridae)	-	[67]
Stachybonoid D (**7**)	488	C_27_H_36_O_8_	*Himerometra magnipinna* (Crinoids, Himerometridae)	Zhanjiang Mangrove National Nature Reserve, Guangdong, China	[68]
Stachyboside A (**8**)	550	C_29_H_42_O_10_	*Xestospongia testudinaris* (Sponge, Petrosiidae)	Xisha Island, China	[66]
Stachyboside B (**9**)	566	C_29_H_42_O_11_	*Xestospongia testudinaris* (Sponge, Petrosiidae)	Xisha Island, China	[66]
Stachybotrysin D (**10**)	468	C_23_H_32_O_8_S	Cultured	-	[67]
Stachybotrysin E (**11**)	428	C_25_H_32_O_6_	Cultured	-	[67]
F1839-I (**12**)	372	C_23_H_32_O_4_	*Xestospongia testudinaris* (Sponge, Petrosiidae)	Xisha Island, China	[66]
Stachybotrysin A (**13**)	414	C_25_H_34_O_5_	Cultured	-	[67]
Stachartin A = Stachybotrysin (**14**)	428	C_26_H_36_O_5_	Cultured	-	[67]
	-	-	Soil sample	Datun tin mine tailings area, Yunnan, China	[69]
Stachybochartin G (**15**)	388	C_23_H_32_O_5_	*Pinellia ternata* (rhizomes, Araceae)	Nanjing, Jiangsu, China	[70]
Stachybotrysin F (**16**)	428	C_26_H_36_O_5_	Cultured	-	[67]
Stachybotrysin G (**17**)	428	C_26_H_36_O_5_	Cultured	-	[67]
Stachybotrysin H (**18**)	444	C_26_H_36_O_6_	Cultured	-	[71]
Stachybotrysin I (**19**)	444	C_26_H_36_O_6_	Cultured	-	[71]
Stachybotrylactone = Stachybotrolide (**20**)	386	C_23_H_30_O_5_	Cultured	-	[65]
	-	-	Mud	East Dongting Lake, Hunan, China	[72]
	-	-	*Sinularia* sp. (Soft coral, Alcyoniidae)	Yongxing Island, South Chian Sea, China	[73]
	-	-	Soil sample	Datun tin mine tailings area, Yunnan, China	[69]
	-	-	*Himerometra magnipinna* (Crinoids, Himerometridae)	Zhanjiang Mangrove National Nature Reserve, Guangdong, China	[68]
Stachybotrylactone acetate (**21**)	428	C_25_H_32_O_6_	Cultured	-	[65,67]
	-	-	Mud	East Dongting Lake, Hunan, China	[72]
Stachybotrane A (**22**)	386	C_23_H_30_O_5_	Mud	East Dongting Lake, Hunan, China	[72]
	-	-	*Pinellia ternata* (rhizomes, Araceae)	Nanjing, Jiangsu, China	[70]
Stachybotrane B (**23**)	428	C_25_H_32_O_6_	Mud	East Dongting Lake, Hunan, China	[72]
	-	-	*Pinellia ternata* (rhizomes, Araceae)	Nanjing, Jiangsu, China	[70]
2α-Hydroxystachybotrylactone (**24**)	402	C_23_H_30_O_6_	Cultured	-	[65]
	-	-	Mud	East Dongting Lake, Hunan, China	[72]
Stachybochartin E (**25**)	444	C_25_H_32_O_7_	*Pinellia ternata* (rhizomes, Araceae)	Nanjing, Jiangsu, China	[70]
	-	-	Mud	East Dongting Lake, Hunan, China	[72]
2α-Acetoxystachybotrylactone acetate (**26**)	486	C_27_H_34_O_8_	Cultured	-	[65]
Stachybotrane C (**27**)	402	C_23_H_30_O_6_	Mud	East Dongting Lake, Hunan, China	[72]
Stachybotryslactone B = Stachartin B (**28**)	386	C_23_H_30_O_5_	Cultured	-	[67]
	-	-	Soil sample	Datun tin mine tailings area, Yunnan, China	[69]
	-	-	*Himerometra magnipinna* (Crinoids, Himerometridae)	Zhanjiang Mangrove National Nature Reserve, Guangdong, China	[68]
Stachybotrylactam (**29**)	385	C_23_H_31_NO_4_	Cultured	-	[65]
	-	-	*Niphates recondita* (Sponge, Niphatidae)	Coral reef near Weizhou Island, Beibuwan Bay, Guangxi, China	[74]
	-	-	Soil sample	Datun tin mine tailings area, Yunnan, China	[69]
	-	-	Cultured	-	[71]
	-	-	*Pinellia ternata *(rhizomes, Araceae)	Nanjing, Jiangsu, China	[70]
Stachybotrylactam acetate (**30**)	427	C_25_H_33_NO_5_	Cultured	-	[65]
	-	-	*Niphates recondita* (Sponge, Niphatidae)	Coral reef near Weizhou Island, Beibuwan Bay, Guangxi, China	[74]
	-	-	*Sinularia* sp. (Soft coralm Alcyoniidae)	Yongxing Island, South Chian Sea, China	[73]
Arthproliferin E = Stachybotrin E (**31**)	399	C_24_H_33_NO_4_	*Sinularia* sp. ( Soft coral, Alcyoniidae)	Yongxing Island, South Chian Sea, China	[73]
	-	-	Cultured	-	[71]
Stachybotrin = Stachybotramide (**32**)	429	C_25_H_35_NO_5_	*Sinularia* sp. (Soft coral, Alcyoniidae)	Yongxing Island, South Chian Sea, China	[73]
	-	-	*Pinellia ternata* (rhizomes, Araceae)	Nanjing, Jiangsu, China	[70]
	-	-	Cultured	-	[65,75]
	-	-	*Niphates recondita* (Sponge, Niphatidae)	Coral reef near Weizhou Island, Beibuwan Bay, Guangxi, China	[74]
	-	-	Mud	East Dongting Lake, Hunan, China	[72]
Stachybochartin F (**33**)	471	C_27_H_37_NO_6_	*Pinellia ternata* (rhizomes, Araceae)	Nanjing, Jiangsu, China	[70]
Stachybotrin D (**34**)	441	C_26_H_35_NO_5_	*Xestospongia testudinaris* (Sponge, Petrosiidae)	Xisha Island, China	[66]
Stachybotrysam E (**35**)	471	C_27_H_37_NO_6_	Cultured	-	[76]
K-76-1 (**36**)	515	C_28_H_37_NO_8_	Soil	Himalaya, India	[77]
	-	-	*Xestospongia testudinaris* (Sponge, Petrosiidae)	Xisha Island, China	[66]
K-76-2 (**37**)	487	C_27_H_37_NO_7_	Soil	Himalaya, India	[77]
Stachybotrin E (**38**)	529	C_29_H_39_NO_8_	*Xestospongia testudinaris* (Sponge, Petrosiidae)	Xisha Island, China	[66]
Stachybotrin F (**39**)	529	C_29_H_39_NO_8_	*Xestospongia testudinaris* (Sponge, Petrosiidae)	Xisha Island, China	[66]
Chartarlactam A (**40**)	399	C_23_H_29_NO_5_	*Niphates recondita* (Sponge, Niphatidae)	Coral reef near Weizhou Island, Beibuwan Bay, Guangxi, China	[74]
	-	-	Soil sample	Datun tin mine tailings area, Yunnan, China	[69]
Chartarlactam B (**41**)	485	C_28_H_39_NO_6_	*Niphates recondita* (Sponge, Niphatidae)	Coral reef near Weizhou Island, Beibuwan Bay, Guangxi, China	[74]
Chartarlactam C (**42**)	401	C_23_H_31_NO_5_	*Niphates recondita* (Sponge, Niphatidae)	Coral reef near Weizhou Island, Beibuwan Bay, Guangxi, China	[74]
Chartarlactam D (**43**)	563	C_30_H_45_NO_9_	*Niphates recondita* (Sponge, Niphatidae)	Coral reef near Weizhou Island, Beibuwan Bay, Guangxi, China	[74]
Chartarlactam E (**44**)	383	C_23_H_29_NO_4_	*Niphates recondita* (Sponge, Niphatidae)	Coral reef near Weizhou Island, Beibuwan Bay, Guangxi, China	[74]
	-	-	*Sinularia* sp. (Soft coral, Alcyoniidae)	Yongxing Island, South Chian Sea, China	[73]
Stachartin C (**45**)	499	C_29_H_41_NO_6_	Soil sample	Datun tin mine tailings area, Yunnan, China	[69]
Stachartin D (**46**)	513	C_30_H_43_NO_6_	Soil sample	Datun tin mine tailings area, Yunnan, China	[69]
Stachybonoid E (**47**)	457	C_26_H_35_NO_6_	*Himerometra magnipinna* (Crinoids, Himerometridae)	Zhanjiang Mangrove National Nature Reserve, Guangdong, China	[68]
	-	-	Soil sample	Datun tin mine tailings area, Yunnan, China	[78]
Stachybonoid F (**48**)	485	C_28_H_39_NO_6_	*Himerometra magnipinna* (Crinoids, Himerometridae)	Zhanjiang Mangrove National Nature Reserve, Guangdong, China	[68]
	-	-	Soil sample	Datun tin mine tailings area, Yunnan, China	[78]
Stachartin E (**49**)	547	C_33_H_41_NO_6_	Soil sample	Datun tin mine tailings area, Yunnan, China	[69]
*N*-(2-Benzenepropanoic acid) stachybotrylactam (**50**)	533	C_32_H_39_NO_6_	*Niphates recondita* (Sponge, Niphatidae)	Coral reef near Weizhou Island, Beibuwan Bay, Guangxi, China	[74]
F1839-A (**51**)	401	C_23_H_31_NO_5_	*Niphates recondita* (Sponge, Niphatidae)	Coral reef near Weizhou Island, Beibuwan Bay, Guangxi, China	[74]
	-	-	*Sinularia* sp. (Soft coral, Alcyoniidae)	Yongxing Island, South Chian Sea, China	[73]
Chartarlactam I (**52**)	401	C_23_H_31_NO_5_	*Niphates recondita* (Sponge, Niphatidae)	Coral reef near Weizhou Island, Beibuwan Bay, Guangxi, China	[74]
Chartarlactam J (**53**)	401	C_23_H_31_NO_5_	*Niphates recondita* (Sponge, Niphatidae)	Coral reef near Weizhou Island, Beibuwan Bay, Guangxi, China	[74]
Chartarlactam K (**54**)	443	C_25_H_33_NO_6_	*Niphates recondita* (Sponge, Niphatidae)	Coral reef near Weizhou Island, Beibuwan Bay, Guangxi, China	[74]
Chartarlactam M (**55**)	385	C_23_H_31_NO_4_	*Niphates recondita* (Sponge, Niphatidae)	Coral reef near Weizhou Island, Beibuwan Bay, Guangxi, China	[74]
Chartarlactam P (**56**)	401	C_23_H_31_NO_5_	*Niphates recondita* (Sponge, Niphatidae)	Coral reef near Weizhou Island, Beibuwan Bay, Guangxi, China	[74]
F1839-E (**57**)	445	C_25_H_35_NO_6_	*Niphates recondita* (Sponge, Niphatidae)	Coral reef near Weizhou Island, Beibuwan Bay, Guangxi, China	[74]
	-	-	Mud	East Dongting Lake, Hunan, China	[72]
	-	-	Mud	East Dongting Lake, Hunan, China	[72]
Stachybotrane D (**58**)	445	C_25_H_35_NO_6_	Mud	East Dongting Lake, Hunan, China	[72]
K-76-3 (**59**)	487	C_27_H_37_NO_7_	Soil	Himalaya, India	[77]
K-76-4 (**60**)	501	C_28_H_39_NO_7_	Soil	Himalaya, India	[77]
K-76-5 (**61**)	515	C_29_H_41_NO_7_	Soil	Himalaya, India	[77]
K-76-6 (**62**)	515	C_29_H_41_NO_7_	Soil	Himalaya, India	[77]
K-76-7 (**63**)	549	C_32_H_39_NO_7_	Soil	Himalaya, India	[77]
2α-Acetoxystachybotrylactam acetate (**64**)	485	C_27_H_35_NO_7_	Cultured	-	[65]
	-	-	*Niphates recondita* (Sponge, Niphatidae)	Coral reef near Weizhou Island, Beibuwan Bay, Guangxi, China	[74]
	-	-	*Sinularia* sp. (Soft coral, Alcyoniidae)	Yongxing Island, South Chian Sea, China	[73]
Chartarlactam F (**65**)	385	C_23_H_31_NO_4_	*Niphates recondita* (Sponge, Niphatidae)	Coral reef near Weizhou Island, Beibuwan Bay, Guangxi, China	[74]
	-	-	*Sinularia* sp. (Soft coral, Alcyoniidae)	Yongxing Island, South Chian Sea, China	[73]
Chartarlactam G (**66**)	385	C_23_H_31_NO_4_	*Niphates recondita* (Sponge, Niphatidae)	Coral reef near Weizhou Island, Beibuwan Bay, Guangxi, China	[74]
Chartarlactam H (**67**)	429	C_25_H_35_NO_5_	*Niphates recondita* (Sponge, Niphatidae)	Coral reef near Weizhou Island, Beibuwan Bay, Guangxi, China	[74]
Chartarlactam N (**68**)	429	C_25_H_35_NO_5_	*Niphates recondita* (Sponge, Niphatidae)	Coral reef near Weizhou Island, Beibuwan Bay, Guangxi, China	[74]
Chartarlactam O (**69**)	385	C_23_H_31_NO_4_	*Niphates recondita* (Sponge, Niphatidae)	Coral reef near Weizhou Island, Beibuwan Bay, Guangxi, China	[74]
Stachartin F (**70**)	485	C_28_H_39_NO_6_	Soil sample	Datun tin mine tailings area, Yunnan, China	[78]
F1839-D (**71**)	401	C_23_H_31_NO_5_	*Niphates recondita* (Sponge, Niphatidae)	Coral reef near Weizhou Island, Beibuwan Bay, Guangxi, China	[74]
Chartarlactam L (**72**)	766	C_46_H_58_N_2_O_8_	*Niphates recondita* (Sponge, Niphatidae)	Coral reef near Weizhou Island, Beibuwan Bay, Guangxi, China	[74]
Bistachybotrysin A (**73**)	774	C_46_H_62_O_10_	Cultured	-	[79]
Bistachybotrysin B (**74**)	816	C_48_H_64_O_11_	Cultured	-	[79]
Bistachybotrysin C (**75**)	790	C_46_H_62_O_11_	Cultured	-	[79]
Bistachybotrysin D (**76**)	772	C_47_H_64_O_9_	Cultured	-	[80]
Bistachybotrysin E (**77**)	772	C_47_H_64_O_9_	Cultured	-	[80]
Bistachybotrysin F (**78**)	800	C_48_H_64_O_10_	Cultured	-	[81]
Bistachybotrysin G (**79**)	774	C_46_H_62_O_10_	Cultured	-	[81]
Bistachybotrysin H (**80**)	816	C_48_H_64_O_11_	Cultured	-	[81]
Bistachybotrysin I (**81**)	788	C_47_H_64_O_10_	Cultured	-	[81]
Bistachybotrysin J (**82**)	816	C_49_H_66_O_11_	Cultured	-	[81]
Bistachybotrysin K (**83**)	758	C_46_H_62_O_9_	Cultured	-	[82]
Bistachybotrysin L (**84**)	772	C_47_H_64_O_9_	Cultured	-	[83]
Bistachybotrysin M (**85**)	772	C_47_H_64_O_9_	Cultured	-	[83]
Bistachybotrysin N (**86**)	772	C_47_H_64_O_9_	Cultured	-	[83]
Bistachybotrysin O (**87**)	772	C_47_H_64_O_9_	Cultured	-	[83]
Bistachybotrysin P (**88**)	814	C_49_H_66_O_10_	Cultured	-	[83]
Bistachybotrysin Q (**89**)	814	C_49_H_66_O_10_	Cultured	-	[83]
Bistachybotrysin R (**90**)	854	C_52_H_70_O_10_	Cultured	-	[83]
Bistachybotrysin S (**91**)	854	C_52_H_70_O_10_	Cultured	-	[83]
Bistachybotrysin T (**92**)	798	C_49_H_66_O_9_	Cultured	-	[83]
Bistachybotrysin U (**93**)	756	C_46_H_60_O_9_	Cultured	-	[83]
Bistachybotrysin V (**94**)	758	C_46_H_62_O_9_	Cultured	-	[83]
Bistachybotrysin W (**95**)	832	C_48_H_64_O_12_	Cultured	-	[84]
Bistachybotrysin X (**96**)	832	C_48_H_64_O_12_	Cultured	-	[84]
Bistachybotrysin Y (**97**)	874	C_50_H_67_O_13_	Cultured	-	[84]
Chartarolide A (**98**)	772	C_44_H_49_O_10_Cl	*Niphates* sp. (Sponge, Niphatidae)	Near coral reef, Beibuwan Bay, GuangXi, China	[85]
Chartarolide B (**99**)	772	C_44_H_49_O_10_Cl	*Niphates* sp. (Sponge, Niphatidae)	Near coral reef, Beibuwan Bay, GuangXi, China	[85]
Chartarolide C (**100**)	771	C_44_H_50_NO_9_Cl	*Niphates* sp. (Sponge, Niphatidae)	Near coral reef, Beibuwan Bay, GuangXi, China	[85]
Chartarlactam Q (**101**)	753	C_46_H_59_NO_8_	Cultured	-	[86]
Chartarlactam R (**102**)	797	C_48_H_63_NO_9_	Cultured	-	[86]
Chartarlactam S (**103**)	771	C_46_H_61_NO_9_	Cultured	-	[86]
Chartarlactam T (**104**)	855	C_50_H_65_NO_11_	Cultured	-	[86]
Stachybochartin A (**105**)	774	C_46_H_62_O_10_	*Pinellia ternata* (Rhizomes, Araceae)	Nanjing, Jiangsu, China	[70]
Stachybochartin B (**106**)	844	C_50_H_68_O_11_	*Pinellia ternata* (Rhizomes, Araceae)	Nanjing, Jiangsu, China	[70]
Stachybochartin C (**107**)	772	C_47_H_64_O_9_	*Pinellia ternata* (Rhizomes, Araceae)	Nanjing, Jiangsu, China	[70]
Stachybochartin D (**108**)	858	C_50_H_66_O_12_	*Pinellia ternata* (Rhizomes, Araceae)	Nanjing, Jiangsu, China	[70]
Stachyin B (**109**)	755	C_46_H_61_NO_8_	Cultured	-	[86]
Stachartone A (**110**)	758	C_46_H_62_O_9_	Soil sample	Datun tin mine tailings area, Yunnan, China	[87]
Stachartarin A (**111**)	758	C_46_H_62_O_9_	Soil sample	Datun tin mine tailings area, Yunnan, China	[88]
Stachartarin B (**112**)	772	C_47_H_64_O_9_	Soil sample	Datun tin mine tailings area, Yunnan, China	[89]
Stachybocin E (**113**)	824	C_50_H_68_N_2_O_8_	*Xestospongia testudinaris* (Sponge, Petrosiidae)	Xisha Island, China	[66]
Stachybocin F (**114**)	838	C_51_H_70_N_2_O_8_	*Xestospongia testudinaris* (Sponge, Petrosiidae)	Xisha Island, China	[66]
12,13-Epoxytrichothec-9-ene (**115**)	234	C_15_H_22_O_2_	Cultured	-	[90,91]
2,4,12-Trihydroxyapotrichothecene (**116**)	268	C_15_H_24_O_4_	*Niphates recondita* (Sponge, Niphatidae)	Inner coral reef, Beibuwan Bay, Guangxi, China	[92]
Chartarene A (**117**)	398	C_21_H_34_O_7_	*Niphates recondita* (Sponge, Niphatidae)	Inner coral reef, Beibuwan Bay, Guangxi, China	[92]
Chartarene B (**118**)	282	C_16_H_26_O_4_	*Niphates recondita* (Sponge, Niphatidae)	Inner coral reef, Beibuwan Bay, Guangxi, China	[92]
Chartarene C (**119**)	264	C_16_H_24_O_3_	*Niphates recondita* (Sponge, Niphatidae)	Inner coral reef, Beibuwan Bay, Guangxi, China	[92]
Trichodermol (**120**)	250	C_15_H_22_O_3_	Air and surface samples	Homes enrolled in a case-control study of pulmonary hemosiderosis in infants, Cleveland, Ohio, USA	[93]
Verrucarol (**121**)	266	C_15_H_22_O_4_	Cultured	-	[65,91,94]
	-	-	Straw	Hungarian village of Jaszapati from a field case of mycotoxicosis in horses	[95]
Trichodermin (**122**)	292	C_17_H_24_O_4_	Air and surface samples	Homes enrolled in a case-control study of pulmonary hemosiderosis in infants, Cleveland, Ohio, USA	[93]
Isotrichoverrol B (**123**)	420	C_23_H_32_O_7_	*Niphates recondita* (Sponge, Niphatidae)	Inner coral reef, Beibuwan Bay, Guangxi, China	[92]
Verrol (**124**)	362	C_21_H_30_O_5_	Air and surface samples	Homes enrolled in a case-control study of pulmonary hemosiderosis in infants, Cleveland, Ohio, USA	[93]
	-	-	*Niphates recondita* (Sponge, Niphatidae)	Inner coral reef, Beibuwan Bay, Guangxi, China	[92]
Trichoverrol (**125**)	434	C_24_H_34_O_7_	Cultured	-	[65]
Trichoverrol A (**126**)	434	C_24_H_34_O_7_	Dead animals	Various parts of Hungary and Czechoslovakia	[96]
	-	-	Egyptian paddy grains	Egypt	[97]
Trichoverrol B (**127**)	434	C_24_H_34_O_7_	Dead animals	Various parts of Hungary and Czechoslovakia	[96]
Trichodermadienediol B (**128**)	404	C_23_H_32_O_6_	*Niphates recondita* (Sponge, Niphatidae)	Inner coral reef, Beibuwan Bay, Guangxi, China	[92]
Roridin L-2 (**129**)	544	C_30_H_40_O_9_	Cultured	-	[65,94,98]
	-	-	Air and surface samples	Homes enrolled in a case-control study of pulmonary hemosiderosis in infants, Cleveland, Ohio, USA	[93]
	-	-	*Niphates recondita* (Sponge, Niphatidae)	Inner coral reef, Beibuwan Bay, Guangxi, China	[92]
Chartarene D (**130**)	512	C_29_H_36_O_8_	*Niphates recondita* (Sponge, Niphatidae)	Inner coral reef, Beibuwan Bay, Guangxi, China	[92]
Verrucarin J (**131**)	484	C_27_H_32_O_8_	Cultured	-	[91]
	-	-	Straw	Hungarian village of Jaszapati from a field case of mycotoxicosis in horses	[95]
	-	-	Dead animals	Various parts of Hungary and Czechoslovakia	[96,99]
	-	-	Egyptian paddy grains	Egypt	[97]
Roridin A (**132**)	532	C_29_H_40_O_9_	Cultured	-	[91]
Roridin D (**133**)	530	C_29_H_38_O_9_	Cultured	-	[91]
Roridin E (**134**)	514	C_29_H_38_O_8_	Cultured	-	[65,91,94]
	-	-	Straw	Hungarian village of Jaszapati from a field case of mycotoxicosis in horses	[95]
	-	-	Dead animals	Various parts of Hungary and Czechoslovakia	[96,99]
	-	-	Egyptian paddy grains	Egypt	[97]
	-	-	*Niphates recondita* (Sponge, Niphatidae)	Inner coral reef, Beibuwan Bay, Guangxi, China	[92]
Isororidin E (**135**)	514	C_29_H_38_O_8_	Cultured	-	[65]
Epiroridin E (**136**)	514	C_29_H_38_O_8_	Cultured	-	[65]
Epiisororidin E (**137**)	514	C_29_H_38_O_8_	Cultured	-	[65]
Roridin H (**138**)	512	C_29_H_36_O_8_	Cultured	-	[91]
Muconomycin B (**139**)	484	C_27_H_32_O_8_	*Niphates recondita* (Sponge, Niphatidae)	Inner coral reef, Beibuwan Bay, Guangxi, China	[92]
Satratoxin F (**140**)	542	C_29_H_34_O_10_	Cultured	-	[90]
	-	-	Straw	Hungarian village of Jaszapati from a field case of mycotoxicosis in horses	[95,100]
	-	-	Dead animals	Various parts of Hungary and Czechoslovakia	[96]
	-	-	Egyptian paddy grains	Egypt	[97]
	-	-	Air and surface samples	Homes enrolled in a case-control study of pulmonary hemosiderosis in infants, Cleveland, Ohio, USA	[93]
	-	-	*Sinularia* sp. (Soft coral, Alcyoniidae)	Yongxing Island, South Chian Sea, China	[73]
Isosatratoxin F (**141**)	542	C_29_H_34_O_10_	Air and surface samples	Homes enrolled in a case-control study of pulmonary hemosiderosis in infants, Cleveland, Ohio, USA	[93]
Satratoxin G (**142**)	544	C_29_H_36_O_10_	Cultured	-	[65,90,98]
	-	-	Straw	Hungarian village of Jaszapati from a field case of mycotoxicosis in horses	[95,100]
	-	-	Dead animals	Various parts of Hungary and Czechoslovakia	[96,99]
	-	-	Egyptian paddy grains	Egypt	[97]
	-	-	Bedding Straw of a Sheep Flock with Fatal Stachybotryotoxicosis	Hungaria	[101]
	-	-	-	Finland	[102]
	-	-	Air and surface samples	Homes enrolled in a case-control study of pulmonary hemosiderosis in infants, Cleveland, Ohio, USA	[93]
	-	-	Indoor Air from a Water-Damaged building	Sartorius, Goettingen, Germany	[103]
	-	-	*Niphates recondita* (Sponge, Niphatidae)	Inner coral reef, Beibuwan Bay, Guangxi, China	[92]
Isosatratoxin G (**143**)	544	C_29_H_36_O_10_	Air and surface samples	Homes enrolled in a case-control study of pulmonary hemosiderosis in infants, Cleveland, Ohio, USA	[93]
Satratoxin H (**144**)	528	C_29_H_36_O_9_	Cultured	-	[65,75,91]
	-	-	Straw	Hungarian village of Jaszapati from a field case of mycotoxicosis in horses	[75,95]
	-	-	Dead animals	Various parts of Hungary and Czechoslovakia	[96,99]
	-	-	Egyptian paddy grains	Egypt	[97]
	-	-	-	Finland	[102]
	-	-	Bedding Straw of a Sheep Flock with Fatal Stachybotryotoxicosis	Hungaria	[101]
	-	-	Indoor Air from a Water-Damaged building	Sartorius, Goettingen, Germany	[103]
	-	-	*Sinularia* sp. (Soft coral, Alcyoniidae)	Yongxing Island, South Chian Sea, China	[73]
	-	-	*Niphates recondita* (Sponge, Niphatidae)	Inner coral reef, Beibuwan Bay, Guangxi, China	[92]
Satratoxin H diacetate (**145**)	612	C_33_H_40_O_11_	Cultured	-	[91]
Mytoxin A (**146**)	544	C_29_H_36_O_10_	*Niphates recondita* (Sponge, Niphatidae)	Inner coral reef, Beibuwan Bay, Guangxi, China	[92]
Chartarutine A (**147**)	403	C_23_H_33_NO_5_	*Niphates* sp. (Sponge, Niphatidae)	Near coral reef, Beibuwan Bay, GuangXi, China	[104]
Chartarutine B (**148**)	369	C_23_H_31_NO_3_	*Niphates* sp. (Sponge, Niphatidae)	Near coral reef, Beibuwan Bay, GuangXi, China	[104]
	-	-	Cultured	-	[76]
Chartarutine C (**149**)	449	C_23_H_31_NO_6_S	*Niphates* sp. (Sponge, Niphatidae)	Near coral reef, Beibuwan Bay, GuangXi, China	[104]
Chartarutine D (**150**)	448	C_23_H_32_N_2_O_5_S	*Niphates* sp. (Sponge, Niphatidae)	Near coral reef, Beibuwan Bay, GuangXi, China	[104]
Chartarutine E (**151**)	401	C_23_H_31_NO_5_	*Niphates* sp. (Sponge, Niphatidae)	Near coral reef, Beibuwan Bay, GuangXi, China	[104]
Chartarutine F (**152**)	401	C_23_H_31_NO_5_	*Niphates* sp. (Sponge, Niphatidae)	Near coral reef, Beibuwan Bay, GuangXi, China	[104]
Chartarutine G (**153**)	367	C_23_H_29_NO_3_	*Niphates* sp. (Sponge, Niphatidae)	Near coral reef, Beibuwan Bay, GuangXi, China	[104]
Chartarutine H (**154**)	367	C_23_H_29_NO_3_	*Niphates* sp. (Sponge, Niphatidae)	Near coral reef, Beibuwan Bay, GuangXi, China	[104]
Stachybotrin G (**155**)	503	C_27_H_39_N_2_O_5_S	*Xestospongia testudinaris* (Sponge, Petrosiidae)	Xisha Island, China	[105]
Stachybotrysam A (**156**)	413	C_25_H_35_NO_4_	Cultured	-	[76]
Stachybotrysam B (**157**)	493	C_25_H_35_NO_7_S	Cultured	-	[76]
Stachybotrysam C (**158**)	535	C_27_H_37_NO_8_S	Cultured	-	[76]
Stachybotrysam D (**159**)	611	C_29_H_41_NO_11_S	Cultured	-	[76]
Atranone A (**160**)	416	C_24_H_32_O_6_	Cultured	-	[75,106]
Methylatranone A (**161**)	430	C_25_H_34_O_6_	Cultured	-	[107]
	-	-	*Himerometra magnipinna* (Crinoids, Himerometridae)	Zhanjiang Mangrove National Nature Reserve inGuangdong, China	[108]
Atranone B (**162**)	446	C_25_H_34_O_7_	Cultured	-	[62,75,106]
	-	-	*Himerometra magnipinna* (Crinoids, Himerometridae)	Zhanjiang Mangrove National Nature Reserve, Guangdong, China	[108]
Methylatranone B (**163**)	460	C_26_H_36_O_7_	*Himerometra magnipinna* (Crinoids, Himerometridae)	Zhanjiang Mangrove National Nature Reserve, Guangdong, China	[108]
22-Epimer-methylatranone B (**164**)	460	C_26_H_36_O_7_	*Himerometra magnipinna* (Crinoids, Himerometridae)	Zhanjiang Mangrove National Nature Reserve, Guangdong, China	[108]
Atranone C (**165**)	416	C_24_H_32_O_6_	Cultured	-	[75,106]
Methylatranone C (**166**)	430	C_25_H_34_O_6_	Cultured	-	[107]
Atranone D (**167**)	386	C_24_H_34_O_4_	Cultured	-	[106]
Atranone E (**168**)	386	C_24_H_34_O_4_	Cultured	-	[106]
Atranone F (**169**)	432	C_24_H_32_O_7_	Cultured	-	[106]
Atranone G (**170**)	462	C_25_H_34_O_8_	Cultured	-	[106]
Atranone H (**171**)	432	C_24_H_32_O_7_	Cultured	-	[107]
Atranone I (**172**)	432	C_24_H_32_O_7_	Cultured	-	[107]
Atranone J (**173**)	404	C_23_H_32_O_6_	Cultured	-	[107]
Atranone K (**174**)	448	C_24_H_32_O_8_	Cultured	-	[107]
Atranone L (**175**)	478	C_26_H_38_O_8_	*Himerometra magnipinna* (Crinoids, Himerometridae)	Zhanjiang Mangrove National Nature Reserve, Guangdong, China	[108]
Atranone M (**176**)	476	C_26_H_36_O_8_	*Himerometra magnipinna* (Crinoids, Himerometridae)	Zhanjiang Mangrove National Nature Reserve, Guangdong, China	[108]
Atranone N (**177**)	462	C_26_H_38_O_7_	*Himerometra magnipinna* (Crinoids, Himerometridae)	Zhanjiang Mangrove National Nature Reserve, Guangdong, China	[108]
Atranone O (**178**)	480	C_25_H_36_O_9_	*Himerometra magnipinna* (Crinoids, Himerometridae)	Zhanjiang Mangrove National Nature Reserve, Guangdong, China	[108]
Atranone P (**179**)	404	C_23_H_32_O_6_	*Himerometra magnipinna* (Crinoids, Himerometridae)	Zhanjiang Mangrove National Nature Reserve, Guangdong, China	[108]
Atranone Q (**180**)	390	C_23_H_34_O_5_	*Sarcophyton subviride* (Coral, Alcyoniidae)	Xisha Island, South China Sea, China	[61]
	-	-	Cultured	-	[62]
Atranone R (**181**)	450	C_25_H_38_O_7_	*Sarcophyton subviride* (Coral, Alcyoniidae)	Xisha Island, South China Sea, China	[61]
Atranone S (**182**)	402	C_24_H_34_O_5_	*Sarcophyton subviride* (Coral, Alcyoniidae)	Xisha Island, South China Sea, China	[61]
Atranone T (**183**)	432	C_24_H_32_O_7_	Cultured	-	[62]
Atranone U (**184**)	446	C_25_H_34_O_7_	Cultured	-	[62]
Stachatranone A (**185**)	318	C_20_H_30_O_3_	*Sarcophyton subviride* (Coral, Alcyoniidae)	Xisha Island, South China Sea, China	[61]
Stachatranone B (**186**)	334	C_20_H_30_O_4_	*Sarcophyton subviride* (Coral, Alcyoniidae)	Xisha Island, South China Sea, China	[61]
Stachatranone C (**187**)	334	C_20_H_30_O_4_	*Sarcophyton subviride* (Coral, Alcyoniidae)	Xisha Island, South China Sea, China	[61]
	-	-	Cultured	-	[62]
6α-Hydroxydolabella-3*E*,7*E*,12-trien-14-one (**189**)	302	C_20_H_30_O_2_	Cultured	-	[106]
(1S*,3R*,4R*,6S*,11S*)-3,4-Epoxy-6-hydroxydolabella-7*E*,12-dien-14-one (**188**)	318	C_20_H_30_O_3_	Cultured	-	[106]
(1*R*,6*R*,11*R*)-6-Hydroxydolabella-3*E*,7*E*, 12-trien-14-one (**190**)	302	C_20_H_30_O_2_	*Himerometra magnipinna* (Crinoids, Himerometridae)	Zhanjiang Mangrove National Nature Reserve, Guangdong, China	[108]
Arthproliferin B (**191**)	360	C_22_H_32_O_4_	*Sinularia* sp. (Soft coral, Alcyoniidae)	Yongxing Island, South Chian Sea, China	[73]
Arthproliferin C (**192**)	388	C_23_H_32_O_5_	*Sinularia* sp. (Soft coral, Alcyoniidae)	Yongxing Island, South Chian Sea, China	[73]
Stachybotrychromene A (**193**)	354	C_23_H_30_O_3_	Cultured	-	[64]
Stachybotrychromene B (**194**)	412	C_25_H_32_O_5_	Cultured	-	[64]
Stachybotrychromene C (**195**)	368	C_23_H_28_O_4_	Cultured	-	[64]
Stachybonoid A (**196**)	460	C_26_H_36_O_7_	*Himerometra magnipinna* (Crinoids, Himerometridae)	Zhanjiang Mangrove National Nature Reserve, Guangdong, China	[68]
Stachybonoid B (**197**)	446	C_25_H_34_O_7_	*Himerometra magnipinna* (Crinoids, Himerometridae)	Zhanjiang Mangrove National Nature Reserve, Guangdong, China	[68]
Stachybonoid C (**198**)	388	C_23_H_32_O_5_	*Himerometra magnipinna* (Crinoids, Himerometridae)	Zhanjiang Mangrove National Nature Reserve, Guangdong, China	[68]
*Epi*-cochlioquinone A (**199**)	532	C_30_H_44_O_8_	Cultured	-	[109]
11-*O*-Methyl-epicochlioquinone A (**200**)	546	C_31_H_46_O_8_	Cultured	-	[109]
Arthproliferin D (**201**)	358	C_20_H_22_O_6_	*Sinularia* sp. (Soft coral, Alcyoniidae)	Yongxing Island, South Chian Sea, China	[73]
Pestaxanthone (**202**)	358	C_20_H_22_O_6_	*Sinularia* sp. (Soft coral, Alcyoniidae)	Yongxing Island, South Chian Sea, China	[73]
Prenxanthone (**203**)	340	C_20_H_20_O_5_	*Sinularia* sp. (Soft coral, Alcyoniidae)	Yongxing Island, South Chian Sea, China	[73]
Staprexanthone A (**204**)	394	C_24_H_26_O_5_	Root of an unidentified mangrove plant	Fujian, China	[110]
Staprexanthone B (**205**)	392	C_25_H_28_O_4_	Root of an unidentified mangrove plant	Fujian, China	[110]
Staprexanthone C (**206**)	392	C_25_H_28_O_4_	Root of an unidentified mangrove plant	Fujian, China	[110]
Staprexanthone D (**207**)	392	C_25_H_28_O_4_	Root of an unidentified mangrove plant	Fujian, China	[110]
Staprexanthone E (**208**)	392	C_25_H_28_O_4_	Root of an unidentified mangrove plant	Fujian, China	[110]
Cyclosporin A (**209**)	1202	C_62_H_111_N_11_O_12_	Soil	Hahajima Island Tokyo, Japan	[111]
FR901459 A (**210**)	1218	C_62_H_111_N_11_O_13_	Soil	Hahajima Island Tokyo, Japan	[111]
Arthproliferin A (**211**)	407	C_25_H_29_NO_4_	*Sinularia* sp. (Soft coral, Alcyoniidae)	Yongxing Island, South Chian Sea, China	[73]
5-[(2-Methoxyphenoxy)methyl]-1,3-oxazolidin-2-one (**212**)	223	C_11_H_13_NO_4_	Black mold	Uzbekistan	[112]
Cyclopentanone oxime (**213**)	99	C_5_H_9_NO	Black mold	Uzbekistan	[113]
BR-011 (**214**)	372	C_23_H_32_O_4_	*Niphates* sp. (Sponge, Niphatidae)	Near coral reef, Beibuwan Bay, GuangXi, China	[104]
β-Sitosterol (**215**)	414	C_29_H_50_O	Black mold	Uzbekistan	[112]

**Table 2:** List of biological activities of compounds isolated from *Stachybotrys chartarum*.

Compound Name	Biological Activity	Assay, Organism, or Cell Line	Biological Results	Positive Control	Ref.
Stachybotrysin B (**4**)	Anti-HIV-1 virus	Luciferase/VSV-G	19.2 μM (IC_50_)	Efavirenz 4.0 nM (IC_50_)	[67]
Stachybotrysin C (**6**)	Antiinflammtory	Griess assay/RAW264.7 cells/ LPS-induced NO production inhibition	27.2 μM (IC_50_)	Indomethacin 37.5 μM (IC_50_)	[68]
Stachybotrysin E (**11**)	Anti-HIV-1 virus	Luciferase/VSV-G	20.5 μM (IC_50_)	Efavirenz 4.0 nM (IC_50_)	[67]
	Anti-influenza A virus	Luciferase/VSV-G	45.6 μM (IC_50_)	Ribavirion 2.0 nM (IC_50_)	[67]
	Cytotoxicity	MTT/HepG2	36.2 μM (IC_50_)	Paclitaxel 6.3 nM (IC_50_)	[67]
F1839-I (**12**)	Anti-HIV-1 virus	Luciferase/VSV-G	15.6 μM (IC_50_)	Efavirenz 4.0 nM (IC_50_)	[67]
	Cytotoxicity	MTT/NCI-H460	15.8 μM (IC_50_)	Paclitaxel 1.0 nM (IC_50_)	[67]
		MTT/BGC823	21.9 μM (IC_50_)	Paclitaxel 0.8 nM (IC_50_)	[67]
		MTT/Baoy	41.5 μM (IC_50_)	Paclitaxel 0.4 nM (IC_50_)	[67]
		MTT/HepG2	18.4 μM (IC_50_)	Paclitaxel 6.3 nM (IC_50_)	[67]
Stachybotrysin A (**13**)	Anti-HIV-1 virus	Luciferase/VSV-G	19.6 μM (IC_50_)	Efavirenz 4.0 nM (IC_50_)	[67]
	Anti-influenza A virus	Luciferase/VSV-G	12.4 μM (IC_50_)	Ribavirion 2.0 nM (IC_50_)	[67]
		MTT/Baoy	29.8 μM (IC_50_)	Paclitaxel 0.4 nM (IC_50_)	[67]
		MTT/HepG2	24.7 μM (IC_50_)	Paclitaxel 6.3 nM (IC_50_)	[67]
Stachartin A = Stachybotrysin (**14**)	Anti-influenza A virus	Luciferase/VSV-G	18.7 μM (IC_50_)	Ribavirion 2.0 nM (IC_50_)	[67]
	Cytotoxicity	MTT/HepG2	34.0 μM (IC_50_)	Paclitaxel 6.3 nM (IC_50_)	[67]
Stachybochartin G (**15**)	Cytotoxicity	MTT/MDA-MB231	5.6 μM (IC_50_)	-Cisplatin 11.3 μM (IC_50_) -Doxorubicin 1.0 μM (IC_50_)	[70]
		MTT/U2-OS	4.5 μM (IC_50_)	-Cisplatin 5.9 μM (IC_50_) -Doxorubicin 1.2 μM (IC_50_)	[70]
Stachybotrysin F (**16**)	Anti-HIV-1 virus	Luciferase/VSV-G	35.7 μM (IC_50_)	Efavirenz 4.0 nM (IC_50_)	[67]
	Anti-influenza A virus	Luciferase/VSV-G	14.6 μM (IC_50_)	Ribavirion 2.0 nM (IC_50_)	[67]
	Cytotoxicity	MTT/HCT116	48.5 μM (IC_50_)	Paclitaxel 1.0 nM (IC_50_)	[67]
		MTT/NCI-H460	41.2 μM (IC_50_)	Paclitaxel 1.0 nM (IC_50_)	[67]
		MTT/BGC823	41.7 μM (IC_50_)	Paclitaxel 0.8 nM (IC_50_)	[67]
		MTT/Baoy	30.6 μM (IC_50_)	Paclitaxel 0.4 nM (IC_50_)	[67]
		MTT/HepG2	28.4 μM (IC_50_)	Paclitaxel 6.3 nM (IC_50_)	[67]
Stachybotrysin G (**17**)	Anti-HIV-1 virus	Luciferase/VSV-G	18.1 μM (IC_50_)	Efavirenz 4.0 nM (IC_50_)	[67]
	Anti-influenza A virus	Luciferase/VSV-G	23.4 μM (IC_50_)	Ribavirion 2.0 nM (IC_50_)	[67]
	Cytotoxicity	MTT/HCT116	44.6 μM (IC_50_)	Paclitaxel 1.0 nM (IC_50_)	[67]
		MTT/NCI-H460	26.9 μM (IC_50_)	Paclitaxel 1.0 nM (IC_50_)	[67]
		MTT/BGC823	31.6 μM (IC_50_)	Paclitaxel 0.8 nM (IC_50_)	[67]
		MTT/Baoy	56.9 μM (IC_50_)	Paclitaxel 0.4 nM (IC_50_)	[67]
		MTT/HepG2	24.6 μM (IC_50_)	Paclitaxel 6.3 nM (IC_50_)	[67]
Stachybotrysin H (**18**)	Potassium channel inhibition	Kv1.3 FLIPR thallium flux /CHO-Kv1.3	13.4 μM (IC_50_)	-Clofazamine 2.01 μM (IC_50_) -PAP-1 0.17 μM (IC_50_)	[71]
Stachybotrysin I (**19**)	Potassium channel inhibition	Kv1.3 FLIPR thallium flux /CHO-Kv1.3	10.9 μM (IC_50_)	-Clofazamine 2.01 μM (IC_50_) -PAP-1 0.17 μM (IC_50_)	[71]
Stachybotrylactone = Stachybotrolide (**20**)	Antiinflammtory	Griess assay/RAW264.7 cells/ LPS-induced NO production inhibition	17.9 μM (IC_50_)	Indomethacin 37.5 μM (IC_50_)	[68]
Stachybotrylactone acetate (**21**)	Cytotoxicity	MTT/HL-60	11.44 μM (IC_50_)	Cisplatin 1.25 μM (IC_50_)	[72]
		MTT/SMMC-7721	23.31 μM (IC_50_)	Cisplatin 7.77 μM (IC_50_)	[72]
		MTT/A-549	22.84 μM (IC_50_)	Cisplatin 6.12 μM (IC_50_)	[72]
		MTT/MCF-7	18.20 μM (IC_50_)	Cisplatin 17.63 μM (IC_50_)	[72]
		MTT/SW480	17.38 μM (IC_50_)	Cisplatin 14.58 μM (IC_50_)	[72]
	Anti-influenza A virus	Luciferase/VSV-G	18.9 μM (IC_50_)	Ribavirion 2.0 nM (IC_50_)	[67]
Stachybotrane A (**22**)	Cytotoxicity	MTT/HL-60	9.23 μM (IC_50_)	Cisplatin 1.25 μM (IC_50_)	[72]
		MTT/SMMC-7721	19.67 μM (IC_50_)	Cisplatin 7.77 μM (IC_50_)	[72]
		MTT/A-549	31.22 μM (IC_50_)	Cisplatin 6.12 μM (IC_50_)	[72]
		MTT/MCF-7	18.74 μM (IC_50_)	Cisplatin 17.63 μM (IC_50_)	[72]
Stachybotrane B (**23**)	Cytotoxicity	MTT/HL-60	15.08 μM (IC_50_)	Cisplatin 1.25 μM (IC_50_)	[72]
		MTT/SMMC-7721	24.90 μM (IC_50_)	Cisplatin 7.77 μM (IC_50_)	[72]
		MTT/MCF-7	29.34 μM (IC_50_)	Cisplatin 17.63 μM (IC_50_)	[72]
		MTT/SW480	31.03 μM (IC_50_)	Cisplatin 14.58 μM (IC_50_)	[72]
K-76-1 (**36**)	Tyrosine kinase inhibition	Radiometry/^33^P	>0.2 mM (IC_50_)	-	[77]
K-76-2 (**37**)	Tyrosine kinase inhibition	Radiometry/^33^P	>0.031 mM (IC_50_)	-	[77]
Stachybonoid F (**48**)	Antiinflammtory	Griess assay/RAW264.7 cells/ LPS-induced NO production inhibition	52.5 μM (IC_50_)	Indomethacin 37.5 μM (IC_50_)	[68]
K-76-3 (**59**)	Tyrosine kinase inhibition	Radiometry/^33^P	>0.4 mM (IC_50_)	-	[77]
K-76-4 (**60**)	Tyrosine kinase inhibition	Radiometry/^33^P	>0.025 mM (IC_50_)	-	[77]
K-76-5 (**61**)	Tyrosine kinase inhibition	Radiometry/^33^P	>0.097 mM (IC_50_)	-	[77]
K-76-6 (**62**)	Tyrosine kinase inhibition	Radiometry/^33^P	>0.146 mM (IC_50_)	-	[77]
K-76-7 (**63**)	Tyrosine kinase inhibition	Radiometry/^33^P	>0.046 mM (IC_50_)	-	[77]
Bistachybotrysin A (**73**)	Cytotoxicity	MTT/HCT116	6.7 μM (IC_50_)	Paclitaxel 0.0038 μM (IC_50_)	[79]
		MTT/NCI-H460	7.5 μM (IC_50_)	Paclitaxel 0.0004 μM (IC_50_)	[79]
		MTT/BGC823	14.2 μM (IC_50_)	Paclitaxel 0.002 μM (IC_50_)	[79]
		MTT/Daoy	2.8 μM (IC_50_)	Paclitaxel 0.0002 μM (IC_50_)	[79]
		MTT/HepG2	11.9 μM (IC_50_)	Paclitaxel 0.0102 μM (IC_50_)	[79]
Bistachybotrysin B (**74**)	Cytotoxicity	MTT/HCT116	18.0 μM (IC_50_)	Paclitaxel 0.0038 μM (IC_50_)	[79]
		MTT/NCI-H460	5.5 μM (IC_50_)	Paclitaxel 0.0004 μM (IC_50_)	[79]
		MTT/BGC823	6.6 μM (IC_50_)	Paclitaxel 0.002 μM (IC_50_)	[79]
		MTT/Daoy	4.2 μM (IC_50_)	Paclitaxel 0.0002 μM (IC_50_)	[79]
		MTT/HepG2	19.3 μM (IC_50_)	Paclitaxel 0.0102 μM (IC_50_)	[79]
Bistachybotrysin C (**75**)	Cytotoxicity	MTT/HCT116	19.1 μM (IC_50_)	Paclitaxel 0.0038 μM (IC_50_)	[79]
		MTT/NCI-H460	12.3 μM (IC_50_)	Paclitaxel 0.0004 μM (IC_50_)	[79]
		MTT/BGC823	19.2 μM (IC_50_)	Paclitaxel 0.002 μM (IC_50_)	[79]
		MTT/Daoy	14.6 μM (IC_50_)	Paclitaxel 0.0002 μM (IC_50_)	[79]
		MTT/HepG2	19.3 μM (IC_50_)	Paclitaxel 0.0102 μM (IC_50_)	[79]
Bistachybotrysin D (**76**)	Cytotoxicity	MTT/HCT116	6.8 μM (IC_50_)	Paclitaxel 0.00381 μM (IC_50_)	[80]
		MTT/NCI-H460	14.7 μM (IC_50_)	Paclitaxel 0.000384 μM (IC_50_)	[80]
		MTT/BGC823	11.4 μM (IC_50_)	Paclitaxel 0.00197 μM (IC_50_)	[80]
		MTT/Daoy	11.6 μM (IC_50_)	Paclitaxel 0.000187 μM (IC_50_)	[80]
		MTT/HepG2	7.5 μM (IC_50_)	Paclitaxel 0.0102 μM (IC_50_)	[80]
	Antiinflammtory	LPS/BV2/NO production inhibition	61.1% at 10.0 μM	Curcumin 67.6% at 10.0 μM	[80]
Bistachybotrysin E (**77**)	Cytotoxicity	MTT/HCT116	8.9 μM (IC_50_)	Paclitaxel 0.00381 μM (IC_50_)	[80]
		MTT/NCI-H460	19.0 μM (IC_50_)	Paclitaxel 0.000384 μM (IC_50_)	[80]
		MTT/BGC823	6.7 μM (IC_50_)	Paclitaxel 0.00197 μM (IC_50_)	[80]
		MTT/Daoy	59.0 μM (IC_50_)	Paclitaxel 0.000187 μM (IC_50_)	[80]
		MTT/HepG2	12.4 μM (IC_50_)	Paclitaxel 0.0102 μM (IC_50_)	[80]
Bistachybotrysin F (**78**)	Cytotoxicity	MTT/HCT116	22.8 μM (IC_50_)	Taxol 0.00381 μM (IC_50_)	[81]
		MTT/NCI-H460	22.5 μM (IC_50_)	Taxol 0.000384 μM (IC_50_)	[81]
		MTT/BGC823	18.3 μM (IC_50_)	Taxol 0.00197 μM (IC_50_)	[81]
		MTT/Daoy	61.8 μM (IC_50_)	Taxol 0.000187 μM (IC_50_)	[81]
		MTT/HepG2	18.3 μM (IC_50_)	Taxol 0.0102 μM (IC_50_)	[81]
Bistachybotrysin G (**79**)	Cytotoxicity	MTT/HepG2	22.7 μM (IC_50_)	Taxol 0.0102 μM (IC_50_)	[81]
Bistachybotrysin H (**80**)	Cytotoxicity	MTT/HCT116	20.7 μM (IC_50_)	Taxol 0.00381 μM (IC_50_)	[81]
		MTT/NCI-H460	10.6 μM (IC_50_)	Taxol 0.000384 μM (IC_50_)	[81]
		MTT/BGC823	21.1 μM (IC_50_)	Taxol 0.00197 μM (IC_50_)	[81]
		MTT/Daoy	20.7 μM (IC_50_)	Taxol 0.000187 μM (IC_50_)	[81]
		MTT/HepG2	18.4 μM (IC_50_)	Taxol 0.0102 μM (IC_50_)	[81]
Bistachybotrysin I (**81**)	Cytotoxicity	MTT/HCT116	9.1 μM (IC_50_)	Taxol 0.00381 μM (IC_50_)	[81]
		MTT/NCI-H460	19.9 μM (IC_50_)	Taxol 0.000384 μM (IC_50_)	[81]
		MTT/BGC823	17.2 μM (IC_50_)	Taxol 0.00197 μM (IC_50_)	[81]
		MTT/Daoy	18.4 μM (IC_50_)	Taxol 0.000187 μM (IC_50_)	[81]
		MTT/HepG2	21.4 μM (IC_50_)	Taxol 0.0102 μM (IC_50_)	[81]
Bistachybotrysin J (**82**)	Cytotoxicity	MTT/HCT116	15.8 μM (IC_50_)	Taxol 0.00381 μM (IC_50_)	[81]
		MTT/NCI-H460	20.4 μM (IC_50_)	Taxol 0.000384 μM (IC_50_)	[81]
		MTT/BGC823	16.9 μM (IC_50_)	Taxol 0.00197 μM (IC_50_)	[81]
		MTT/Daoy	25.4 μM (IC_50_)	Taxol 0.000187 μM (IC_50_)	[81]
		MTT/HepG2	12.2 μM (IC_50_)	Taxol 0.0102 μM (IC_50_)	[81]
Bistachybotrysin K (**83**)	Cytotoxicity	MTT/HCT116	3.4 μM (IC_50_)	Paclitaxel 0.0038 μM (IC_50_)	[82]
		MTT/NCI-H460	4.7 μM (IC_50_)	Paclitaxel 0.0004 μM (IC_50_)	[82]
		MTT/BGC823	3.3 μM (IC_50_)	Paclitaxel 0.0002 μM (IC_50_)	[82]
		MTT/Daoy	1.1 μM (IC_50_)	Paclitaxel 0.0002 μM (IC_50_)	[82]
		MTT/HepG2	4.3 μM (IC_50_)	Paclitaxel 0.0102 μM (IC_50_)	[82]
Bistachybotrysin L (**84**)	Neuroprotective	MTT/SK-N-SH	0.15 % at 10.0 μM, ↑ cell viability	Resveratrol 16.1% at 10.0 μM, ↑ cell viability	[83]
	Cytotoxicity	MTT/HCT116	10.6 μM (IC_50_)	Paclitaxel 0.038 μM (IC_50_)	[83]
		MTT/NCI-H460	13.5 μM (IC_50_)	Paclitaxel 0.004 μM (IC_50_)	[83]
		MTT/BGC823	22.3 μM (IC_50_)	Paclitaxel 0.002 μM (IC_50_)	[83]
		MTT/HepG2	18.9 μM (IC_50_)	Paclitaxel 0.0102 μM (IC_50_)	[83]
	Antiinflammtory	LPS/BV2/NO production inhibition	5.31% at 10.0 μM	Curcumin 67.6% at 10.0 μM	[83]
Bistachybotrysin M (**85**)	Neuroprotective	MTT/SK-N-SH	17.4 % at 10.0 μM, ↑ cell viability	Resveratrol 16.1% at 10.0 μM, ↑ cell viability	[83]
	Cytotoxicity	MTT/HCT116	2.5 μM (IC_50_)	Paclitaxel 0.0038 μM (IC_50_)	[83]
		MTT/NCI-H460	3.5 μM (IC_50_)	Paclitaxel 0.0004 μM (IC_50_)	[83]
		MTT/BGC823	1.8 μM (IC_50_)	Paclitaxel 0.002 μM (IC_50_)	[83]
		MTT/Daoy	2.4 μM (IC_50_)	Paclitaxel 0.0002 μM (IC_50_)	[83]
		MTT/HepG2	2.2 μM (IC_50_)	Paclitaxel 0.0102 μM (IC_50_)	[83]
	Antiinflammtory	LPS/BV2/NO production inhibition	26.3% at 10.0 μM	Curcumin 67.6% at 10.0 μM	[83]
Bistachybotrysin N (**86**)	Neuroprotective	MTT/SK-N-SH	17.6 % at 10.0 μM, ↑ cell viability	Resveratrol 16.1% at 10.0 μM, ↑ cell viability	[83]
	Cytotoxicity	MTT/HCT116	64.5 μM (IC_50_)	Paclitaxel 0.0038 μM (IC_50_)	[83]
		MTT/NCI-H460	8.3 μM (IC_50_)	Paclitaxel 0.0004 μM (IC_50_)	[83]
		MTT/BGC823	12.5 μM (IC_50_)	Paclitaxel 0.002 μM (IC_50_)	[83]
		MTT/Daoy	61.4 μM (IC_50_)	Paclitaxel 0.0002 μM (IC_50_)	[83]
		MTT/HepG2	56.1 μM (IC_50_)	Paclitaxel 0.0102 μM (IC_50_)	[83]
Bistachybotrysin O (**87**)	Neuroprotective	MTT/SK-N-SH	8.4 % at 10.0 μM, ↑ cell viability	Resveratrol 16.1% at 10.0 μM, ↑ cell viability	[83]
	Cytotoxicity	MTT/HCT116	18.8 μM (IC_50_)	Paclitaxel 0.0038 μM (IC_50_)	[83]
		MTT/NCI-H460	11.5 μM (IC_50_)	Paclitaxel 0.0004 μM (IC_50_)	[83]
		MTT/BGC823	20.5 μM (IC_50_)	Paclitaxel 0.002 μM (IC_50_)	[83]
		MTT/Daoy	10.7 μM (IC_50_)	Paclitaxel 0.0002 μM (IC_50_)	[83]
		MTT/HepG2	20.1 μM (IC_50_)	Paclitaxel 0.0102 μM (IC_50_)	[83]
Bistachybotrysin P (**88**)	Antiinflammtory	LPS/BV2/NO production inhibition	12.9% at 10.0 μM	Curcumin 67.6% at 10.0 μM	[83]
Bistachybotrysin Q (**89**)	Antiinflammtory	LPS/BV2/NO production inhibition	10.1% at 10.0 μM	Curcumin 67.6% at 10.0 μM	[83]
	Cytotoxicity	MTT/HCT116	18.5 μM (IC_50_)	Paclitaxel 0.038 μM (IC_50_)	[83]
		MTT/NCI-H460	8.8 μM (IC_50_)	Paclitaxel 0.004 μM (IC_50_)	[83]
		MTT/BGC823	55.4 μM (IC_50_)	Paclitaxel 0.002 μM (IC_50_)	[83]
		MTT/Daoy	17.3 μM (IC_50_)	Paclitaxel 0.0002 μM (IC_50_)	[83]
		MTT/HepG2	14.1 μM (IC_50_)	Paclitaxel 0.0102 μM (IC_50_)	[83]
Bistachybotrysin R (**90**)	Cytotoxicity	MTT/HCT116	8.8 μM (IC_50_)	Paclitaxel 0.0038 μM (IC_50_)	[83]
		MTT/NCI-H460	8.2 μM (IC_50_)	Paclitaxel 0.0004 μM (IC_50_)	[83]
		MTT/BGC823	17.8 μM (IC_50_)	Paclitaxel 0.002 μM (IC_50_)	[83]
		MTT/Daoy	13.1 μM (IC_50_)	Paclitaxel 0.0002 μM (IC_50_)	[83]
		MTT/HepG2	9.4 μM (IC_50_)	Paclitaxel 0.0102 μM (IC_50_)	[83]
Bistachybotrysin S (**91**)	Neuroprotective	MTT/SK-N-SH	6.5 % at 10.0 μM, ↑ cell viability	Resveratrol 16.1% at 10.0 μM, ↑ cell viability	[83]
	Cytotoxicity	MTT/HCT116	8.0 μM (IC_50_)	Paclitaxel 0.0038 μM (IC_50_)	[83]
		MTT/NCI-H460	11.7 μM (IC_50_)	Paclitaxel 0.0004 μM (IC_50_)	[83]
		MTT/BGC823	8.7 μM (IC_50_)	Paclitaxel 0.002 μM (IC_50_)	[83]
		MTT/Daoy	11.8 μM (IC_50_)	Paclitaxel 0.0002 μM (IC_50_)	[83]
		MTT/HepG2	6.0 μM (IC_50_)	Paclitaxel 0.0102 μM (IC_50_)	[83]
	Antiinflammtory	LPS/BV2/NO production inhibition	54.2 % at 10.0 μM	Curcumin 67.6% at 10.0 μM	[83]
Bistachybotrysin T (**92**)	Neuroprotective	MTT/SK-N-SH	17.4 % at 10.0 μM, ↑ cell viability	Resveratrol 16.1% at 10.0 μM, ↑ cell viability	[83]
Bistachybotrysin U (**93**)	Neuroprotective	MTT/SK-N-SH	9.3 % at 10.0 μM, ↑ cell viability	Resveratrol 16.1% at 10.0 μM, ↑ cell viability	[83]
	Cytotoxicity	MTT/HCT116	9.7 μM (IC_50_)	Paclitaxel 0.038 μM (IC_50_)	[83]
		MTT/NCI-H460	10.1 μM (IC_50_)	Paclitaxel 0.004 μM (IC_50_)	[83]
		MTT/BGC823	9.8 μM (IC_50_)	Paclitaxel 0.002 μM (IC_50_)	[83]
		MTT/Daoy	8.1 μM (IC_50_)	Paclitaxel 0.0002 μM (IC_50_)	[83]
		MTT/HepG2	9.4 μM (IC_50_)	Paclitaxel 0.0102 μM (IC_50_)	[83]
Bistachybotrysin V (**94**)	Cytotoxicity	MTT/HCT116	15.0 μM (IC_50_)	Paclitaxel 0.0038 μM (IC_50_)	[83]
		MTT/NCI-H460	10.9 μM (IC_50_)	Paclitaxel 0.0004 μM (IC_50_)	[83]
		MTT/BGC823	23.9 μM (IC_50_)	Paclitaxel 0.002 μM (IC_50_)	[83]
		MTT/Daoy	27.7 μM (IC_50_)	Paclitaxel 0.0002 μM (IC_50_)	[83]
		MTT/HepG2	12.9 μM (IC_50_)	Paclitaxel 0.0102 μM (IC_50_)	[83]
Bistachybotrysin W (**95**)	Cytotoxicity	MTT/HCT116	12.1 μM (IC_50_)	Paclitaxel 0.0038 μM (IC_50_)	[84]
		MTT/NCI-H460	11.5 μM (IC_50_)	Paclitaxel 0.0004 μM (IC_50_)	[84]
		MTT/BGC823	13.2 μM (IC_50_)	Paclitaxel 0.002 μM (IC_50_)	[84]
		MTT/Daoy	8.8 μM (IC_50_)	Paclitaxel 0.0002 μM (IC_50_)	[84]
		MTT/HepG2	7.0 μM (IC_50_)	Paclitaxel 0.0102 μM (IC_50_)	[84]
Bistachybotrysin X (**96**)	Cytotoxicity	MTT/HCT116	22.6 μM (IC_50_)	Paclitaxel 0.0038 μM (IC_50_)	[84]
		MTT/NCI-H460	10.8 μM (IC_50_)	Paclitaxel 0.0004 μM (IC_50_)	[84]
		MTT/BGC823	15.1 μM (IC_50_)	Paclitaxel 0.002 μM (IC_50_)	[84]
		MTT/Daoy	21.5 μM (IC_50_)	Paclitaxel 0.0002 μM (IC_50_)	[84]
		MTT/HepG2	11.5 μM (IC_50_)	Paclitaxel 0.0102 μM (IC_50_)	[84]
Bistachybotrysin Y (**97**)	Cytotoxicity	MTT/HCT116	5.9 μM (IC_50_)	Paclitaxel 0.0038 μM (IC_50_)	[84]
		MTT/NCI-H460	13.0 μM (IC_50_)	Paclitaxel 0.0004 μM (IC_50_)	[84]
		MTT/BGC823	14.1 μM (IC_50_)	Paclitaxel 0.002 μM (IC_50_)	[84]
		MTT/Daoy	6.4 μM (IC_50_)	Paclitaxel 0.0002 μM (IC_50_)	[84]
		MTT/HepG2	9.8 μM (IC_50_)	Paclitaxel 0.0102 μM (IC_50_)	[84]
Chartarolide A (**98**)	Cytotoxicity	MTT/HCT-116	1.9 μM (IC_50_)	Taxol 0.03 μM (IC_50_)	[85]
		MTT/HepG2	1.8 μM (IC_50_)	Taxol 0.02 μM (IC_50_)	[85]
		MTT/BGC-823	1.3 μM (IC_50_)	Taxol 0.001 μM (IC_50_)	[85]
		MTT/NCI-H1650	5.5 μM (IC_50_)	Taxol 0.07 μM (IC_50_)	[85]
		MTT/A2780	1.5 μM (IC_50_)	Taxol 0.03 μM (IC_50_)	[85]
		MTT/MCF-7	1.4 μM (IC_50_)	Taxol 0.09 μM (IC_50_)	[85]
	Tumor-related kinases inhibition	Spectrophotometric/FGFR3	2.6 μM (IC_50_)	Satratoxin H ˂0.5 μM (IC_50_)	[85]
		Spectrophotometric/IGF1R	6.8 μM (IC_50_)	Satratoxin H ˂0.5 μM (IC_50_)	[85]
		Spectrophotometric/PDGFRb	9.1 μM (IC_50_)	Satratoxin H ˂0.5 μM (IC_50_)	[85]
		Spectrophotometric/TRKB	8.0 μM (IC_50_)	Satratoxin H ˂0.5 μM (IC_50_)	[85]
Chartarolide B (**99**)	Cytotoxicity	MTT/HCT-116	2.3 μM (IC_50_)	Taxol 0.03 μM (IC_50_)	[85]
		MTT/HepG2	2.8 μM (IC_50_)	Taxol 0.02 μM (IC_50_)	[85]
		MTT/BGC-823	1.6 μM (IC_50_)	Taxol 0.001 μM (IC_50_)	[85]
		MTT/NCI-H1650	4.8 μM (IC_50_)	Taxol 0.07 μM (IC_50_)	[85]
		MTT/A2780	3.2 μM (IC_50_)	Taxol 0.03 μM (IC_50_)	[85]
		MTT/MCF-7	3.8 μM (IC_50_)	Taxol 0.09 μM (IC_50_)	[85]
	Tumor-related kinases inhibition	Spectrophotometric/FGFR3	4.9 μM (IC_50_)	Satratoxin H ˂0.5 μM (IC_50_)	[85]
		Spectrophotometric/IGF1R	8.4 μM (IC_50_)	Satratoxin H ˂0.5 μM (IC_50_)	[85]
		Spectrophotometric/PDGFRb	20.3 μM (IC_50_)	Satratoxin H ˂0.5 μM (IC_50_)	[85]
		Spectrophotometric/TRKB	11.3 μM (IC_50_)	Satratoxin H ˂0.5 μM (IC_50_)	[85]
Chartarolide C (**100**)	Cytotoxicity	MTT/HCT-116	7.8 μM (IC_50_)	Taxol 0.03 μM (IC_50_)	[85]
		MTT/HepG2	8.9 μM (IC_50_)	Taxol 0.02 μM (IC_50_)	[85]
		MTT/BGC-823	5.4 μM (IC_50_)	Taxol 0.001 μM (IC_50_)	[85]
		MTT/NCI-H1650	11.3 μM (IC_50_)	Taxol 0.07 μM (IC_50_)	[85]
		MTT/A2780	12.5 μM (IC_50_)	Taxol 0.03 μM (IC_50_)	[85]
		MTT/MCF-7	8.7 μM (IC_50_)	Taxol 0.09 μM (IC_50_)	[85]
	Tumor-related kinases inhibition	Spectrophotometric/FGFR3	21.4 μM (IC_50_)	Satratoxin H ˂0.5 μM (IC_50_)	[85]
		Spectrophotometric/TRKB	18.8 μM (IC_50_)	Satratoxin H ˂0.5 μM (IC_50_)	[85]
Chartarlactam Q (**101**)	Antibacterial	Broth microdilution/*S. aureus* ATCC 29213	8.0 μg/mL (MIC)	Chloramphenicol 1.0 μg/mL (MIC)	[86]
Chartarlactam R (**102**)	Antibacterial	Broth microdilution/*S. aureus* ATCC 29213	16.0 μg/mL (MIC)	Chloramphenicol 1.0 μg/mL (MIC)	[86]
Chartarlactam S (**103**)	Antibacterial	Broth microdilution/*S. aureus* ATCC 29213	4.0 μg/mL (MIC)	Chloramphenicol 1.0 μg/mL (MIC)	[86]
Stachybochartin A (**105**)	Cytotoxicity	MTT/MDA-MB231	21.7 μM (IC_50_)	-Cisplatin 11.3 μM (IC_50_) -Doxorubicin 1.0 μM (IC_50_)	[70]
		MTT/U2-OS	19.8 μM (IC_50_)	-Cisplatin 5.9 μM (IC_50_) -Doxorubicin 1.2 μM (IC_50_)	[70]
Stachybochartin B (**106**)	Cytotoxicity	MTT/MDA-MB231	17.6 μM (IC_50_)	-Cisplatin 11.3 μM (IC_50_) -Doxorubicin 1.0 μM (IC_50_)	[70]
		MTT/U2-OS	11.2 μM (IC_50_)	-Cisplatin 5.9 μM (IC_50_) -Doxorubicin 1.2 μM (IC_50_)	[70]
Stachybochartin C (**107**)	Cytotoxicity	MTT/MDA-MB231	11.6 μM (IC_50_)	-Cisplatin 11.3 μM (IC_50_) -Doxorubicin 1.0 μM (IC_50_)	[70]
		MTT/U2-OS	14.5 μM (IC_50_)	-Cisplatin 5.9 μM (IC_50_) -Doxorubicin 1.2 μM (IC_50_)	[70]
Stachybochartin D (**108**)	Cytotoxicity	MTT/MDA-MB231	10.4 μM (IC_50_)	-Cisplatin 11.3 μM (IC_50_) -Doxorubicin 1.0 μM (IC_50_)	[70]
		MTT/U2-OS	9.2 μM (IC_50_)	-Cisplatin 5.9 μM (IC_50_) -Doxorubicin 1.2 μM (IC_50_)	[70]
Stachyin B (**109**)	Antibacterial	Broth microdilution/*S. aureus* ATCC 29213	4.0 μg/mL (MIC)	Chloramphenicol 1.0 μg/mL (MIC)	[86]
2,4,12-Trihydroxyapotrichothecene (**116**)	Cytotoxicity	MTT/HCT-116	0.87 μM (IC_50_)	Taxol 0.03 μM (IC_50_)	[92]
		MTT/HepG2	0.69 μM (IC_50_)	Taxol 0.01 μM (IC_50_)	[92]
		MTT/BGC-823	0.65 μM (IC_50_)	Taxol 0.01 μM (IC_50_)	[92]
		MTT/NCI-H1650	0.84 μM (IC_50_)	Taxol 0.04 μM (IC_50_)	[92]
		MTT/A2780	0.69 μM (IC_50_)	Taxol 0.01 μM (IC_50_)	[92]
	Tumor-related kinases inhibition	Spectrophotometric/FGFR3	0.5 μM (IC_50_)	-	[92]
		Spectrophotometric/IGF1R	1.0 μM (IC_50_)	-	[92]
		Spectrophotometric/PDGFRb	2.1 μM (IC_50_)	-	[92]
		Spectrophotometric/TRKB	1.0 μM (IC_50_)	-	[92]
		Spectrophotometric/WT	2.2 μM (IC_50_)	-	[92]
Chartarene A (**117**)	Cytotoxicity	MTT/HCT-116	3.39 μM (IC_50_)	Taxol 0.03 μM (IC_50_)	[92]
		MTT/HepG2	3.95 μM (IC_50_)	Taxol 0.01 μM (IC_50_)	[92]
		MTT/BGC-823	2.87 μM (IC_50_)	Taxol 0.01 μM (IC_50_)	[92]
		MTT/A2780	2.38 μM (IC_50_)	Taxol 0.01 μM (IC_50_)	[92]
Chartarene B (**118**)	Cytotoxicity	MTT/HCT-116	5.58 μM (IC_50_)	Taxol 0.03 μM (IC_50_)	[92]
	Tumor-related kinases inhibition	Spectrophotometric/FGFR3	2.4 μM (IC_50_)	-	[92]
		Spectrophotometric/IGF1R	6.9 μM (IC_50_)	-	[92]
		Spectrophotometric/PDGFRb	10.4 μM (IC_50_)	-	[92]
		Spectrophotometric/TRKB	7.0 μM (IC_50_)	-	[92]
		Spectrophotometric/WT	12.9 μM (IC_50_)	-	[92]
Chartarene C (**119**)	Cytotoxicity	MTT/HCT-116	0.74 μM (IC_50_)	Taxol 0.03 μM (IC_50_)	[92]
		MTT/HepG2	2.09 μM (IC_50_)	Taxol 0.01 μM (IC_50_)	[92]
		MTT/NCI-H1650	2.58 μM (IC_50_)	Taxol 0.04 μM (IC_50_)	[92]
		MTT/A2780	2.07 μM (IC_50_)	Taxol 0.01 μM (IC_50_)	[92]
	Tumor-related kinases inhibition	Spectrophotometric/FGFR3	1.1 μM (IC_50_)	-	[92]
		Spectrophotometric/IGF1R	3.0 μM (IC_50_)	-	[92]
		Spectrophotometric/PDGFRb	5.3 μM (IC_50_)	-	[92]
		Spectrophotometric/TRKB	2.7 μM (IC_50_)	-	[92]
		Spectrophotometric/WT	6.3 μM (IC_50_)	-	[92]
Trichodermol (**120**)	Cytotoxicity	MTT/HCT-116	7.22 μM (IC_50_)	Taxol 0.03 μM (IC_50_)	[92]
		MTT/HepG2	3.69 μM (IC_50_)	Taxol 0.01 μM (IC_50_)	[92]
		MTT/BGC-823	2.55 μM (IC_50_)	Taxol 0.01 μM (IC_50_)	[92]
		MTT/NCI-H1650	2.68 μM (IC_50_)	Taxol 0.04 μM (IC_50_)	[92]
		MTT/A2780	2.68 μM (IC_50_)	Taxol 0.01 μM (IC_50_)	[92]
	Tumor-related kinases inhibition	Spectrophotometric/FGFR3	0.9 μM (IC_50_)	-	[92]
		Spectrophotometric/IGF1R	0.8 μM (IC_50_)	-	[92]
		Spectrophotometric/PDGFRb	3.1 μM (IC_50_)	-	[92]
		Spectrophotometric/TRKB	1.8 μM (IC_50_)	-	[92]
		Spectrophotometric/WT	3.0 μM (IC_50_)	-	[92]
Isotrichoverrol B (**123**)	Cytotoxicity	MTT/HCT-116	3.48 μM (IC_50_)	Taxol 0.03 μM (IC_50_)	[92]
		MTT/HepG2	2.62 μM (IC_50_)	Taxol 0.01 μM (IC_50_)	[92]
		MTT/BGC-823	2.64 μM (IC_50_)	Taxol 0.01 μM (IC_50_)	[92]
		MTT/NCI-H1650	2.36 μM (IC_50_)	Taxol 0.04 μM (IC_50_)	[92]
		MTT/A2780	2.12 μM (IC_50_)	Taxol 0.01 μM (IC_50_)	[92]
	Tumor-related kinases inhibition	Spectrophotometric/FGFR3	24.0 μM (IC_50_)	-	[92]
Verrol (**124**)	Cytotoxicity	MTT/HCT-116	2.77 μM (IC_50_)	Taxol 0.03 μM (IC_50_)	[92]
		MTT/HepG2	1.45 μM (IC_50_)	Taxol 0.01 μM (IC_50_)	[92]
		MTT/BGC-823	2.33 μM (IC_50_)	Taxol 0.01 μM (IC_50_)	[92]
		MTT/NCI-H1650	2.68 μM (IC_50_)	Taxol 0.04 μM (IC_50_)	[92]
		MTT/A2780	2.00 μM (IC_50_)	Taxol 0.01 μM (IC_50_)	[92]
	Tumor-related kinases inhibition	Spectrophotometric/FGFR3	0.1 μM (IC_50_)	-	[92]
		Spectrophotometric/IGF1R	0.2 μM (IC_50_)	-	[92]
		Spectrophotometric/PDGFRb	0.7 μM (IC_50_)	-	[92]
		Spectrophotometric/TRKB	0.4 μM (IC_50_)	-	[92]
		Spectrophotometric/WT	0.9 μM (IC_50_)	-	[92]
Trichodermadienediol B (**128**)	Cytotoxicity	MTT/HCT-116	1.65 μM (IC_50_)	Taxol 0.03 μM (IC_50_)	[92]
		MTT/HepG2	0.86 μM (IC_50_)	Taxol 0.01 μM (IC_50_)	[92]
		MTT/BGC-823	0.81 μM (IC_50_)	Taxol 0.01 μM (IC_50_)	[92]
		MTT/NCI-H1650	1.31 μM (IC_50_)	Taxol 0.04 μM (IC_50_)	[92]
		MTT/A2780	0.68 μM (IC_50_)	Taxol 0.01 μM (IC_50_)	[92]
	Tumor-related kinases inhibition	Spectrophotometric/FGFR3	0.5 μM (IC_50_)	-	[92]
		Spectrophotometric/IGF1R	0.7 μM (IC_50_)	-	[92]
		Spectrophotometric/PDGFRb	1.9 μM (IC_50_)	-	[92]
		Spectrophotometric/TRKB	1.0 μM (IC_50_)	-	[92]
		Spectrophotometric/WT	1.9 μM (IC_50_)	-	[92]
Roridin L-2 (**129**)	Cytotoxicity	MTT/HCT-116	1.73 μM (IC_50_)	Taxol 0.03 μM (IC_50_)	[92]
		MTT/HepG2	1.20 μM (IC_50_)	Taxol 0.01 μM (IC_50_)	[92]
		MTT/BGC-823	1.81 μM (IC_50_)	Taxol 0.01 μM (IC_50_)	[92]
		MTT/NCI-H1650	2.36 μM (IC_50_)	Taxol 0.04 μM (IC_50_)	[92]
		MTT/A2780	1.61 μM (IC_50_)	Taxol 0.01 μM (IC_50_)	[92]
	Tumor-related kinases inhibition	Spectrophotometric/FGFR3	0.4 μM (IC_50_)	-	[92]
		Spectrophotometric/IGF1R	0.5 μM (IC_50_)	-	[92]
		Spectrophotometric/PDGFRb	2.3 μM (IC_50_)	-	[92]
		Spectrophotometric/TRKB	1.9 μM (IC_50_)	-	[92]
		Spectrophotometric/WT	1.7 μM (IC_50_)	-	[92]
Chartarene D (**130**)	Cytotoxicity	MTT/HCT-116	1.48 μM (IC_50_)	Taxol 0.03 μM (IC_50_)	[92]
		MTT/HepG2	0.90 μM (IC_50_)	Taxol 0.01 μM (IC_50_)	[92]
		MTT/BGC-823	0.68 μM (IC_50_)	Taxol 0.01 μM (IC_50_)	[92]
		MTT/NCI-H1650	2.23 μM (IC_50_)	Taxol 0.04 μM (IC_50_)	[92]
		MTT/A2780	0.69 μM (IC_50_)	Taxol 0.01 μM (IC_50_)	[92]
	Tumor-related kinases inhibition	Spectrophotometric/FGFR3	0.1 μM (IC_50_)	-	[92]
		Spectrophotometric/IGF1R	0.1 μM (IC_50_)	-	[92]
		Spectrophotometric/PDGFRb	0.8 μM (IC_50_)	-	[92]
		Spectrophotometric/TRKB	0.7 μM (IC_50_)	-	[92]
		Spectrophotometric/WT	0.7 μM (IC_50_)	-	[92]
Roridin E (**134**)	Cytotoxicity	MTT/HCT-116	˂0.01 μM (IC_50_)	Taxol 0.03 μM (IC_50_)	[92]
		MTT/HepG2	˂0.01 μM (IC_50_)	Taxol 0.01 μM (IC_50_)	[92]
		MTT/BGC-823	˂0.01 μM (IC_50_)	Taxol 0.01 μM (IC_50_)	[92]
		MTT/NCI-H1650	˂0.01 μM (IC_50_)	Taxol 0.04 μM (IC_50_)	[92]
		MTT/A2780	˂0.01 μM (IC_50_)	Taxol 0.01 μM (IC_50_)	[92]
	Tumor-related kinases inhibition	Spectrophotometric/FGFR3	0.4 μM (IC_50_)	-	[92]
		Spectrophotometric/IGF1R	0.4 μM (IC_50_)	-	[92]
		Spectrophotometric/PDGFRb	1.4 μM (IC_50_)	-	[92]
		Spectrophotometric/TRKB	1.0 μM (IC_50_)	-	[92]
		Spectrophotometric/WT	2.1 μM (IC_50_)	-	[92]
Muconomycin B (**139**)	Cytotoxicity	MTT/HCT-116	1.32 μM (IC_50_)	Taxol 0.03 μM (IC_50_)	[92]
		MTT/HepG2	0.81 μM (IC_50_)	Taxol 0.01 μM (IC_50_)	[92]
		MTT/BGC-823	0.84 μM (IC_50_)	Taxol 0.01 μM (IC_50_)	[92]
		MTT/NCI-H1650	0.86 μM (IC_50_)	Taxol 0.04 μM (IC_50_)	[92]
		MTT/A2780	0.68 μM (IC_50_)	Taxol 0.01 μM (IC_50_)	[92]
	Tumor-related kinases inhibition	Spectrophotometric/FGFR3	0.3 μM (IC_50_)	-	[92]
		Spectrophotometric/IGF1R	0.4 μM (IC_50_)	-	[92]
		Spectrophotometric/PDGFRb	1.2 μM (IC_50_)	-	[92]
		Spectrophotometric/TRKB	0.7 μM (IC_50_)	-	[92]
		Spectrophotometric/WT	1.5 μM (IC_50_)	-	[92]
Satratoxin F (**140**)	Antibacterial	Serial dilution/Methicillin-resistant *S*. *aureus* ATCC 29213	39.0 μg/mL (MIC)	Ampicillin 10.0 μg/mL (MIC)	[73]
Satratoxin G (**142**)	Cytotoxicity	MTT/HCT-116	˂0.01 μM (IC_50_)	Taxol 0.03 μM (IC_50_)	[92]
		MTT/HepG2	˂0.01 μM (IC_50_)	Taxol 0.01 μM (IC_50_)	[92]
		MTT/BGC-823	˂0.01 μM (IC_50_)	Taxol 0.01 μM (IC_50_)	[92]
		MTT/NCI-H1650	˂0.01 μM (IC_50_)	Taxol 0.04 μM (IC_50_)	[92]
		MTT/A2780	˂0.01 μM (IC_50_)	Taxol 0.01 μM (IC_50_)	[92]
	Tumor-related kinases inhibition	Spectrophotometric/FGFR3	0.1 μM (IC_50_)	-	[92]
		Spectrophotometric/IGF1R	0.1 μM (IC_50_)	-	[92]
		Spectrophotometric/PDGFRb	0.5 μM (IC_50_)	-	[92]
		Spectrophotometric/TRKB	0.2 μM (IC_50_)	-	[92]
		Spectrophotometric/WT	0.9 μM (IC_50_)	-	[92]
Satratoxin H (**144**)	Cytotoxicity	MTT/HCT-116	˂0.01 μM (IC_50_)	Taxol 0.03 μM (IC_50_)	[92]
		MTT/HepG2	˂0.01 μM (IC_50_)	Taxol 0.01 μM (IC_50_)	[92]
		MTT/BGC-823	˂0.01 μM (IC_50_)	Taxol 0.01 μM (IC_50_)	[92]
		MTT/NCI-H1650	˂0.01 μM (IC_50_)	Taxol 0.04 μM (IC_50_)	[92]
		MTT/A2780	˂0.01 μM (IC_50_)	Taxol 0.01 μM (IC_50_)	[92]
	Tumor-related kinases inhibition	Spectrophotometric/FGFR3	˂0.1 μM (IC_50_)	-	[92]
		Spectrophotometric/IGF1R	˂0.1 μM (IC_50_)	-	[92]
		Spectrophotometric/PDGFRb	˂0.1 μM (IC_50_)	-	[92]
		Spectrophotometric/TRKB	˂0.1 μM (IC_50_)	-	[92]
		Spectrophotometric/WT	0.1 μM (IC_50_)	-	[92]
Mytoxin A (**146**)	Cytotoxicity	MTT/HCT-116	˂0.01 μM (IC_50_)	Taxol 0.03 μM (IC_50_)	[92]
		MTT/HepG2	˂0.01 μM (IC_50_)	Taxol 0.01 μM (IC_50_)	[92]
		MTT/BGC-823	˂0.01 μM (IC_50_)	Taxol 0.01 μM (IC_50_)	[92]
		MTT/NCI-H1650	˂0.01 μM (IC_50_)	Taxol 0.04 μM (IC_50_)	[92]
		MTT/A2780	˂0.01 μM (IC_50_)	Taxol 0.01 μM (IC_50_)	[92]
	Tumor-related kinases inhibition	Spectrophotometric/FGFR3	˂0.1 μM (IC_50_)	-	[92]
		Spectrophotometric/IGF1R	˂0.1 μM (IC_50_)	-	[92]
		Spectrophotometric/PDGFRb	˂0.1 μM (IC_50_)	-	[92]
		Spectrophotometric/TRKB	˂0.1 μM (IC_50_)	-	[92]
		Spectrophotometric/WT	˂0.1 μM (IC_50_)	-	[92]
Chartarutine A (**147**)	Anti-HIV virus	Luciferase/VSV-G	74.00 μM (IC_50_)	Efavirenz 0.65 μM (IC_50_)	[104]
Chartarutine B (**148**)	Anti-HIV virus	Luciferase/VSV-G	4.90 μM (IC_50_)	Efavirenz 0.65 μM (IC_50_)	[104]
Chartarutine C (**149**)	Anti-HIV virus	Luciferase/VSV-G	24.00 μM (IC_50_)	Efavirenz 0.65 μM (IC_50_)	[104]
Chartarutine D (**150**)	Anti-HIV virus	Luciferase/VSV-G	51.76 μM (IC_50_)	Efavirenz 0.65 μM (IC_50_)	[104]
Chartarutine E (**151**)	Anti-HIV virus	Luciferase/VSV-G	40.70 μM (IC_50_)	Efavirenz 0.65 μM (IC_50_)	[104]
Chartarutine F (**152**)	Anti-HIV virus	Luciferase/VSV-G	18.63 μM (IC_50_)	Efavirenz 0.65 μM (IC_50_)	[104]
Chartarutine G (**153**)	Anti-HIV virus	Luciferase/VSV-G	5.57 μM (IC_50_)	Efavirenz 0.65 μM (IC_50_)	[104]
Chartarutine H (**154**)	Anti-HIV	Luciferase/VSV-G	5.58 μM (IC_50_)	Efavirenz 0.65 μM (IC_50_)	[104]
Stachybotrysam A (**156**)	Anti-HIV virus	Luciferase/293T cells	9.3 μM (IC_50_)	Efavirenz 2.0 nM (IC_50_)	[76]
Stachybotrysam B (**157**)	Anti-HIV virus	Luciferase/293T cells	1.0 μM (IC_50_)	Efavirenz 2.0 nM (IC_50_)	[76]
Stachybotrysam C (**158**)	Anti-HIV virus	Luciferase/293T cells	9.6 μM (IC_50_)	Efavirenz 2.0 nM (IC_50_)	[76]
Atranone Q (**180**)	Cytotoxicity	MTT/MG-63	8.6 μM (IC_50_)	5-FU 10.4 μM (IC_50_)	[62]
	Antimicrobial	Broth microdilution/*S. aureus* ATCC 43300	32.0 μg/mL (MIC)	Vancomycin 0.5 μg/mL (MIC)	[61]
		Broth microdilution/*E. faecalis* ATCC 29212	16.0 μg/mL (MIC)	Vancomycin 0.5 μg/mL (MIC)	[61]
		Broth microdilution/*C. albicans* ATCC 10231	8.0 μg/mL (MIC)	Fluconazole 1.0 μg/mL (MIC)	[61]
Stachatranone B (**186**)	Antimicrobial	Broth microdilution/*A. baumannii* ATCC 19606	16.0 μg/mL (MIC)	Ceftriaxone 8 μg/mL (MIC)	[61]
Stachybotrychromene A (**193**)	Cytotoxicity	Alamar Blue assay/HepG2	73.7 μM (IC_50_)	-	[64]
Stachybotrychromene B (**194**)	Cytotoxicity	Alamar Blue assay/HepG2	28.2 μM (IC_50_)	-	[64]
*Epi*-cochlioquinone A (**199**)	Human chemokine antagonist	CCR-5	4.0 μM (IC_50_)	-	[109]
11-*O*-Methyl-epi-cochlioquinone A (**200**)	Human chemokine antagonist	CCR-5	7.0 μM (IC_50_)	-	[109]
Cyclosporin A (**209**)	Immunosuppressant	Lymphocyte murine inhibition/MLR	9.9 ng/mL (IC_50_)	-	[111]
		Mitogen suppression/ConA	21.9 ng/mL (IC_50_)	-	[111]
		Skin grafting, TGF-β1/ radioimmunoprecipitation	19.0 day (Median serivial time)/100 mg/kg orally	Vehicle treated group (Olive oil)	[111]
FR901459 A (**210**)	Immunosuppressant	Lymphocyte murine inhibition/MLR	26.8 ng/mL (IC_50_)	-	[111]
		Mitogen suppression/ConA	50.1 ng/mL (IC_50_)	-	[111]
		Skin grafting, TGF-β1/ radioimmunoprecipitation	10.0 day (Median serivial time)/100 mg/kg orally	Vehicle treated group (Olive oil)	[111]
Arthproliferin A (**211**)	Antibacterial	Serial dilution/Methicillin-resistant *S*. *aureus* ATCC 29213	78.0 μg/mL (MIC)	Ampicillin 10.0 μg/mL (MIC)	[73]
BR-011 (**214**)	Anti-HIV virus	Luciferase/VSV-G	17.90 μM (IC_50_)	Efavirenz 0.65 μM (IC_50_)	[104]

## Data Availability

Not applicable.

## Abbreviations

A-549	Human lung cancer cell line
A2780	Human ovarian cancer cell line
BGC823	Human gastric carcinoma cell line
BV2	Microglia cell line
C4-2B	Human prostatic carcinoma cell line
CCR5	Chemokine (C-C motif) receptor 5
CCK-8	Cell counting Kit 8
CD	Circular dichroism
ConA	Concanavalin A
CH_2_Cl_2_	Dichloromethane
CHO	Chinese hamster ovary cell line
CsA	Cyclosporin A
DRG	Dorsal root ganglia
EC_50_	The concentration (or dose) effective in producing 50% of the maximal response
ECD	Electronic circular dichroism
EtOAc	Ethyl acetate
FGFR3	Fibroblast growth factor receptor 3
GC-MS	Gas chromatography–mass spectrometry
HCT-116	Human colorectal carcinoma cell line
HepG2	Human hepatic cancer cell line
HIV	human immunodeficiency virus
HL-60	Human myeloid leukemia cell line:
HPLC	High performance liquid chromatography
IFN-γ	Interferon γ
IGF1R	Insulin-like growth factor 1 receptor
IL-2	Interleukin 2
MCF-7	Human breast cancer cell line
MDAMB-231	Human triple-negative breast cancer
MDA-MB-468	Epithelial, human breast cancer cell line
MG-63	Human osteosarcoma cell line
MGC803	Human gastric cancer cell line
MIC	minimum inhibitory concentrations
MIP-1α	Macrophage inflammatory protein-1 Alpha
MLR	One-way mixed lymphocyte reaction
mRNA	Messenger ribonucleic acid
MTT	(3-(4,5-Dimethylthiazol-2-yl)-2,5-diphenyltetrazolium bromide)
MVOCs	Microbial volatile organic compounds
NCI-H460	Human lung carcinoma cell line
NCI-H1655/1650	Non-small-cell lung adenocarcinoma
NRTIs	Nucleoside reverse transcriptase inhibitors
RP-18	Rephrased phase-18
PAP-1	(5-(4-Phenoxybutoxy)psoralen)
PC-12	Human pheochromocytoma neuronal cell line
PDGFRb	β-Type platelet-derived growth factor receptor
SK-N-SH	Human neuroblastoma cell line
SiO_2_CC	Silica gel column chromatography
SMMC-7721	Human hepatocellular carcinoma cell line
SW480	Human colon cancer cell line
U-2OS	Human osteosarcoma cell line
TC	Total cholesterol
TG	Total glyceride
Tie2	Tyrosine-protein kinase receptor
TRKB	Tropomyosin receptor kinase B
VSV-G	Vesicular stomatitis virus G protein
WT	Cell Division Cycle 37
