# Correction: Ibrahim et al. *Stachybotrys chartarum*—A Hidden Treasure: Secondary Metabolites, Bioactivities, and Biotechnological Relevance. *J. Fungi* 2022, *8*, 504

**DOI:** 10.3390/jof12110801

**Published:** 2026-10-23

**Authors:** Sabrin R. M. Ibrahim, Hani Choudhry, Amer H. Asseri, Mahmoud A. Elfaky, Shaimaa G. A. Mohamed, Gamal A. Mohamed

**Affiliations:** 1 Department of Chemistry, Preparatory Year Program, Batterjee Medical College, Jeddah 21442, Saudi Arabia; 2 Department of Pharmacognosy, Faculty of Pharmacy, Assiut University, Assiut 71526, Egypt; 3 Biochemistry Department, Faculty of Science, King Abdulaziz University, Jeddah 21589, Saudi Arabia; 4 Center for Artificial Intelligence in Precision Medicines, King Abdulaziz University, Jeddah 21589, Saudi Arabia; 5 Department of Natural Products and Alternative Medicine, Faculty of Pharmacy, King Abdulaziz University, Jeddah 21589, Saudi Arabia; 6 Faculty of Dentistry, British University, El Sherouk City, Suez Desert Road, Cairo 11837, Egypt

**Text: Corrections and Clarifications**

The authors request the following corrections and clarifications to the original publication [1]. After re-examining the article and verifying it against the cited primary literature, the identified issues are limited to isolated typographical and numbering errors. The corrections do not affect the reported chemical structures, biological data, scientific interpretation, or overall conclusions of the review.

**1. Bistachybotrysin K (83): Unit Correction**

In the narrative text describing Bistachybotrysin K (**83**), “IC_50_ ranged from 1.1 to 4.7 mM” should be corrected to “IC_50_ ranged from 1.1 to 4.7 µM”. The numerical values are unchanged. The corrected unit is consistent with Table 2 and the cited primary source (Ref. 82).

**2. Stachybochartin G: Compound-Number Correction**

F1839-A and Stachybochartin G are distinct compounds. F1839-A is compound **51**, whereas Stachybochartin G is compound **15**, as correctly listed in Table 1. Accordingly, the duplicate 19th row of Table 2, incorrectly designated “Stachybochartin G (**51**)”, should be deleted, while the existing entry for Stachybochartin G (**15**) should be retained. The retained IC_50_ values of 5.6 µM against MDA-MB-231 and 4.5 µM against U2-OS are consistent with Table S1 of the Supporting Information for Ref. 70. In the related narrative text, “Stachybochartin G (**51**)” should be corrected to “Stachybochartin G (**15**)”. F1839-A (**51**) remains unchanged.

**3. Compound 9: Stereochemical Descriptor**

The published stereochemical descriptor “2R/3S/5S/8R/9R/0S” contains a typographical omission and should be corrected to “2R/3S/5S/8R/9R/10S”. The digit “1” was inadvertently omitted from “10S”. This correction does not alter the corresponding chemical structure or its interpretation.

The authors state that the scientific conclusions are unaffected. This correction was approved by the Academic Editor. The original publication has also been updated.
